# Supplementary material for: A PP2A-B55-Mediated Crosstalk between TORC1 and TORC2 Regulates the Differentiation Response in Fission Yeast
Source: Curr Biol. 2017 Jan 23;27(2):175–88. doi: 10.1016/j.cub.2016.11.037 (PMC5266790; doi:10.1016/j.cub.2016.11.037)
Supplement: Document S1. Figures S1–S6 and Supplemental Experimental Procedures [file mmc1.pdf]

Current Biology, Volume *27*

Supplemental Information

A PP2A-B55-Mediated Crosstalk between TORC1 and TORC2 Regulates the Differentiation Response in Fission Yeast

Ruth Martín, Marina Portantier, Nathalia Chica, Mari Nyquist-Andersen, Juan Mata, and Sandra Lopez-Aviles

A

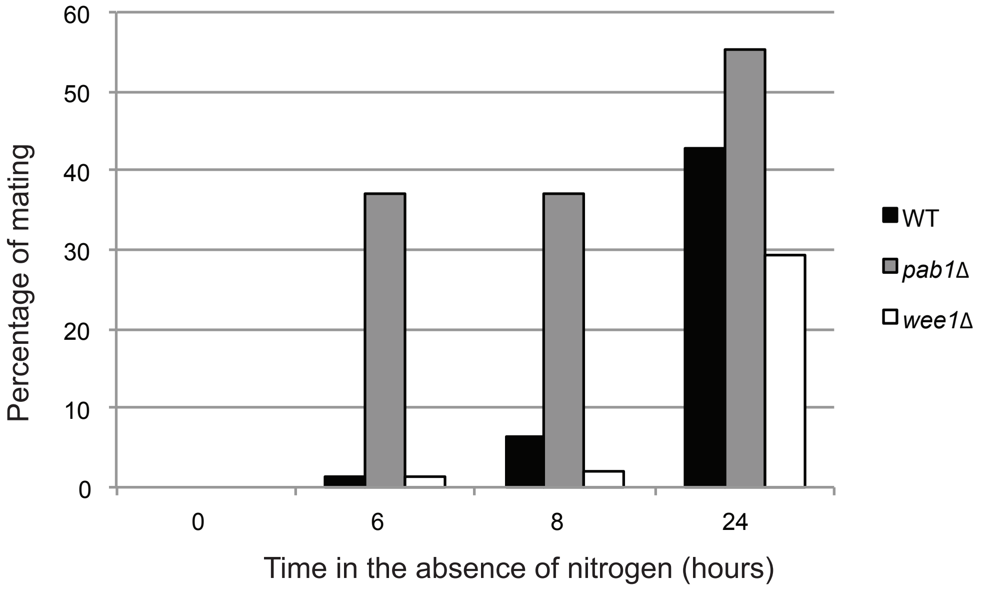

B

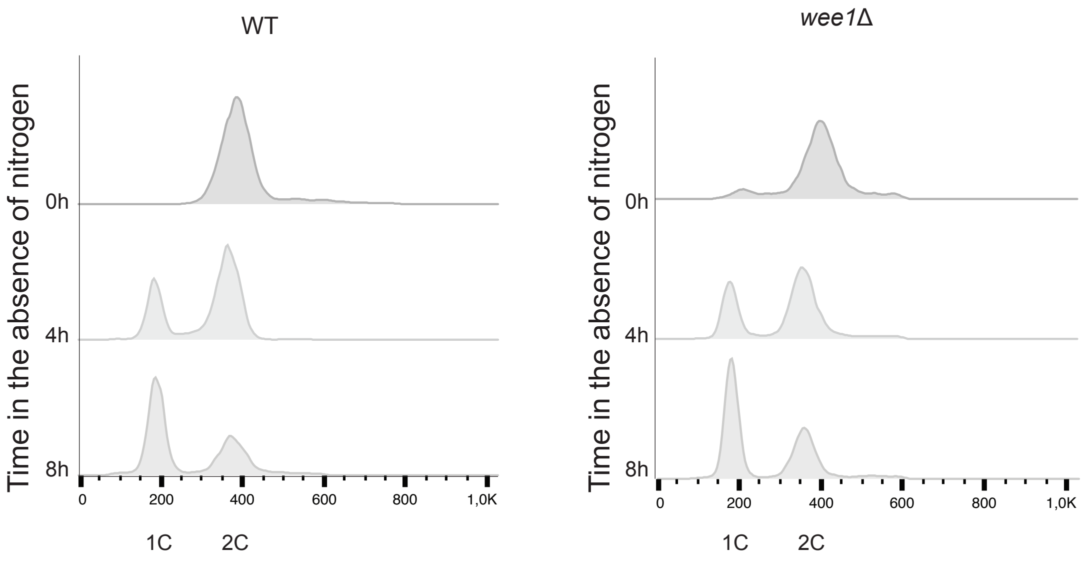

Figure S1 (related to Figure 1): *wee1*Δ cells display reduced conjugation efficiency.

A, homotallic WT, *pab1*Δ and *wee1*Δ cells were incubated at 25ºC in the absence of nitrogen and their mating ability was determined at 0 h, 6 h, 8 h and 24 h.
B, FACS analysis of the DNA content of the cells in A.

A

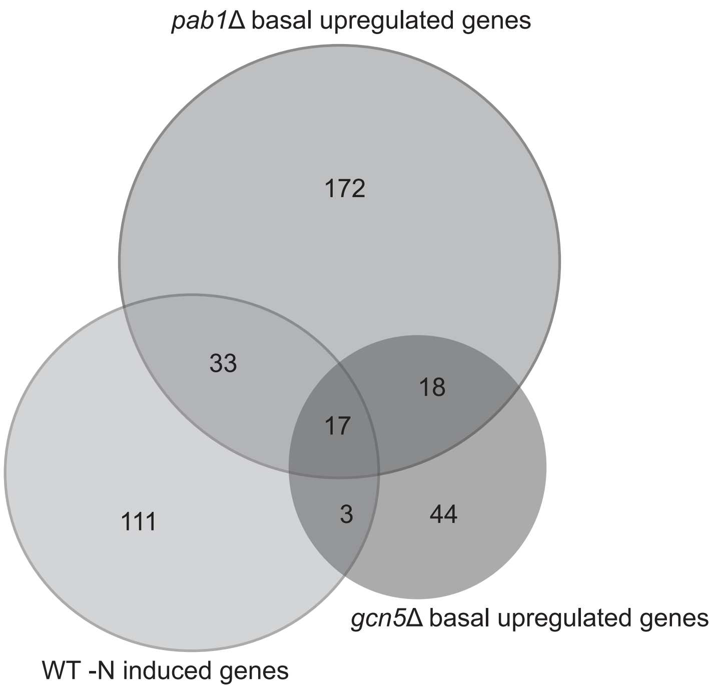

B

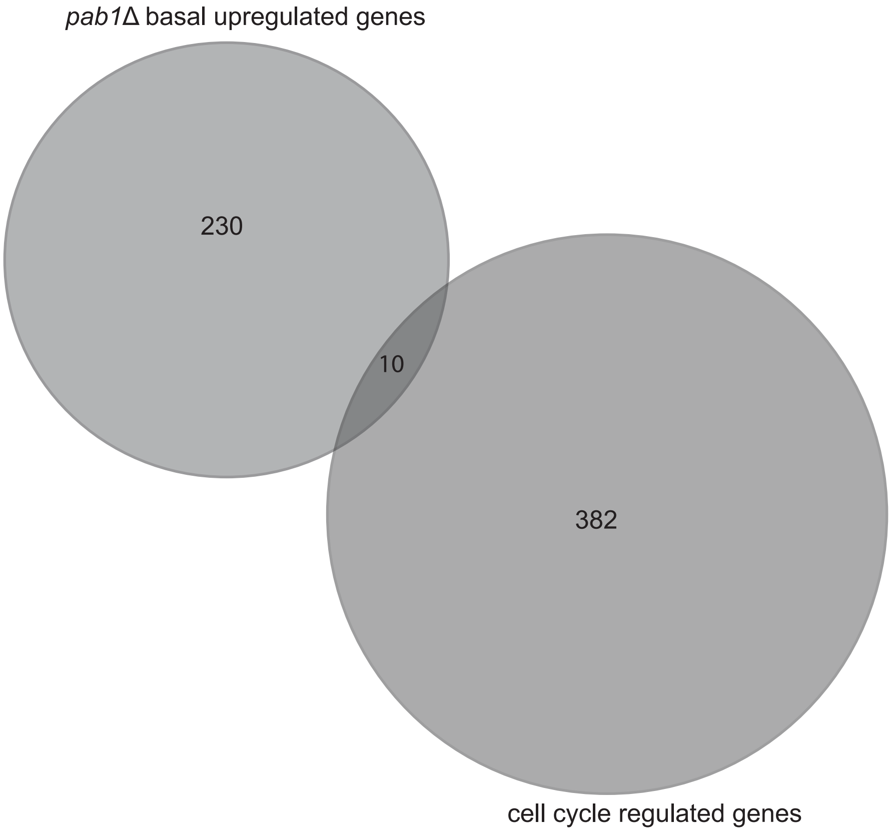

Figure S2 (related to Figure 2): The set of *pab1*Δ upregulated genes significantly overlaps with *gcn5*Δ upregulated genes but not with cell cycle regulated genes.

A, Venn diagram illustrating theoverlap between *pab1*Δ and *gcn5*Δ upregulated gene list in basal conditions (p value = $1.971867e^{-25}$). WT -N induced genes are also shown. **B**, Venn diagram illustrating the overlap between *pab1*Δ upregulated genes in basal conditions and cell cycle regulated gene list (p value = 0.99).

A

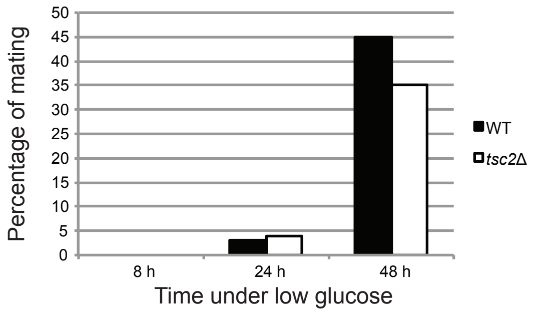

B

	cell concentration								
	5x10^{6} cells/ml			50x10^{6} cells/ml			100x10^{6} cells/ml		
	4 h	24 h	48 h	4 h	24 h	48 h	4 h	24 h	48 h
WT	0	42,7	40,9	0	38	34,2	0	8,5	9
pab1Δ	22,2	60,3	50,33	31,2	60,7	63	42,5	59	56,6
tsc2Δ	0	0	0	0	0	0,6	0	0,6	1,3
pab1Δ tsc2Δ	6,8	12,3	7,4	8,5	16,6	26,1	10,2	13,3	19,4

Percentage of mating (%)

C

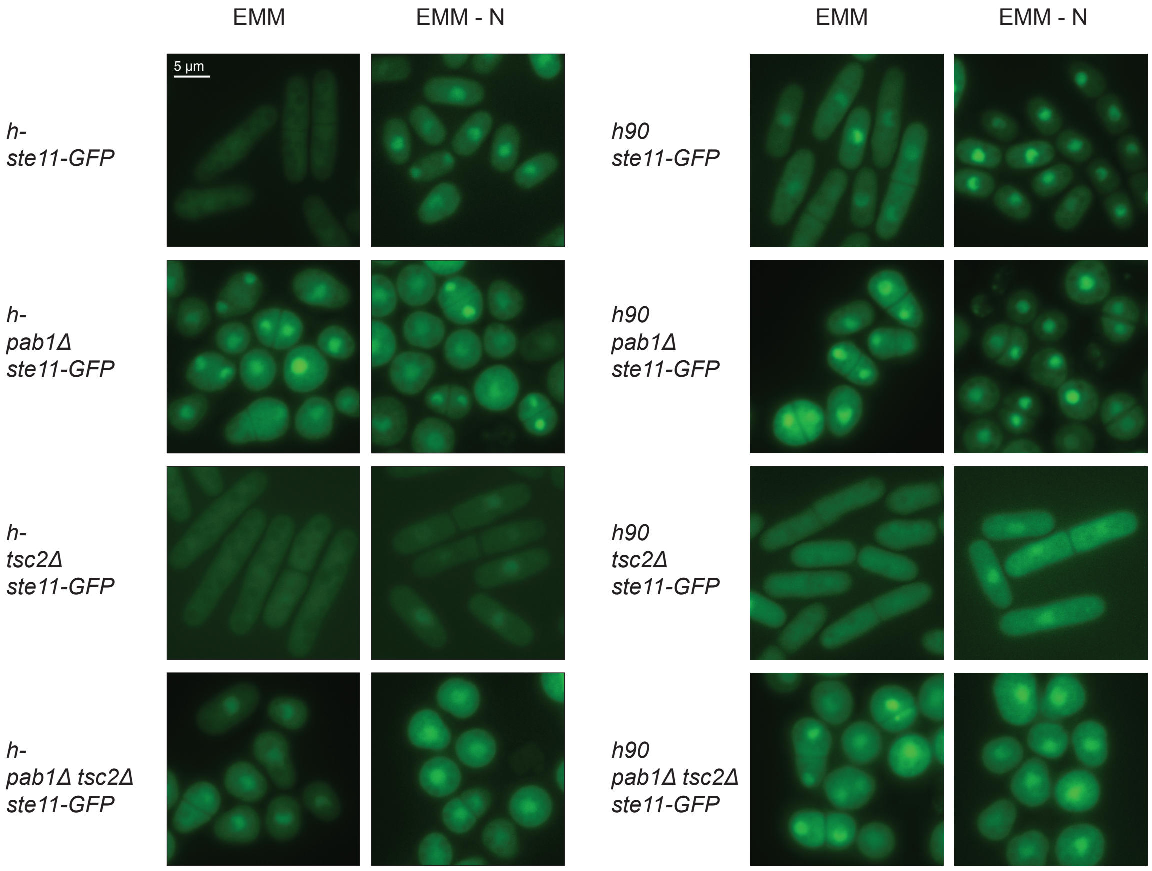

Figure S3 (related to Figure 3): *tsc2Δ* cells mating efficiency under low glucose and rescue of the sexual differentiation defect by deletion of *pab1.*

A, *tsc2Δ* cells show WT conjugation efficiency under low glucose conditions. Homotallic WT and *tsc2Δ* cells were incubated at 25ºC in the presence of 0.1% glucose (supplemented with 3% glycerol) and their mating ability was determined at 8 h, 24 h, and 48 h.

B, The mating efficiency of the *pab1Δ tsc2Δ* mutant is enhanced by high cellular concentration. Homotallic WT, *pab1Δ*, *tsc2Δ* and *pab1Δ tsc2Δ* cells were incubated at 25ºC in the absence of nitrogen at different cellular concentrations and their mating ability was determined at 4 h, 24 h and 48 h.

C, The defect in Ste11 accumulation of *tsc2Δ* mutants is rescued by *pab1* deletion. Heterotallic and homotallic WT, *pab1Δ*, *tsc2 Δ* and *pab1Δ tsc2Δ* cells expressing endogenous levels of Ste11-GFP were incubated at 32ºC in the absence of nitrogen for 4h. Control cells were incubated in EMM for the same period of time.

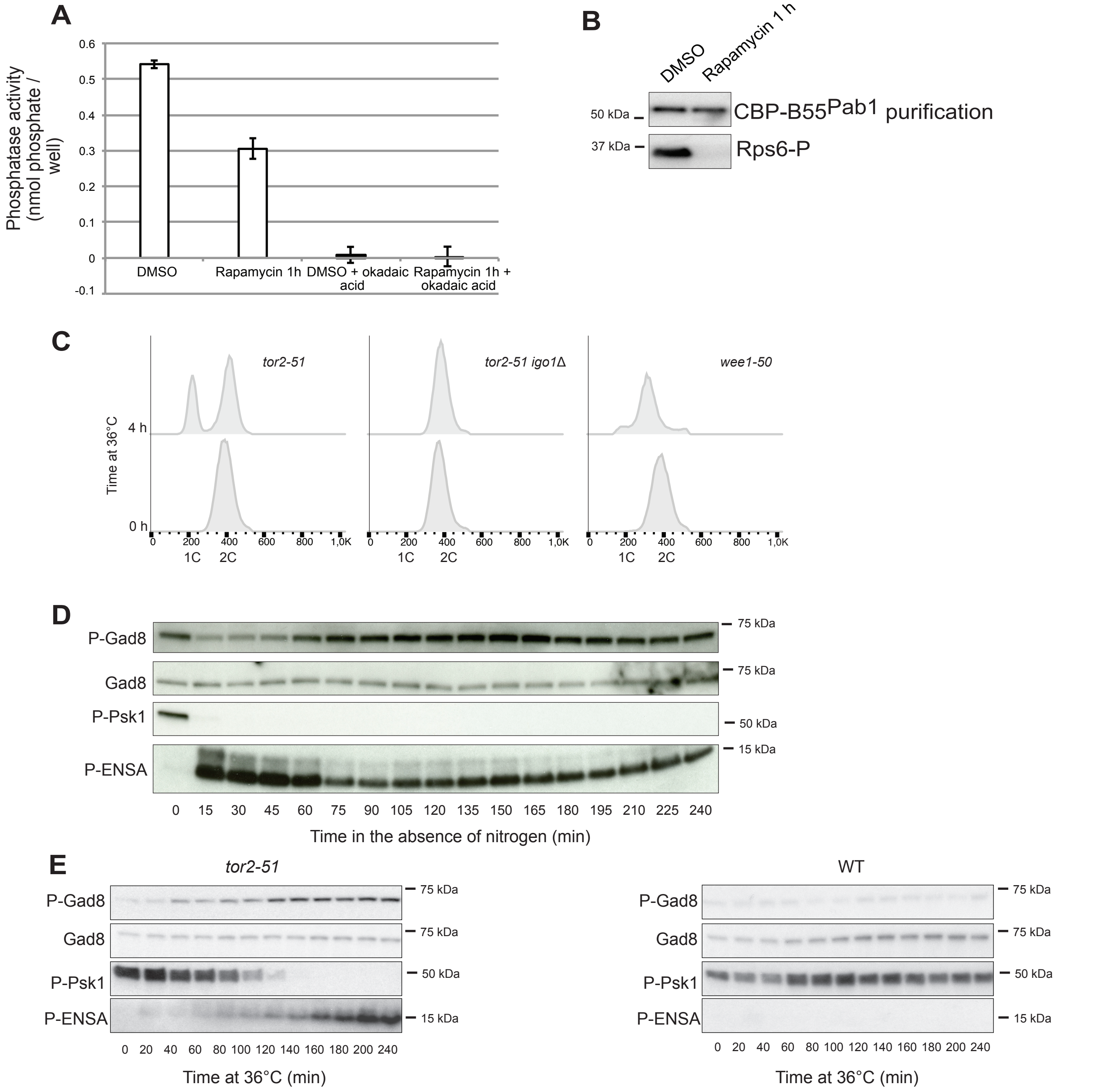

Figure S4 (related to Figure 4): TORC1 inhibition promotes inactivation of PP2A-B55 and this results in increased Gad8 Ser546 phosphorylation.

A, TAP-B55^{Pab1} was purified from cells treated with either DMSO or Rapamycin (0.2 µg/ml) for 1 h and used in a phosphatase assay against a synthetic serine phosphopeptide (DLDVPIPGRFDRRVS(PO3)VAAE). Released phosphate was used as a measure of the phosphatase activity present in the sample, and its concentration was determined based on the absorbance at 620 nm upon reaction with the malachite green reagent. Treatment with okadaic acid completely abrogated the release of phosphate, indicating that the activity observed was PP2A specific. Mean and SEM of two independent biological replicates are shown.

B, Upper panel: B55^{Pab1} present in the samples used in the phosphatase assay was detected by western blot against its amino-terminal CBP-tag. Lower panel: TORC1 activity present in the DMSO and rapamycin treated cultures was determined by western blot against P-Rps6 using TCA extracts.

C, FACS analysis of the DNA content of *tor2-51*, *tor2-51 igo1*Δ and *wee1-50* cells grown at 25°C and incubated at 36°C for 4 h.

D, Homotallic WT cells were incubated at 25°C in the absence of nitrogen and samples were collected at the indicated time points. Gad8 phosphorylation at Ser546 and Igo1 phosphorylation at Ser64 (P-ENSA) were detected by western blot. Psk1 phosphorylation was used as readout of TORC1 activity. Total Gad8 served as loading control.

E, Homotallic *tor2-51* and WT cells were grown at 25°C and then shifted to 36°C in order to inactivate TORC1. Samples were collected at the indicated time points. Gad8 phosphorylation at Ser546 and Igo1 phosphorylation at Ser64 (P-ENSA) were detected by western blot. Psk1 phosphorylation was used as readout of TORC1 activity. Total Gad8 served as loading control.

A

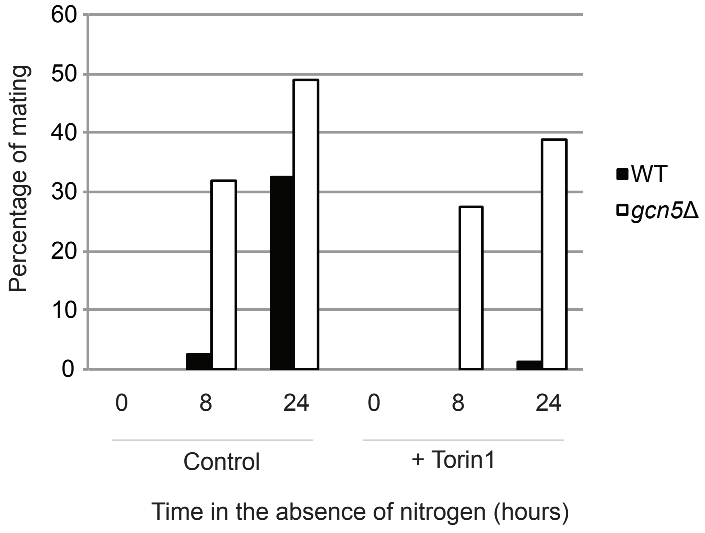

B

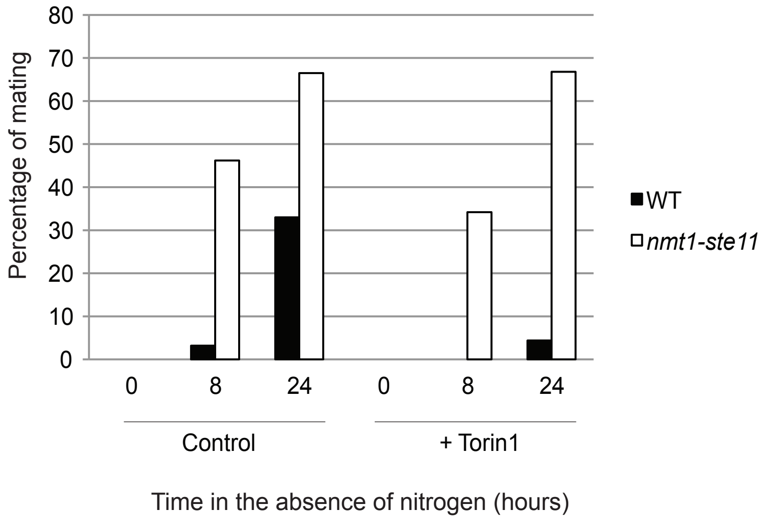

Figure S5 (related to Figure 5): Torin1 does not inhibit mating in a *gcn5*Δ strain or in a strain overexpressing *ste11*.

A, Homotallic WT and *gcn5*Δ cells were incubated at 25ºC in the absence of nitrogen in the presence or in the absence of Torin1 (25 µM) and their mating ability was determined at the indicated time points (as described in Experimental Procedures).

B, Homotallic WT and *nmt1-ste11* cells were grown at 25ºC in the absence of thiamine for 18 h, before they were washed and incubated in the absence of nitrogen in the presence or in the absence of Torin1 (25 µM). Their mating ability was subsequently determined at the indicated time points (as described in Experimental Procedures)

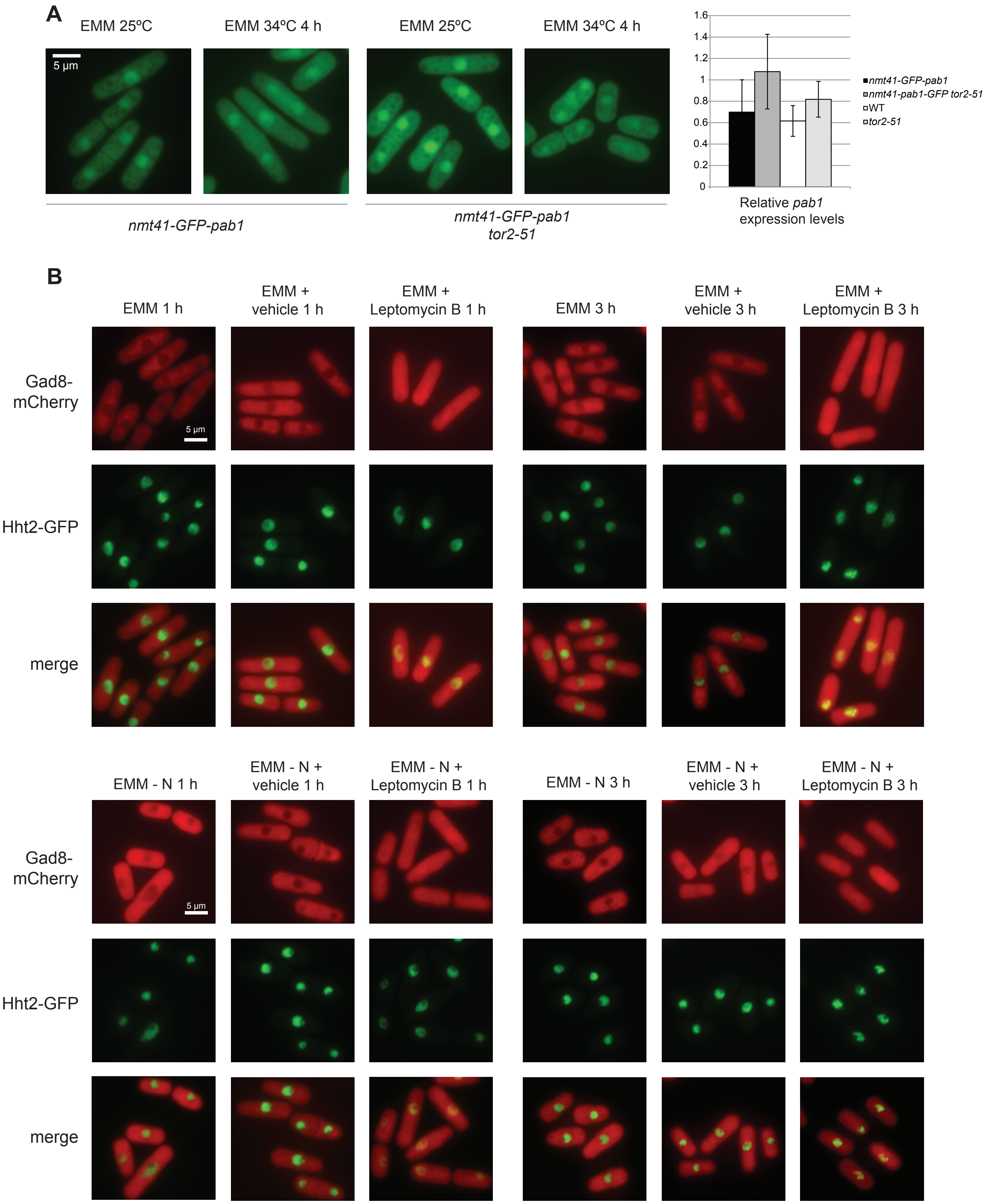

Figure S6 (related to Figure 6): In vivo localization of B55^{Pab1} and of Gad8.

A, Pab1 localizes to the nucleus and cytoplasm and its localization does not change upon Tor2 inactivation.

nmt41-GFP-pab1 and *nmt41-GFP-pab1 tor2-51* cells grown in EMM were shifted to the restrictive temperature of 34ºC for 4 h in order to inactivate Tor2. Control cells were kept at 25ºC for the same period of time. mRNA expression of *pab1* in these cells was analyzed and compared to the endogenous expression in a WT and in a *tor2-51* strain grown in EMM at 25ºC. Expression is relative to actin and was determined by qPCR. Mean and SEM of three biological replicates is shown.

B, Gad8 localizes primarily to the cytoplasm, but treatment with leptomycin B reveals nuclear-cytoplasmic shuttling of a fraction of Gad8.

gad8-mCherry hht2-GFP cells grown at 25ºC in EMM, were split and incubated either in the absence of nitrogen (EMM-N) or in fresh EMM for the indicated times (1 h or 3 h). Gad8 localization under these conditions was assessed upon treatment with leptomycin B or vehicle (for the same period of time). Histone H2 localization served as a marker for the nuclear compartment.

Supplemental Experimental Procedures

Plasmid and strain construction

For the construction of the *nmt41-3PK-miniAID-pab1* strain, a new pFA6a plasmid (pFA6a-KanMX6-p41nmt1-3PK-miniAID) was generated. Briefly, the 3PK tag followed by the miniAID module was amplified by PCR from existing plasmids [S1], adding a 6 Gly linker downstream of the miniAID and overhangs of 15 bp both at the 5' and 3' end of the cassette. These 15 bp overhangs bore homology to the regions upstream and downstream of the tag in a pFA6a-KanMX6-p41nmt1-3HA plasmid. This plasmid was also amplified by PCR using primers matching the 15bp regions of homology, therefore excluding the 3HA tag. The final plasmid was obtained by recombination of these two fragments using a recombination-mediated cloning kit (In-Fusion® Cloning Kit, Takara-Clontech).

For the construction of the *gad8Ser546Ala* strain, the *gad8* gene was first deleted using an Ura4 cassette, in a *ura4-D18* background. The whole genomic sequence spanning from 635 upstream the start codon to 661 bp downstream of the stop codon and comprising the Ser546 to Ala substitution (AGC→GCC) was synthesized by GenScript and cloned in a pUC57 backbone. Digestion of the plasmid with BamHI and PstI released a 3087 bp fragment containing the genomic sequence of *gad8* (Ser546Ala) flanked by the regions upstream of the start codon and downstream of the stop codon. This fragment was then used to transform the *gad8*Δ::*ura4* strain and clones that had exchanged the Ura4 cassette by the mutated allele of *gad8* were selected based on their ability to grow on FOA plates supplemented with Uracil. The presence of the Ser546Ala was confirmed by sequencing.

For the construction of the *gad8-5flag Ser546Ala* allele, genomic DNA was obtained from a Gad8-5flag tagged strain (*gad8-5flag::KanMX6*). This DNA was used as template in two different PCRs. The first PCR amplified a region starting 465 nt upstream of the original stop codon and finishing 8bp downstream of the Ser546 codon. The reverse primer in this PCR introduced a mutation in the Ser546 codon, so that the final PCR product contained the Ser to Ala mutation (AGC→GCC). Similarly, the second PCR produced a fragment that started 9bp before the Ser546 codon, and that finished 374 bp downstream of the 5flag-KanMX6 cassette. As for the first PCR the primer matching the Ser546 codon introduced the mutation to Ala. These two PCRs were then used as template in a tandem PCR reaction together with the forward primer of the first reaction and the reverse primer of the second in order to generate a single cassette now containing the Ser to Ala mutation, the 5flag tag and the kanMX6 module as well as extensive regions of homology to Gad8 ORF and 3' UTR upstream and downstream of the tag and antibiotic resistance. This final PCR was then used to transform a wild type strain. Positive clones were subsequently sequenced to confirm the presence of the mutation.

For the construction of the *fkh2Ser321*Ala strain, the *fkh2* gene was first deleted in an *ura4-D18* background using an Ura4 cassette. The whole genomic sequence spanning from 560 bp upstream the start codon to 400 bp downstream of the stop codon and comprising the Ser321 to Ala substitution (TCC→GCC) was synthesized by GenScript and cloned in a pUC18 backbone. Digestion of the plasmid with AseI released a 3076 bp fragment containing the genomic sequence of *fkh2* (Ser321Ala) flanked by the regions upstream of the start codon and downstream of the stop codon. This fragment was then used to transform the *fkh2*Δ::*ura4* strain and clones that had exchanged the Ura4 cassette by the mutated allele of *fkh2* were selected based on their ability to grow on FOA plates supplemented with Uracil. The presence of the Ser321Ala was confirmed by sequencing.

The NTAP-Pab1 strain was obtained by transformation of *pab1*Δ::KanMX6 *leu1-32* with a pJK148-Ppab1-NTAP-pab1-pab1 3'UTR that had been digested with Sty1 for integration at *leu1*. To obtain the plasmid, the different fragments corresponding to the promoter region and 5'UTR (starting 977 bp upstream of the start codon and finishing at the start codon), the NTAP tag (including a 9 Gly linker) and the genomic sequence and 3'UTR of *pab1* (starting at the start codon and finishing 714 bp downstream of the stop codon) were obtained by PCR. Genomic DNA was used as template to amplify the promoter and the genomic sequence followed by 3'UTR of *pab1*. For the amplification of the NTAP we used a pcDNA3-NTAP plasmid as template. Each PCR reaction was carried out with primers that introduced 15 bp of homology to the adjacent region in the final plasmid. These three PCR reactions were cloned by recombination using a recombination-mediated cloning kit (In-Fusion® Cloning Kit, Takara-Clontech) in a pJK148 empty vector that had been linearized with NotI and SacI.

Strains used in this study

Figure 1		
167	h^{90} WT	Lab stock
343	h^{90} *pab1Δ::kanMX6*	Lab stock
363	h^{90} *par1Δ::kanMX6*	Lab stock
696	h^{90} *dis2Δ::ura4*	Lab stock
Figure 2		
167	h^{90} WT	Lab stock
343	h^{90} *pab1Δ::kanMX6*	Lab stock
678	h^{90} *tor1Δ::kanMX6*	This study
Figure 3		
557	h^{90} *kanMX6::P3nmt1::tor2*	S. Moreno
551	h^{90} *kanMX6::P3nmt1::tor2 pab1Δ::kanMX6*	This study
167	h^{90} WT	Lab stock
343	h^{90} *pab1Δ::kanMX6*	Lab stock
350	h^{90} *tsc2Δ::kanMX6*	This study
554	h^{90} *tsc2Δ::kanMX6 pab1Δ::hphMX6*	This study
147	*h+* WT	Lab stock
411	*h+ pab1Δ::kanMX6*	Lab stock
857	*h- tsc2Δ::kanMX6 pab1Δ::hphMX6*	This study
848	*h- tsc2Δ::kanMX6*	This study
Figure 4		
167	h^{90} WT	Lab stock
343	h^{90} *pab1Δ::kanMX6*	Lab stock
561	h^{90} *kanMX6::P3nmt1::pab1*	Lab stock
594	*h- ade6::ade6+-Padh15-skp1-AtTIR1-2NLS-natMX6-Padh15-skp1-OsTIR1*	Lab stock (Originally from H. Masukata)
593	*h- kanMX6::P41nmt1::3PK-miniAID-pab1 ade6::ade6+-Padh15-skp1-AtTIR1-2NLS-natMX6-Padh15-skp1-OsTIR1*	This study
650	*h- cdc10-V50 kanMX6::P41nmt1::3PK-miniAID-pab1 ade6::ade6+-Padh15-skp1-AtTIR1-2NLS-natMX6-Padh15-skp1-OsTIR1*	This study
222	h^{90} *tor2-51::ura4*	S. Moreno
660	h^{90} *tor2-51::ura4 igo1Δ::hphMX6*	This study
825	h^{90} *wee1-50*	P. Nurse
Figure 5		
222	h^{90} *tor2-51::ura4*	S. Moreno
660	h^{90} *tor2-51::ura4 igo1Δ::hphMX6*	This study
714	h^{90} *tor2-51::ura4 igo1Δ::hphMX6 flag-ryh1QL*	This study (*flag-ryh1QL* allele originally from K. Shiozaki)
167	h^{90} WT	Lab stock
343	h^{90} *pab1Δ::kanMX6*	Lab stock
900	h^{90} *gad8S546A*	This study
908	h^{90} *gad8S546A pab1Δ::hphMX6*	This study
767	h^{90} *fkh2S321A*	This study

773	*h^{90} fkh2S321A pab1Δ::hphMX6*	This study
Figure 6		
841	*h- 3flag-tor1::hphMX6 gad8-5flag::kanMX6 leu1-32*	This study (*3flag-tor1* allele originally from M. Yanagida)
588	*h- 3flag-tor1::hphMX6 gad8-5flag::kanMX6 pab1Δ::kanMX6 leu1-32::leu1::Ppab1::TAP-pab1*	This study
647	*h+ sin1-13myc::natMX6 kanMX6::P41nmt1::3PK-miniAID-pab1 ade6::ade6+-Padh15-skp1-AtTIR1-2NLS-natMX6-Padh15-skp1-OsTIR1*	This study
637	*h- sin1-13myc::natMX6 gad8-5flag::kanMX6 kanMX6::P41nmt1::3PK-miniAID-pab1 ade6::ade6+-Padh15-skp1-AtTIR1-2NLS-natMX6-Padh15-skp1-OsTIR1*	This study
887	*h- sin1-13myc::natMX6 kanMX6::P3nmt1::3HA-pab1*	This study
888	*h+ sin1-13myc::natMX6 gad8-5flag::hphMX6 kanMX6::P3nmt1::3HA-pab1*	This study
732	*h- leu1-32::leu1::Ppab1::TAP-pab1 pab1Δ::kanMX6 Δpar1::hphMX6*	This study
148	*h-* WT	Lab stock
586	*h+ gad8-5flag::kanMX6*	K. Shiozaki
587	*h+ gad8-5flag::kanMX6 pab1Δ::kanMX6*	This study
870	*h- gad8S546A-5flag::kanMX6*	This study
897	*h- gad8S546A-5flag::kanMX6 pab1Δ::hphMX6*	This study
932	*gad8K259R-5flag::hphMX6*	This study (*gad8K259R* allele originally from J. Petersen)
Figure S1		
167	*h^{90}* WT	Lab stock
343	*h^{90} pab1Δ::kanMX6*	Lab stock
568	*h^{90} wee1Δ::ura4 ura4-D18*	This study
Figure S2		
167	*h^{90}* WT	Lab stock
343	*h^{90} pab1Δ::kanMX6*	Lab stock
Figure S3		
167	*h^{90}* WT	Lab stock
350	*h^{90} tsc2Δ::kanMX6*	This study
343	*h^{90} pab1Δ::kanMX6*	Lab stock
554	*h^{90} tsc2Δ::kanMX6 pab1Δ::hphMX6*	This study
849	*h- ste11-GFP::kanMX6*	This study
850	*h- ste11-GFP::kanMX6 tsc2Δ::kanMX6*	This study
851	*h^{90} ste11-GFP::kanMX6 tsc2Δ::kanMX6*	This study
852	*h^{90} ste11-GFP::kanMX6*	This study
853	*h- ste11-GFP::kanMX6 pab1Δ::hphMX6*	This study
854	*h^{90} ste11-GFP::kanMX6 pab1Δ::hphMX6*	This study
855	*h- ste11-GFP::kanMX6 tsc2Δ::kanMX6 pab1Δ::hphMX6*	This study
856	*h^{90} ste11-GFP::kanMX6 tsc2Δ::kanMX6 pab1Δ::hphMX6*	This study
Figure S4		
584	*h- leu1-32::leu1::Ppab1::TAP-pab1 pab1Δ::kanMX6*	This study
167	*h^{90}* WT	Lab stock

222	h^{90} *tor2-51::ura4*	S. Moreno
660	h^{90} *tor2-51::ura4 igo1Δ::hphMX6*	This study
825	h^{90} *wee1-50*	P. Nurse
Figure S5		
167	h^{90} WT	Lab stock
475	h^{90} *gcn5Δ::kanMX6*	D. Helmlinger
859	h^{90} *kanMX6::P3nmt1::ste11*	This study
Figure S6		
705	*h- kanMX6::P41nmt1::GFP-pab1*	Lab stock
731	*h+ tor2-51::ura4 kanMX6::P41nmt1::GFP-pab1*	Lab stock
905	*h+ gad8-mcherry::kanMX6 hht2-GFP:ura4*	This study

Drugs

A 100 µM stock solution of Okadaic acid (Sigma, 07885) was prepared in DMSO and used at final concentrations 1 nM and 10 nM. A 7.5 mM stock solution of Torin1 (R & D systems, 4247) was prepared in DMSO and used at a final concentration of 25 µM. Rapamycin (Sigma, R0395) was resuspended in DMSO at a concentration of 1mg/ml and used at a final concentration of 0.2 µM. A 500 mM stock solution of 1-Naphthaleneacetic acid potassium salt (ChemCruz Biochemicals, SC-229803) was prepared in water and added to a final concentration of 500 µM.

Leptomycin B (Sigma, L2913) was added at a final concentration of 100 ng/ml. Control cells were treated with the same volume of vehicle (Methanol:water (7:3)).

Protein extraction and Immunoblotting

Samples were collected by filtration and washed in STOP buffer (150 mM NaCl, 50 mM NaF, 10 mM EDTA, 1 mM sodium azide) before freezing them in liquid nitrogen. Protein extracts were prepared using the TCA method [S2]. 30-50 µg of total protein was loaded in Criterion™ TGX™ Any Kd™ gels (BioRad) and transferred to PVDF membranes using a semi-dry blotting system (Trans-Blot® Turbo™ from BioRad). Antibody solutions were prepared in TBS-Tween (0.1%) containing 5% BSA (for all antibodies against phosphorylated residues except for Gad8-Ser546-P) or 5% non-fat milk (in all other instances). Western blots were developed using Amersham ECL western blotting detection reagents.

Antibodies

Polyclonal anti Gad8S546-P and anti Gad8 (directed against the last 20 aminoacids of Gad8) antibodies were produced in rabbit by Eurogentec and used in 1:250-1:1000 dilutions for Western blotting.

The following commercial antibodies were used for WB: anti-PSTAIR (Abcam) 1:1000, anti-phospho S/T AKT substrate (PAS) (Cell Signaling) 1:1000; anti-phospho-S6K (T389) mouse monoclonal antibody (clone # 1A5) (Cell Signaling) 1:1000 was used to detect phosphorylated Psk1; anti phospho-p44/p42 (Cell Signaling) 1:1000 was used to detect phosphorylated Spk1; anti-CBP (GenScript) 1:1000; anti-Myc 9E10 (SIGMA) 1:1000; anti-Flag (SIGMA) 1:1000; anti-HA 12CA5 (Roche) 1:1000; anti-GST (Abcam) 1:1000; anti-V5 (AbD Serotec) 1:1000; anti-PP2A C subunit (Millipore) 1:1000; Phospho-ENSA (Ser67) (Cell Signaling) 1:1000; HRP-conjugated anti mouse IgG (SIGMA) 1:10000 and HRP-conjugated anti rabbit IgG (SIGMA) 1:10000. For IP experiments the anti-Flag, anti-Myc, anti-protein A (SIGMA) antibodies were used in 1:250 dilution. In order to avoid IgG detection in IP experiments, Rabbit and Mouse Trueblot® (Rockland) secondary antibodies were used.

Immunopurifications

150 ml of exponentially growing cells ($\sim 10^9$ cells) were collected by filtration, and pellets were washed in STOP buffer or in TBS (for IPs used in kinase and phosphatase assays) before freezing them in liquid nitrogen. Native protein extraction was performed in IP Buffer (20 mM Tris-HCl pH 8, 140 mM KCl, 1.8 mM MgCl2, 0.1% NP40, 1 mM PMSF, 1 cOmplete™ mini protease inhibitors (Roche)/5 ml buffer, 1 phosSTOP™ tablet (Roche)/10 ml buffer). Additional phosphatase inhibitors (50 mM NaF, 0,2 mM sodium ortovanadate, 0,02 µM mycrocystin-LR, 1 mM sodium β-glycerophosphate, 10 mM sodium pyrophosphate) were included in the buffer for IPs to be used in kinase assays. For the TAP-purification of PP2A-$B55^{Pab1}$ all phosphatase inhibitors were omitted.

Cells were broken using a FastPrep®-24 equipped with a CryoPrep™ adapter for cryogenic lysis. For all the IPs, 10 mg of whole cell extract were incubated 1 h at 4 ºC with 75 µl of Dynabeds ® Pan Mouse IgG (Invitrogen) which had been incubated overnight with the corresponding antibody in a 1:250 dilution. In the case of Tandem Affinity purifications (TAP) the beads were incubated with anti-protein A. The beads were then washed 4 times in Wash Buffer (20 mM Tris-HCl pH 8, 140 mM KCl, 1.8 mM MgCl2, 0.04% NP40, 10% glycerol) containing 1 mM PMSF and 4 times with Wash Buffer containing 1 mM DTT. The beads were finally resuspended in laemmli loading buffer (for co-inmunopurification experiments) or in the corresponding kinase or phosphatase buffer. In the case of TAP purifications, the beads were resuspended in wash buffer containing DTT and 20 U AcTEV (Invitrogen) was added for 1 h RT in order to release the CBP-tagged protein from the protein A.

Kinase assays

The purification of Gad8-5flag was done in IP Buffer containing phosphatase inhibitors as explained above.

The Gad8-Fkh2 assay was performed according to Laor D et al. [S3]. Briefly, Gad8-5flag IP was incubated with GST-Fkh2 recombinant fragment (100 ng/µl) purified from *E. coli* in the kinase buffer (2 mM DTT, 2.5 mM Mg acetate, 20 mM Tris HCl pH 8, 2.5 mM MgCl2, 1 mM ATP) and incubated 10 min at 30 ºC. Reactions were stopped by boiling the samples for 5 min in laemmli loading buffer. Western blot against PAS was used to detect phosphorylated recombinant Fkh2 and served as readout of the Gad8 activity present in the samples.

Phosphatase assays

For phosphatase assays, CBP-B55^{Pab1} was purified from a *par1*Δ strain (in order to avoid competition for the scaffolding and catalytic subunit) in IP Buffer without phosphatase inhibitors and Gad8-5flag (used as the substrate) was purified from a *pab1*Δ strain with IP Buffer containing phosphatase inhibitors. Prior to the assay, the Gad8-5flag beads were washed in Wash Buffer to ensure removal of phosphatase inhibitors before finally resuspending them in Phosphatase Buffer (50 mM Tris pH 7.5, 10 mM MgCl2, 0.02 % Brij-35, 1 mg/ml BSA). The assay was performed by adding Gad8-5flag IP to eluted CBP-B55^{Pab1} in phosphatase buffer (pre-treated with okadaic acid at the indicated concentration for 15 min in the specified cases) and incubated for 30 min at 30 ºC. The reactions were terminated as explained before. Western blot against Gad8-Ser546-P was used to determine the extent of dephosphorylation of Gad8 by PP2A-B55^{Pab1}.

A malachite green-based colorimetric assay (DuoSet® IC activity assay from R&D systems) was used to determine the activity of PP2A-B55^{Pab1} upon Tor2 inhibition. The assay was performed using CBP-B55^{Pab1} IP according to the manufacturer instructions.

RNA extraction and quantitative PCR (qPCR)

For qPCR and RNAseq experiments 20 ml samples were collected by centrifugation and pellets were washed in DEPC-treated water before freezing them in liquid nitrogen.

Total RNA preparation was performed with MasterPure™ Yeast RNA Purification Kit (Epicentre) following the manufacturer instructions. For qPCR experiments, 1 µg of RNA was used for cDNA synthesis using SuperScript® III Reverse Transcriptase (Invitrogen). qPCR was performed with the corresponding oligos, mei2: AAGAAACTCCCACTGCTGCT and CTGGAGATGATTCAGTGCGT and act1: CAAATCCAACCGTGAGAAGA and CATCACCAGAGTCCAAGACG with SYBR® Select Master Mix (Applied Biosystems). Analysis was done using the ΔΔCt method.

RNAseq

The RNA quality was assessed with Bioanalyzer. The construction of libraries (TruSeq stranded prep) and sequencing (NextSeq 500 75 bp SR H.O) was performed by Norwegian Seq Center http://www.sequencing.uio.no/services/

Reads were aligned to the S. pombe genome using Tophat 2 version 2.1.0 [S4] and Bowtie 2 version 2.2.6 [S5]. The following parameters were used: --no-novel-juncs --no-novel-indels --min-anchor-length 20 --b2-very-fast --read-mismatches 2 --min-intron-length 29 --max-intron-length 819 --max-multihits 1. A gff3 file downloaded from Pombase (ASM294v2.28) [S6] was used as a source of splice junctions. The numbers of reads per feature were quantified using a custom-written Perl script. Determination of differentially expressed genes was performed using the Bioconductor package DEseq [S7]. A threshold if adjusted p values of 0.01 was used. Enrichment analysis was performed using ANGELI [S8]. Raw data files have been deposited in ArrayExpress [S9] with accession number E-MTAB-4106.

The p value of the overlapping genes between lists was calculated with R i386 3.2.2 (hypergeometric test).

Supplemental References

S1. Kubota, T., Nishimura, K., Kanemaki, M. T., and Donaldson, A. D. (2013). The Elg1 replication factor C-like complex functions in PCNA unloading during DNA replication. Mol. Cell *50*, 273–280.

S2. Foiani, M., Marini, F., Gamba, D., Lucchini, G., and Plevani, P. (1994). The B subunit of the DNA polymerase alpha-primase complex in Saccharomyces cerevisiae executes an essential function at the initial stage of DNA replication. Mol. Cell. Biol. *14*, 923–933.

S3. Laor, D., Cohen, A., Pasmanik-Chor, M., Oron-Karni, V., Kupiec, M., and Weisman, R. (2014). Isp7 is a novel regulator of amino acid uptake in the TOR signaling pathway. Mol. Cell. Biol. *34*, 794–806.

S4. Kim, D., Pertea, G., Trapnell, C., Pimentel, H., and Kelley, R. (2013). TopHat2: accurate alignment of transcriptomes in the presence of insertions, deletions and gene fusions. Genome Biology *14,* R36.

S5. Langmead, B., and Salzberg, S. L. (2012). Fast gapped-read alignment with Bowtie 2. Nat. Methods *9*, 357–359.

S6. McDowall, M. D., Harris, M. A., Lock, A., Rutherford, K., Staines, D. M., Bähler, J., Kersey, P. J., Oliver, S. G., and Wood, V. (2015). PomBase 2015: updates to the fission yeast database. Nucleic Acids Res. *43*, D656–61.

S7. Anders, S., and Huber, W. (2010). Differential expression analysis for sequence count data. Genome Biology *11*, R106.

S8. Bitton, D. A., Schubert, F., Dey, S., Okoniewski, M., Smith, G. C., Khadayate, S., Pancaldi, V., Wood, V., and Bähler, J. (2015). AnGeLi: A Tool for the Analysis of Gene Lists from Fission Yeast. Front. Genet. *6*, 76.

S9. Rustici, G., Kolesnikov, N., Brandizi, M., Burdett, T., Dylag, M., Emam, I., Farne, A., Hastings, E., Ison, J., Keays, M., et al. (2013). ArrayExpress update--trends in database growth and links to data analysis tools. Nucleic Acids Res. *41*, D987–90.
