# Supplementary material for: A PP2A-B55-Mediated Crosstalk between TORC1 and TORC2 Regulates the Differentiation Response in Fission Yeast
Source: Curr Biol. 2017 Jan 23;27(2):175–88. doi: 10.1016/j.cub.2016.11.037 (PMC5266790; doi:10.1016/j.cub.2016.11.037)
Supplement: Table S1. Enrichment Analysis of Genes Upregulated in pab1Δ versus WT in Basal Conditions, Related to Figure 2 [file mmc2.pdf]

Table S1. Enrichment analysis of genes upregulated in *pab1Δ* vs wt in basal conditions. Related to Figure 2.

Category Name	External ID	GeneSet Name	over represented/under represented	List Frequency	Background Frequency	Corrected pvalue	Genes In Common/Interacting Gene Pair
**GO Biological Process**	GO:0000003	reproduction	Enriched	18.18 (42/231)	6.34 (326/5135)	3.74462e-08	G10.04C;SPAC31G5.09C;SPBC409.03;SPBC1718.02;SPBC16E9.08;SPAC27D7.03C;SPBC1711.02;SPAC222.15;SPCC1183.12;SPBC29A10.02;SPAC11E3.06;SPBC21D10.06C;SPAPB8E5.05;SPCC1442.01;SPAC458.04C;SPAC4G9.05;SPCC1795.06;SPBC23G7.09;SPAC11H11.04;SPBC4.01;SPAC24C9.15C;SPAC1565.04C;SPAC13C5.03;SPBP8B7.28C;SPAC22F3.12C;SPBC428.07;SPAC2F7.06C;SPBC405.05;SPAC1F8.05;SPAC3F10.10C;SPAC23C4.07;SPBC119.14;SPBC1347.03;SPAC10F6.12C;SPAC6G9.16C;SPBC337.08C;SPBC27.03
**GO Biological Process**	GO:0032005	signal transduction involved in conjugation with cellular fusion	Enriched	4.32 (10/231)	0.56 (29/5135)	3.21177e-05	SPBPJ4664.03;SPAC513.03;SPAC31G5.09C;SPAC11E3.06;SPCC1442.01;SPCC1795.06;SPAC11H11.04;SPAC1565.04C;SPAC22F3.12C;SPAC3F10.10C
**GO Biological Process**	GO:0022414	reproductive process	Enriched	10.82 (25/231)	3.62 (186/5135)	6.85163e-05	SPBPJ4664.03;SPCC1235.12C;SPAC513.03;SPBC1778.04;SPAC31G5.09C;SPBC409.03;SPBC1711.02;SPCC1183.12;SPAC11E3.06;SPBC29A10.02;SPBC21D10.06C;SPAPB8E5.05;SPCC1442.01;SPCC1795.06;SPBC23G7.09;SPAC11H11.04;SPBC4.01;SPAC1565.04C;SPAC13C5.03;SPAC22F3.12C;SPAC2F7.06C;SPAC3F10.10C;SPAC23C4.07;SPBC1347.03;SPAC10F6.12C
**GO Biological Process**	GO:0019953	sexual reproduction	Enriched	9.95 (23/231)	3.17 (163/5135)	7.96681e-05	11.02;SPCC1183.12;SPBC29A10.02;SPAC11E3.06;SPBC21D10.06C;SPAPB8E5.05;SPCC1442.01;SPCC1795.06;SPBC23G7.09;SPAC11H11.04;SPBC4.01;SPAC1565.04C;SPAC13C5.03;SPAC22F3.12C;SPAC3F10.10C;SPAC23C4.07;SPBC1347.03;SPAC10F6.12C
**GO Biological Process**	GO:0044703	multi-organism reproductive process	Enriched	9.95 (23/231)	3.17 (163/5135)	7.96681e-05	11.02;SPCC1183.12;SPBC29A10.02;SPAC11E3.06;SPBC21D10.06C;SPAPB8E5.05;SPCC1442.01;SPCC1795.06;SPBC23G7.09;SPAC11H11.04;SPBC4.01;SPAC1565.04C;SPAC13C5.03;SPAC22F3.12C;SPAC3F10.10C;SPAC23C4.07;SPBC1347.03;SPAC10F6.12C
**GO Biological Process**	GO:0000747	conjugation with cellular fusion	Enriched	7.79 (18/231)	2.08 (107/5135)	9.98943e-05	10.06C;SPAPB8E5.05;SPCC1442.01;SPCC1795.06;SPBC23G7.09;SPAC11H11.04;SPBC4.01;SPAC1565.04C;SPAC13C5.03;SPAC22F3.12C;SPAC3F10.10C;SPAC23C4.07;SPAC10F6.12C
**GO Biological Process**	GO:0000749	response to pheromone involved in conjugation with cellular fusion	Enriched	3.89 (9/231)	0.50 (26/5135)	0.000103463	SPAC31G5.09C;SPAC11E3.06;SPBC21D10.06C;SPCC1442.01;SPCC1795.06;SPAC11H11.04;SPAC1565.04C;SPAC22F3.12C;SPAC10F6.12C
**GO Biological Process**	GO:0019236	response to pheromone	Enriched	4.32 (10/231)	0.64 (33/5135)	0.000103463	SPAC31G5.09C;SPAC11E3.06;SPBC21D10.06C;SPCC1442.01;SPCC1795.06;SPAC11H11.04;SPAC1565.04C;SPAC22F3.12C;SPAC3F10.10C;SPAC10F6.12C
**GO Biological Process**	GO:0051704	multi-organism process	Enriched	9.956 (23/231)	3.23 (166/5135)	0.000103463	11.02;SPCC1183.12;SPBC29A10.02;SPAC11E3.06;SPBC21D10.06C;SPAPB8E5.05;SPCC1442.01;SPCC1795.06;SPBC23G7.09;SPAC11H11.04;SPBC4.01;SPAC1565.04C;SPAC13C5.03;SPAC22F3.12C;SPAC3F10.10C;SPAC23C4.07;SPBC1347.03;SPAC10F6.12C
**GO Biological Process**	GO:0000746	conjugation	Enriched	7.79 (18/231)	2.10 (108/5135)	0.000107651	10.06C;SPAPB8E5.05;SPCC1442.01;SPCC1795.06;SPBC23G7.09;SPAC11H11.04;SPBC4.01;SPAC1565.04C;SPAC13C5.03;SPAC22F3.12C;SPAC3F10.10C;SPAC23C4.07;SPAC10F6.12C
**GO Biological Process**	GO:0044764	multi-organism cellular process	Enriched	7.79 (18/231)	2.14 (110/5135)	0.000140382	SPBPJ4664.03;SPAC513.03;SPAC31G5.09C;SPBC1711.02;SPAC11E3.06;SPBC21D10.06C;SPAPB8E5.05;SPCC1442.01;SPCC1795.06;SPBC23G7.09;SPAC11H11.04;SPBC4.01;SPAC1565.04C;SPAC13C5.03;SPAC22F3.12C;SPAC3F10.10C;SPAC23C4.07;SPAC10F6.12C
**GO Biological Process**	GO:0051321	meiotic cell cycle	Enriched	11.68 (27/231)	4.49 (231/5135)	0.000323077	SPAC6G9.13C;SPCC1235.12C;SPBC1778.04;SPAC25G10.04C;SPAC31G5.09C;SPBC409.03;SPBC1718.02;SPBC16E9.08;SPAC27D7.03C;SPAC222.15;SPCC1183.12;SPAC11E3.06;SPBC29A10.02;SPAC458.04C;SPAC4G9.05;SPBC23G7.09;SPAC24C9.15C;SPBP8B7.28C;SPBC428.07;SPBC405.05;SPAC1F8.05;SPAC3F10.10C;SPBC119.14;SPBC1347.03;SPAC6G9.16C;SPBC337.08C;SPBC27.03

**GO Biological Process**	GO:0071444	cellular response to pheromone	Enriched	3.89 (9/231)	0.58 (30/5135)	0.000363032	SPAC31G5.09C;SPAC11E3.06;SPBC21D10.06C;SPCC1442.01;SPCC1795.06;SPAC11H11.04;SPAC1565.04C;SPAC22F3.12C;SPAC10F6.12C
**GO Biological Process**	GO:0000750	pheromone-dependent signal transduction involved in conjugation with cellular fusion	Enriched	3.03 (7/231)	0.37 (19/5135)	0.000858307	SPAC31G5.09C;SPAC11E3.06;SPCC1442.01;SPCC1795.06;SPAC11H11.04;SPAC1565.04C;SPAC22F3.12C
**GO Biological Process**	GO:0007186	G-protein coupled receptor signaling pathway	Enriched	3.466 (8/231)	0.54 (28/5135)	0.00159615	SPAC31G5.09C;SPAC11E3.06;SPCC1442.01;SPCC1795.06;SPAC11H11.04;SPAC1565.04C;SPAC22F3.12C;SPAC3F10.10C
**GO Biological Process**	GO:0007166	cell surface receptor signaling pathway	Enriched	3.46 (8/231)	0.64 (33/5135)	0.0049694	SPAC31G5.09C;SPAC11E3.06;SPCC1442.01;SPCC1795.06;SPAC11H11.04;SPAC1565.04C;SPAC22F3.12C;SPAC3F10.10C
**GO Biological Process**	GO:0010033	response to organic substance	Enriched	5.19 (12/231)	1.49 (77/5135)	0.00857751	SPAC31G5.09C;SPAC11E3.06;SPBC21D10.06C;SPCC1442.01;SPBC3E7.02C;SPCC1795.06;SPAC11H11.04;SPAC1565.04C;SPBP8B7.28C;SPAC22F3.12C;SPAC3F10.10C;SPAC10F6.12C
**Gene Expression**		Meiosis sporulation module	Enriched	30.73 (71/231)	5.25 (270/5135)	1.07499e-34	5A10.05C;SPCC338.18;SPBC1778.04;SPCC285.07C;SPCC794.02;SPCC1906.04;SPAC1F5.09C;SPBC146.11C;SPCC1393.07C;SPCC1281.08;SPBC16E9.08;SPBC15D4.12C;SPBC359.06;SPAC4F10.17;SPAC27D7.03C;SPAC1002.19;SPAC22H10.13;SPAC222.15;SPBC1198.14C;SPBC19F8.06C;SPAC1A6.06C;SPAC29A4.12C;SPAC1F8.01;SPBC29A10.02;SPBC19C7.04C;SPBC21D10.06C;SPCC162.04C;SPAPB8E5.05;SPBC16A3.13;SPAC6C3.05;SPAC4G9.07;SPBC3E7.02C;SPAC4G9.05;SPAC8F11.05C;SPCC1795.06;SPAC1F8.04C;SPAC25H1.09;SPBC1711.11;SPAC11H11.04;SPBC839.06;SPAC24C9.15C;SPBC4.01;SPCC74.09;SPAC167.06C;SPCC1259.14C;SPAC13G6.08;SPCC24B10.14C;SPCC1235.13;SPCC338.04;SPAC22F3.12C;SPCC1739.15;SPBC428.07;SPBC4C3.08;SPAC22A12.02C;SPCC188.12;SPAP27G11.08C;SPAC1F8.05;SPCC970.11C;SPBC119.14;SPAC23C4.07;SPBC1347.03;SPAPJ691.02;SPCC162.10;SPAC1A6.08C;SPBC14C8.05C;SPBC27.03
**Gene Expression**		Reproduction module	Enriched	24.24 (56/231)	5.74 (295/5135)	6.30409e-19	SPBPJ4664.03;SPAC869.07C;SPCC70.04C;SPAC6B12.03C;SPAC29B12.13;SPAC513.03;SPAC15A10.05C;SPCC338.18;SPAC22G7.11C;SPCC285.07C;SPAC12B10.13;SPBC887.16;SPCC794.02;SPAC1F5.09C;SPAC31G5.09C;SPCC1393.07C;SPBC359.06;SPAC4F10.17;SPAC977.16C;SPAC27D7.03C;SPAC1002.19;SPAC22H10.13;SPBC1198.14C;SPAC29A4.12C;SPAC688.03C;SPAC11E3.06;SPCC338.12;SPBC19C7.04C;SPBC21D10.06C;SPCC162.04C;SPAPB8E5.05;SPCC1442.01;SPAC1565.03;SPCC1795.06;SPCC162.06C;SPAC1F8.04C;SPAC13F5.07C;SPBC1711.11;SPAC869.08;SPAC18G6.01C;SPBC4.01;SPBC800.14C;SPAC167.06C;SPAC1565.04C;SPAC513.02;SPAC22F3.12C;SPCC1739.15;SPAC2F7.06C;SPCC188.12;SPAC1F8.05;SPAC3F10.10C;SPCC285.04;SPCC970.11C;SPCC162.10;SPAC6G9.16C;SPAPJ691.02
**Gene Expression**		Nitrogen depletion delayed meiotic genes	Enriched	8.65 (20/231)	0.72 (37/5135)	2.0764e-15	SPBPJ4664.03;SPAC513.03;SPAC1F5.09C;SPAC31G5.09C;SPAC977.16C;SPAC27D7.03C;SPBC1711.02;SPAC11E3.06;SPBC21D10.06C;SPAPB8E5.05;SPCC1442.01;SPAC1565.03;SPCC1795.06;SPAC11H11.04;SPBC4.01;SPAC1565.04C;SPAC22F3.12C;SPAC3F10.10C;SPAC10F6.12C;SPCC162.10
**Gene Expression**		Induced Dbr1 deletion	Enriched	13.859 (32/231)	2.41 (124/5135)	4.71572e-14	SPAC31G5.21;SPCPB16A4.07;SPAC11D3.01C;SPBC30B4.09;SPCC70.04C;SPAC6B12.03C;SPAPJ695.02;SPCC1906.04;SPAC5H10.11;SPAC4F10.17;SPAC1002.19;SPAC5H10.04;SPAC29A4.12C;SPBC19C7.04C;SPAC4G9.07;SPBC3E7.02C;SPAC458.04C;SPAC8F11.05C;SPAC25H1.09;SPAC11H11.04;SPBC839.06;SPAC167.06C;SPCC1259.14C;SPAC977.18;SPCC1235.13;SPAC4H3.08;SPAC513.02;SPCC737.04;SPAC2F7.06C;SPAC1F8.05;SPBC1347.03;SPAPJ691.02

**Gene Expression**		Nitrogen depletion total meiotic genes	Enriched	15.15 (35/231)	4.05 (208/5135)	9.18233e-10	SPBPJ4664.03;SPCC70.04C;SPAC6B12.03C;SPAC29B12.13;SPAC513.03;SPAC25B8.18;SPCC338.18;SPAC12B10.13;SPAC1F5.09C;SPAC31G5.09C;SPAC977.16C;SPAC27D7.03C;SPBC1711.02;SPAC688.03C;SPBC13A2.04C;SPAC11E3.06;SPCC338.12;SPBC21D10.06C;SPAC1556.04C;SPAPB8E5.05;SPCC1442.01;SPAC1565.03;SPCC1795.06;SPAC1F8.04C;SPAC11H11.04;SPBC4.01;SPBC800.14C;SPAC167.06C;SPAC1565.04C;SPAC22F3.12C;SPAC3F10.10C;SPCC285.04;SPAC10F6.12C;SPAPJ691.02;SPCC162.10
**Gene Expression**		Lowly expressed	Enriched	20.77 (48/231)	7.57 (389/5135)	7.7465e-09	SPAC6G9.13C;SPAC869.07C;SPAC869.09;SPCC1235.12C;SPAC6B12.03C;SPCC622.04;SPAC10F6.15;SPCC338.18;SPBC887.16;SPBC146.11C;SPCC1281.08;SPBC15D4.12C;SPAC4F10.17;SPBC1711.02;SPAC222.15;SPBC19F8.06C;SPAC869.06C;SPAC5H10.04;SPAC6C3.05;SPCC622.03C;SPAC4G9.07;SPAC458.04C;SPAC4G9.05;SPAC8F11.05C;SPAC1F8.04C;SPBC23G7.09;SPAC869.08;SPAC11H11.04;SPAC13C5.04;SPAC22G7.03;SPBC839.06;SPCC74.09;SPBC13G1.15C;SPAC5H10.02C;SPCC1259.14C;SPBC23G7.11;SPAC13G6.08;SPAC513.04;SPAC4H3.08;SPCC737.04;SPBC428.07;SPAC2F7.06C;SPAC22A12.02C;SPBC4C3.08;SPAP27G11.08C;SPAC23C4.07;SPBC1347.03;SPBC27.03
**Gene Expression**		Atf31 targets	Enriched	8.22 (19/231)	1.57 (81/5135)	2.53585e-07	SPAC11D3.01C;SPAC869.07C;SPAC869.09;SPCC1235.12C;SPAC15A10.05C;SPAC22G7.11C;SPCC1281.08;SPAC4F10.17;SPBC19F8.06C;SPAC869.06C;SPBC3E7.02C;SPAC869.08;SPAC15A10.07;SPCC1450.08C;SPCC74.09;SPBC3H7.08C;SPAC5H10.02C;SPCC1259.14C;SPAC4H3.08
**Gene Expression**		Caffeine and Rapamycin induced	Enriched	16.01 (37/231)	5.60 (288/5135)	5.02985e-07	SPAC869.07C;SPCC70.04C;SPBPB2B2.18;SPAC29B12.13;SPAC513.03;SPAC15A10.05C;SPBPB21E7.04C;SPCC794.02;SPBPB21E7.01C;SPCC1393.07C;SPAC27D7.03C;SPAC1002.19;SPAC22H10.13;SPAC806.07;SPAC688.03C;SPBC13A2.04C;SPAC11E3.06;SPCC338.12;SPBC19C7.04C;SPAC1556.04C;SPBC3E7.02C;SPAC1F8.04C;SPBC1711.11;SPAC869.08;SPAC13C5.04;SPBC800.14C;SPAC24C9.15C;SPAC167.06C;SPBC23G7.11;SPAC4H3.08;SPAC513.02;SPCC737.04;SPAC2F7.06C;SPAC20H4.11C;SPCC285.04;SPAPJ691.02;SPBC337.08C
**Gene Expression**		Sporulation module	Enriched	11.68 (27/231)	3.58 (184/5135)	4.46828e-06	SPAC11D3.01C;SPAC869.07C;SPAC869.09;SPCC1235.12C;SPAC6B12.03C;SPAC15A10.05C;SPCC338.18;SPAC22G7.11C;SPCC794.02;SPCC1906.04;SPCC1281.08;SPAC4F10.17;SPBC19F8.06C;SPAC869.06C;SPAC5H10.04;SPBC3E7.02C;SPAC869.08;SPAC15A10.07;SPCC74.09;SPBC3H7.08C;SPAC5H10.02C;SPCC1259.14C;SPBC23G7.11;SPAC4H3.08;SPAC513.02;SPBC428.07;SPAC2F7.06C
**Gene Expression**		Induced red1 mutant	Enriched	6.49 (15/231)	1.22(63/5135)	8.88669e-06	SPAC6G9.13C;SPAC29B12.13;SPAC25G10.04C;SPCC1393.07C;SPBC1718.02;SPAC222.15;SPAC5H10.04;SPBC29A10.02;SPAC458.04C;SPAC22G7.03;SPBC4.01;SPAP27G11.08C;SPAC23C4.07;SPBC119.14;SPAC1A6.08C
**Gene Expression**		Induced Pab2 mutant exon intron signal	Enriched	6.06 (14/231)	1.09 (56/5135)	1.25023e-05	SPAC6G9.13C;SPBPJ4664.03;SPCC70.04C;SPBPB2B2.18;SPAC513.03;SPBC29A3.07C;SPAC222.15;SPBC29A10.02;SPAPB8E5.05;SPAC4G9.05;SPBC4.01;SPBC428.07;SPAP27G11.08C;SPBC119.14
**Gene Expression**		Middle meiotic genes	Enriched	22.07 (51/231)	10.67(548/5135)	3.23574e-05	SPAC10F6.15;SPAC15A10.05C;SPBC1778.04;SPCC285.07C;SPCC794.02;SPBC21B10.11;SPCC1906.04;SPBC146.11C;SPCC1281.08;SPBC16E9.08;SPBC15D4.12C;SPBC4C3.04C;SPAC806.07;SPAC1B3.20;SPBC19F8.06C;SPAC1A6.06C;SPAC29A4.12C;SPCC162.04C;SPBC16A3.13;SPCC622.03C;SPAC4G9.07;SPBC3E7.02C;SPAC6B12.06C;SPAC4G9.05;SPAC8F11.05C;SPAC25H1.09;SPBC1711.11;SPCC1450.08C;SPBC839.06;SPAC24C9.15C;SPCC74.09;SPCC1259.14C;SPAC13G6.08;SPCC24B10.14C;SPCC1235.13;SPCC338.04;SPCC1739.15;SPBC428.07;SPAC20H4.11C;SPAC22A12.02C;SPBC4C3.08;SPCC188.12;SPAC1F8.05;SPAC23C4.07;SPCC970.11C;SPBC1347.03;SPAC1A6.08C;SPAC3H1.03;SPBC14C8.05C;SPBC337.08C;SPBC27.03

**Gene Expression**		Ste11 targets	Enriched	6.06 (14/231)	1.18 (61/5135)	3.66347e-05	SPCC338.18;SPAC1F5.09C;SPAC31G5.09C;SPCC1393.07C;SPBC359.06;SPAC27D7.03C;SPAC11E3.06;SPCC1442.01;SPAC18G6.01C;SPBC4.01;SPAC13C5.03;SPAC1565.04C;SPAC22F3.12C;SPCC162.10
**Gene Expression**		Early meiotic genes	Enriched	6.92 (16/231)	1.86 (96/5135)	0.000394237	SPAC6G9.13C;SPAC25G10.04C;SPCC1393.07C;SPBC1718.02;SPBC359.06;SPAC22H10.13;SPAC222.15;SPBC1198.14C;SPAC1F8.01;SPBC29A10.02;SPBC19C7.04C;SPAC6C3.05;SPAC458.04C;SPAC13C5.03;SPAP27G11.08C;SPBC119.14
**Gene Expression**		Induced rnc1 mutant	Enriched	6.06 (14/231)	1.49 (77/5135)	0.000543585	SPAC513.03;SPAC15A10.05C;SPCC338.18;SPBPB21E7.04C;SPAC31G5.09C;SPBPB21E7.01C;SPAC27D7.03C;SPAC11E3.06;SPBC19C7.04C;SPCC1442.01;SPCC1795.06;SPAC13F5.07C;SPAC1565.04C;SPAC22F3.12C
**Gene Expression**		Nitrogen depletion continuous meiotic genes	Enriched	5.19 (12/231)	1.32 (68/5135)	0.00282282	SPCC70.04C;SPAC6B12.03C;SPAC29B12.13;SPAC25B8.18;SPCC338.18;SPAC12B10.13;SPAC688.03C;SPBC13A2.04C;SPCC338.12;SPAC1F8.04C;SPAC167.06C;SPAPJ691.02
**Gene Expression**		Induced usp102 mutant	Enriched	3.89 (9/231)	0.87 (45/5135)	0.00849793	SPAC29B12.13;SPBPB21E7.04C;SPAC25G10.04C;SPAPB24D3.07C;SPCC338.12;SPBC19C7.04C;SPBC3E7.02C;SPAC4H3.08;SPCC737.04
**Phenotypes (FYPO)**	FYPO:0000051	abnormal meiosis	Enriched	7.35 (17/231)	2.20 (113/5135)	0.00075804	SPAC6G9.13C;SPBC1778.04;SPAC25G10.04C;SPBC1718.02;SPAC27D7.03C;SPAC222.15;SPBC29A10.02;SPAC458.04C;SPAC13C5.03;SPBP8B7.28C;SPBC428.07;SPBC405.05;SPAC1F8.05;SPBC1347.03;SPAC6G9.16C;SPBC337.08C;SPBC27.03
**Phenotypes (FYPO)**	FYPO:0003179	decreased intragenic meiotic recombination	Enriched	3.03 (7/231)	0.44 (23/5135)	0.00314642	SPAC6G9.13C;SPAC25G10.04C;SPBC1718.02;SPAC222.15;SPBC29A10.02;SPBC119.14;SPAC23C4.07
**Phenotypes (FYPO)**	FYPO:0000679	developmental process phenotype	Enriched	9.52 (22/231)	3.79(195/5135)	0.0035524	SPAC6G9.13C;SPCC1235.12C;SPBC1778.04;SPAC31G5.09C;SPBC409.03;SPBC146.11C;SPBC1718.02;SPAC27D7.03C;SPCC1183.12;SPBC29A10.02;SPAC11E3.06;SPBC21D10.06C;SPAC458.04C;SPBC23G7.09;SPAC11H11.04;SPAC1565.04C;SPAC13C5.03;SPAC3F10.10C;SPAC23C4.07;SPBC1347.03;SPAC10F6.12C;SPBC337.08C
**Phenotypes (FYPO)**	FYPO:0002712	decreased DNA recombination	Enriched	4.32 (10/231)	1.09 (56/5135)	0.00953533	SPAC6G9.13C;SPAC25G10.04C;SPBC409.03;SPBC1718.02;SPAC222.15;SPBC29A10.02;SPAC458.04C;SPCC24B10.14C;SPAC23C4.07;SPBC119.14
**Protein Domains (Pfam)**	PF03303	WTF protein	Enriched	5.19 (12/231)	0.38 (20/5135)	9.9666e-10	SPCC548.03C;SPCC285.07C;SPCC794.02;SPCC1906.04;SPCC1281.08;SPCC285.06C;SPCC622.21;SPCC162.04C;SPCC1450.08C;SPCC1739.15;SPCC970.11C;SPCC553.05C
**Protein Domains (Pfam)**	PF08594	Uncharacterised protein family (UPF0300)	Enriched	1.73 (4/231)	0.11 (6/5135)	0.0037274	SPAC10F6.15;SPAC4G9.07;SPCC1259.14C;SPCC737.04
**Protein Domains (Pfam)**	PF10346	Conidiation protein 6	Enriched	1.29 (3/231)	0.058 (3/5135)	0.00561306	SPAC11D3.01C;SPAC869.09;SPAC22G7.11C
**Protein Domains (Pfam)**	PF03855	M-factor	Enriched	1.29 (3/231)	0.058 (3/5135)	0.00561306	SPBPJ4664.03;SPAC513.03;SPAPB8E5.05

**Transcript Features**		Ribosomal density (rb/kb)	Higher	10.56	4.37	1.58287e-22	SPAC3F10.15C;SPAC16A10.05C;SPAC6G9.13C;SPAPB8E5.05;SPAC3F10.10C;SPAC513.03;SPAC22F3.12C;SPAC1565.04C;SPAC3H1.03;SPAC31G5.09C;SPAC23C4.11;SPAC1783.06C;SPAC15A10.12C;SPAC5H10.11;SPAC3H8.03;SPAC3H1.08C;SPAC18G6.01C;SPAC6F6.05;SPAC24C9.16C;SPAC30C2.05;SPAC806.07;SPAC1A6.08C;SPAC1705.02;SPAC823.02;SPAC1556.04C;SPAC12B10.13;SPAC11D3.01C;SPAC31G5.21;SPAC2F3.17C;SPAC17C9.09C;SPAC144.01;SPBC146.08C;SPBP8B7.28C;SPBC16A3.16;SPBC21B10.02;SPBC12D12.08C;SPBC685.05;SPBC4B4.05;SPBC11B10.06;SPBC29A3.05;SPBC13A2.04C;SPBC1604.10;SPBC29A3.07C;SPBC1711.02;SPBC1711.15C;SPBC4F6.08C;SPBC21C3.04C;SPBC3E7.02C;SPBC32F12.15;SPBC31F10.15C;SPBC27.05;SPBP8B7.02;SPBC800.14C;SPBC4C3.04C;SPBC31E1.03;SPBC409.03;SPBC27B12.10C;SPBC3H7.08C;SPCC1235.13;SPCC622.06C;SPCC285.04;SPCC548.04;SPCC338.12;SPCC162.06C;SPCC777.17C;SPCC338.04;SPCC24B10.05;SPCC24B10.14C;SPCC132.03;SPCC622.07;SPCC162.10
**Transcript Features**		Top 20% shortest transcripts	Enriched	29.00 (67/231)	9.71 (499/5135)	6.60837e-15	SPBC1105.19;SPAC6G9.13C;SPAC23C4.04C;SPAC688.16;SPBPJ4664.03;SPAC31G5.21;SPCPB16A4.07;SPAC11D3.01C;SPAC869.09;SPAC29B12.13;SPBC409.23;SPAC1705.02;SPAC9E9.17C;SPAC513.03;SPAC25H1.10C;SPAC25B8.18;SPBC27.05;SPAC22G7.11C;SPAC6B12.18;SPBC887.16;SPBC21B10.11;SPAC4F10.22;SPBC31F10.15C;SPBC32F12.15;SPAC922.09;SPBC27B12.10C;SPBC15D4.12C;SPBC4C3.04C;SPAC22H10.13;SPAC1B3.20;SPAC806.07;SPBC685.05;SPAC823.02;SPBC1711.02;SPAC23H4.21;SPAC589.08C;SPAC16A10.05C;SPCC1183.12;SPAC869.06C;SPAC2F3.17C;SPBC21B10.02;SPCC338.12;SPAC24C9.16C;SPAC19A8.16;SPBC56F2.14;SPBC23G7.09;SPCC330.21;SPBC4B4.05;SPAC144.01;SPAC30C2.03;SPCC24B10.05;SPBC800.14C;SPAC23C4.11;SPBC23G7.11;SPAC18G6.13;SPAC513.04;SPAC4H3.17;SPCC338.04;SPBC12D12.08C;SPAC1486.11;SPAC22A12.02C;SPBC2A9.14;SPCC285.04;SPAC17G8.15;SPBC32F12.16;SPAC222.17;SPBC1604.10
**Transcript Features**		Top 10% shortest transcripts	Enriched	7.79 (18/231)	2.76 (142/5135)	0.0037274	.16;SPAC4F10.22;SPAC922.09;SPBC15D4.12C;SPAC823.02;SPBC56F2.14;SPCC330.21;SPAC18G6.13;SPAC513.04;SPAC4H3.17;SPAC1486.11;SPAC22A12.02C;SPAC17G8.15
