# Supplementary material for: A PP2A-B55-Mediated Crosstalk between TORC1 and TORC2 Regulates the Differentiation Response in Fission Yeast
Source: Curr Biol. 2017 Jan 23;27(2):175–88. doi: 10.1016/j.cub.2016.11.037 (PMC5266790; doi:10.1016/j.cub.2016.11.037)
Supplement: Document S2. Article plus Supplemental Information [file mmc3.pdf]

Article

Current Biology

A PP2A-B55-Mediated Crosstalk between TORC1 and TORC2 Regulates the Differentiation Response in Fission Yeast

Graphical Abstract

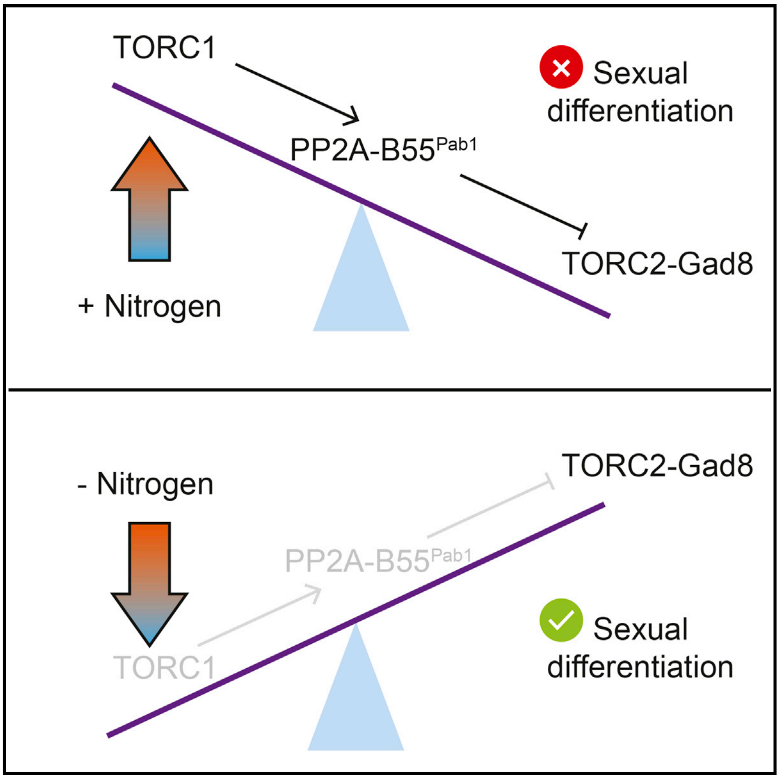

Authors

Ruth Martín, Marina Portantier, Nathalia Chica, Mari Nyquist-Andersen, Juan Mata, Sandra Lopez-Aviles

Correspondence

sandra.lopez-aviles@biotek.uio.no

In Brief

TORC1 and TORC2 play opposite roles in the differentiation response of fission yeast. Here, Martin et al. show that PP2A-$B55^{Pab1}$ coordinates the activities of these two modules in response to nitrogen availability, thus connecting nutritional status to cell-fate decisions.

Highlights

- PP2A-$B55^{Pab1}$ regulates the differentiation response of fission yeast cells
- PP2A-$B55^{Pab1}$ enables a crosstalk between TORC1 and TORC2
- TORC1 favors PP2A-$B55^{Pab1}$ activity to prevent the hyperphosphorylation of Gad8
- TORC1 inactivation leads to PP2A-$B55^{Pab1}$ inhibition, activation of Gad8, and differentiation

Accession Numbers

E-MTAB-4106

Martín et al., 2017, Current Biology 27, 175–188
January 23, 2017 © 2016 The Authors. Published by Elsevier Ltd.
http://dx.doi.org/10.1016/j.cub.2016.11.037

Current Biology
Article

A PP2A-B55-Mediated Crosstalk between TORC1 and TORC2 Regulates the Differentiation Response in Fission Yeast

Ruth Martín,[1,3] Marina Portantier,[1,3] Nathalia Chica,[1] Mari Nyquist-Andersen,[1] Juan Mata,[2] and Sandra Lopez-Aviles[1,4,*]
[1]The Biotechnology Centre of Oslo, University of Oslo, Gaustadalléen 21, Oslo 0349, Norway
[2]Department of Biochemistry, University of Cambridge, Building O, Downing Site, Cambridge CB2 1QW, UK
[3]Co-first author
[4]Lead Contact
*Correspondence: sandra.lopez-aviles@biotek.uio.no
http://dx.doi.org/10.1016/j.cub.2016.11.037

SUMMARY

Extracellular cues regulate cell fate, and this is mainly achieved through the engagement of specific transcriptional programs. The TORC1 and TORC2 complexes mediate the integration of nutritional cues to cellular behavior, but their interplay is poorly understood. Here, we use fission yeast to investigate how phosphatase activity participates in this interplay during the switch from proliferation to sexual differentiation. We find that loss of PP2A-B55^{Pab1} enhances the expression of differentiation-specific genes and leads to premature conjugation. *pab1* deletion brings about a transcriptional profile similar to TORC1 inactivation, and deletion of *pab1* overcomes the repression of differentiation genes in cells overexpressing TORC1. Importantly, we show that this effect is mediated by an increased TORC2-AKT (Gad8) signaling. Under nutrient-rich conditions, PP2A-B55^{Pab1} dephosphorylates Gad8 Ser546, repressing its activity. Conversely, TORC1 inactivation upon starvation leads to the inactivation of PP2A-B55^{Pab1} through the Greatwall-Endosulfin pathway. This results in the activation of Gad8 and the commitment to differentiation. Thus, PP2A-B55^{Pab1} enables a crosstalk between the two TOR complexes that controls cell-fate decisions in response to nutrient availability.

INTRODUCTION

Growing cells integrate a variety of cues in order to decide whether conditions are favorable for cell division or whether they have to halt their cell cycle and differentiate. Failure to do so has negative implications in the fitness of the organism. Notably, cancer pathogenesis is often associated with the poor capacity of cancer cells to differentiate.

The fission yeast *Schizosaccharomyces pombe* provides a good model to study how the nutritional status impinges on the differentiation response. *S. pombe* cells differentiate into conjugation-proficient forms if nutrients are scarce and a mating partner is available. The process of conjugation and meiosis culminates in the formation of spores, which remain dormant until the nutritional conditions improve, when they germinate and resume their mitotic cycle (reviewed in [1]).

Central to these events lies the HMG-box transcription factor Ste11 [2]. Ste11 is essential for the expression of genes implicated in every step of the differentiation pathway [3]. Not surprisingly, Ste11 is subject to a very tight transcriptional regulation. In addition, posttranslational modifications prevent its untimely activation, only allowing it in response to starvation and during G1 phase of the cell cycle (reviewed in [4]).

Sensing whether the environment can provide the elements and the energy required for cell division is an essential aspect of the life cycle of any cell, and, consequently, the signaling pathways conveying this information are highly conserved through evolution. Particularly, target of rapamycin (TOR) signaling plays key roles connecting the environment with the molecular machinery that determines the behavior of the cell (reviewed in [5–7]).

Fission yeast contains two distinct TOR complexes, TORC1 and TORC2, each one with a different catalytic subunit (Tor2 for TORC1 and Tor1 for TORC2) (reviewed in [8]). The best characterized of the two is TORC1, which regulates ribosome biogenesis, protein translation, transcription, and autophagy, as well as cell-cycle progression [9–11]. The second TOR complex is less well understood. However, it is clear that the two TOR complexes have opposite effects in the differentiation response of fission yeast [11, 12], and a crosstalk between the two has been suggested [13].

Compared to protein kinases, the role of protein phosphatases in this context has been poorly explored, although they are excellent candidates to fine-tune cell-fate responses, and they are key players in the making of irreversible decisions during cell-cycle progression [14–16]. Here, we demonstrate that PP2A-B55 acting downstream of TORC1 modulates the activity of the TORC2-Gad8 module in order to prevent its differentiation-promoting functions under nitrogen-rich conditions.

RESULTS

Deletion of *pab1* Results in an Exacerbated Mating Response

PP2A and PP1 are the major phosphatases in the cell, and thus we focused on their potential role in sexual differentiation. To

This is an open access article under the CC BY license (http://creativecommons.org/licenses/by/4.0/).

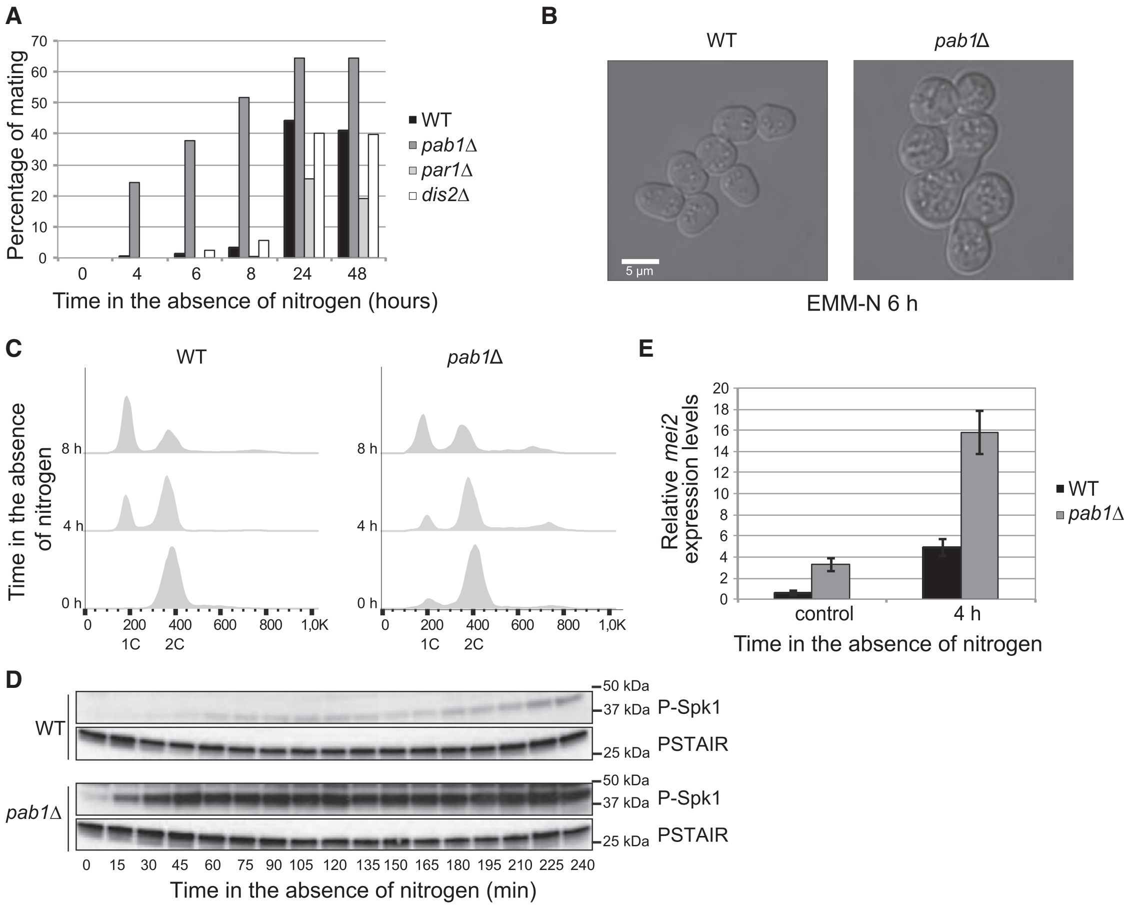

Figure 1. Deletion of *pab1* Results in an Exacerbated Mating Response

(A) Homothallic WT, *pab1*Δ, *par1*Δ, and *dis2*Δ cells were incubated at 25°C in the absence of nitrogen, and their mating ability was determined at 0, 4, 6, 8, 24, and 48 hr.

(B) DIC pictures of homothallic WT and *pab1*Δ cells in the absence of nitrogen for 6 hr.

(C) FACS analysis of the DNA content of homothallic WT and *pab1*Δ cells treated as in (A).

(D) Phosphorylation of the mating pheromone responsive MAPK Spk1 was used as a measure of the pheromone signaling in homothallic WT and *pab1*Δ cells starved for nitrogen. Cdc2 (PSTAIR) served as a loading control.

(E) mRNA expression of *mei2* in homothallic WT and *pab1*Δ cells that had been incubated for 4 hr in EMM (control) or EMM-N. Expression is relative to actin and was determined by qPCR. Mean and SEM of three biological replicates are shown.

See also Figure S1.

investigate the role of PP2A, we used deletion mutants in the genes encoding the two main regulatory subunits, B55 (also known as Pab1) and B56 (also known as Par1), which provide substrate specificity to the complex. For the analysis of PP1, we deleted the gene encoding the main catalytic subunit, Dis2.

Upon nitrogen depletion, homothallic wild-type (WT) cells rapidly initiated the mating response, and the first zygotes could be observed after 8 hr of starvation, with a peak of mating at 24 hr (Figure 1A). *dis2*Δ cells behaved almost indistinguishably to WT, whereas *par1*Δ cells showed a reduced and slower response. Strikingly, in the *pab1*Δ culture, mating products could be observed earlier and to a higher extent than in the WT strain, indicating an exacerbated response in this mutant (Figures 1A and B). This effect was only manifest in this condition (nitrogen starvation) as *pab1*Δ cells mated poorly upon glucose limitation.

Such phenotype could be due to an early G1 arrest upon nitrogen starvation. Cells with a short G2 phase require a longer residence in G1 in order to avoid excessive cell shortening, and therefore such cells are more prone to G1 arrest when nitrogen becomes limiting. PP2A-B55^{Pab1} inhibits entry into mitosis through the dephosphorylation of Wee1 and Cdc25 [17], [14], the two main regulators of Cdk during G2. As a result of a shorter G2 phase, the flow cytometry profile of exponentially proliferating *pab1*Δ cells showed a discrete G1 peak, indicating an expansion of this phase. Despite this, after nitrogen starvation the kinetics of G1 arrest was even slower than in a WT strain (Figure 1C), which might be due to a longer generation time.

In order to evaluate whether the premature mitotic entry could account for the increased conjugation efficiency of the *pab1*Δ strain, we compared it to that of a *wee1*Δ mutant. Similar to *pab1*Δ cells, the flow cytometry profile of *wee1*Δ cells showed a small G1 peak in nutrient-replete conditions. Upon nitrogen depletion, this strain arrested quickly in G1, but this did not correlate with an increased percentage of mating. Actually, its

conjugation efficiency was lower than that of a WT strain (Figure S1), an observation that had been documented before [18]. Hence, we conclude that although a premature mitotic entry prolongs G1 and can accelerate the G1 arrest, this cannot be the only reason for the mating phenotype observed in a *pab1*Δ mutant, especially if this cell-cycle effect occurs via Wee1 inhibition.

Further supporting our hypothesis of an additional, cell-cycle-independent, role of PP2A-B55^{Pab1} during differentiation, the activation of the mating pheromone cascade (as measured by phosphorylation of the MAPK Spk1) in the *pab1*Δ mutant was maximal just after 45 min of nitrogen starvation (Figure 1D). In the case of the WT strain, a faint signal became apparent only after 3 hr of treatment, a time when a large proportion of cells were already arrested in G1. In good agreement with these results, expression of the Ste11 target *mei2* under nitrogen-rich conditions was higher in the *pab1*Δ strain compared to the WT, and it was induced more rapidly and to a higher level upon shift to medium without nitrogen (Figure 1E).

Deletion of *pab1* Leads to Major Transcriptional Changes

Transcriptional adaptation is critical for an adequate mating response. For this reason and given the increased expression of *mei2* observed in *pab1*Δ cells, we decided to explore the transcriptional profile of this strain. Exponentially growing cells lacking *pab1* displayed striking changes in the set of genes that were basally expressed when compared to a WT strain. 240 genes were significantly upregulated at least 3-fold in the mutant, whereas 88 genes were more expressed in the WT strain (Figure 2A). Gene set enrichment analysis of the induced genes revealed over-representation of the GO biological processes "reproduction," "meiotic cell cycle," and "conjugation" among others (Figure 2A; Table S1) and for the gene expression terms "meiosis" and "reproduction modules," "nitrogen depletion genes," and "caffeine and rapamycin induced" (Table S1). Such genome-wide changes in the transcriptional profile could reflect a role of PP2A-B55^{Pab1} modulating the activity of the signaling pathways implicated in the mating response. In particular, given the enrichment in caffeine and rapamycin-induced genes, we paid special attention to genes affected by the two TOR signaling pathways (TORC1 and TORC2). Of the 240 genes upregulated in the *pab1*Δ mutant 63 were also found in the list of genes induced upon TORC1 inactivation (*tor2-ts6*) [10] (Figure 2B). As expected, these common genes showed further enrichment of the terms "Caffeine and rapamycin response," "Conjugation," "Meiosis reproduction module," and "Ste11 targets." Consistently, we could find significant overlap when comparing our list to the set of genes upregulated in a mutant of the SAGA complex component Gcn5, which regulates Ste11 [19] (Figure S2A). Additional comparisons to cell-cycle-regulated genes [20] were also done to rule out an effect of the cell-cycle distribution of our mutant in the expression profile (Figure S2B).

Finally, we compared the list of genes overexpressed in the *pab1*Δ mutant to the list of nitrogen-starvation-induced genes in a *tor1*Δ strain (TORC2 inactive) (Figure 2C). Consistent with previous findings that the two TOR complexes have opposite effects, many of the genes upregulated in *pab1*Δ and that are expressed in the WT strain upon nitrogen depletion depend on Tor1 for their induction, suggesting that the functions of both complexes converge in this response.

PP2A-B55^{Pab1} Functions Downstream of the TORC1 Preventing Sexual Differentiation

Having observed a considerable overlap between genes upregulated upon inactivation of Tor2 and genes upregulated in a *pab1*Δ strain, we decided to analyze in more detail the relationship between these proteins. Overexpression of *tor2* results in a profound mating defect, with no obvious effect on the ability to arrest the cell cycle in G1 upon nitrogen deprivation [9]. Deletion of *pab1* suppressed this defect to a great extent, allowing conjugation of the *tor2* overexpressing cells to the same level as for the WT strain (Figure 3A). Moreover, this correlated with a complete rescue of the expression of *mei2* and with an increased pheromone signaling in this strain (Figures 3B and 3C). These results place PP2A-B55^{Pab1} downstream of TORC1 as an important mediator of its functions. Yet, the fact that conjugation of *nmt1-tor2 pab1*Δ cells was not as high as in the *pab1*Δ mutant suggests that additional effectors of Tor2 also contribute to the regulation of the mating response.

TORC1 is activated by the small GTPase Rhb1, which is, in turn, regulated by the Tsc1-Tsc2 GAP complex [21]. Deletion of *tsc2* impairs the inactivation of TORC1 after nitrogen starvation [22], and it was previously shown that *tsc2* mutants display a mating defect that depends on the cellular concentration at which cells were inoculated on a mating plate [23]. Under our experimental setup, *tsc2*Δ cells completely failed to mate (Figure 3D), and *mei2* expression was utterly abrogated (Figure 3E). Interestingly, we could observe this defect only in the nitrogen starvation response, as this mutant was proficient at mating under low glucose conditions (Figure S3A). Deletion of *pab1* could partially rescue this defect. The mating efficiency of the double mutant *tsc2*Δ *pab1*Δ was still lower compared to the WT strain, but, similar to the single *pab1*Δ mutant, these cells started to conjugate as early as after 4 hr of nitrogen starvation. Basal expression of *mei2* in the *tsc2*Δ *pab1*Δ homothallic strain was as high as in the *pab1*Δ mutant, and it increased upon nitrogen starvation, although not to the same level (Figure 3E). Similarly, we could observe early activation of the pheromone cascade, but it did not increase to the same extent as in the *pab1*Δ mutant (Figure 3F).

The fission yeast Tsc1-Tsc2 complex has been shown to have functions that are independent of TORC1 [24], and early studies suggested that the mating defect of the *tsc2*Δ mutant was the consequence of a deficient pheromone communication [23]. In agreement with a TORC1-independent conjugation defect in *tsc2*Δ cells, we could only marginally rescue the mating defect of this mutant by exogenously inactivating TORC1 with the temperature-sensitive allele *tor2-51* (data not shown).

In order to evaluate whether a faulty pheromone signal transduction could be influencing the mating phenotype of the *tsc2*Δ *pab1*Δ mutant, we analyzed the effect of increasing the concentration of cells in the culture. While high cellular concentration did not have an impact in the mating efficiency of *tsc2*Δ cells, it improved the behavior of the *tsc2*Δ *pab1*Δ mutant, even at concentrations that were deleterious for the WT strain (Figure S3B).

Moreover, we analyzed *mei2* expression in heterothallic WT, *pab1*Δ, *tsc2*Δ, and *tsc2*Δ *pab1*Δ cells, in order to override the

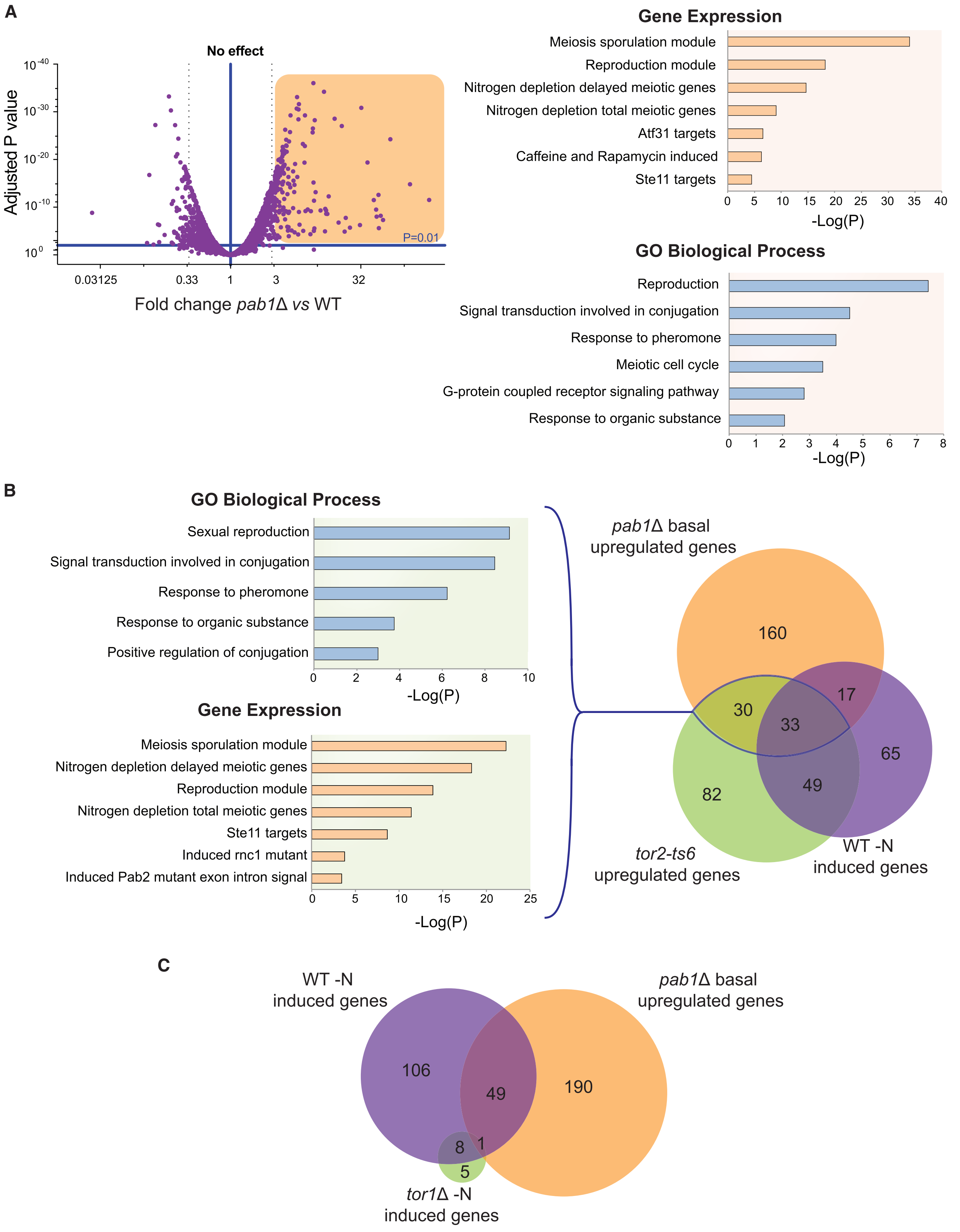

(legend on next page)

induction due to activation of the pheromone pathway. In this background, *mei2* expression was generally lower, but now the double mutant *tsc2*Δ *pab1*Δ and the single *pab1*Δ mutant exhibited the same level of induction (Figure 3G). These results were also confirmed by the observation that heterothallic *tsc2*Δ *pab1*Δ and *pab1*Δ cells accumulated Ste11-GFP in the nucleus to the same extent, both in basal conditions and upon nitrogen depletion (Figure S3C). In WT and *pab1*Δ homothallic strains, Ste11-GFP nuclear accumulation was further enhanced and the cytoplasmic signal was reduced after nitrogen starvation. Nonetheless, the same did not hold true for the homothallic *tsc2*Δ *pab1*Δ mutant that behaved like its heterothallic counterpart (Figure S3C). Therefore, deletion of *pab1* only leads to a full rescue of the defects in *mei2* expression and Ste11 nuclear accumulation of *tsc2*Δ cells if pheromone signaling is prevented. These observations together with the partial rescue of the conjugation defect of the *tsc2*Δ mutant by either *pab1* deletion or TORC1 inactivation are indicative of additional targets of Tsc1/Tsc2 in the regulation of the pheromone signaling.

All in all, we conclude that PP2A-B55^{Pab1} is an instrumental element downstream of the signaling module composed of Tsc1/Tsc2-Rhb1-TORC1 regulating mating.

Nitrogen Starvation Leads to an Increased TORC2-Gad8 Signaling that Depends on the Inactivation of TORC1 and PP2A-B55^{Pab1}

After addressing the link between TORC1 and PP2A-B55^{Pab1}, we next explored the possibility of a crosstalk with the second TOR complex, TORC2, which is essential for the sexual differentiation pathway. While its role in promoting differentiation is central during nutritional stress, it should not take place under optimal growth conditions. Therefore, we hypothesized that nitrogen starvation would stimulate TORC2 signaling in order to favor its mating promoting activities.

To test this idea, we analyzed the level of phosphorylation of the AKT-like kinase Gad8 at Ser546 during nitrogen starvation. Gad8 is the only known effector of TORC2 and its phosphorylation is used as a readout of TORC2 signaling [25]. In a WT strain, the basal phosphorylation was low, and upon medium shift there was an initial drop before it started to increase, becoming maximal after 2 hr (Figures 4A and S4D). At later time points, the phosphorylation diminished, pointing at a transient nature of this signal (likely due to a negative feedback loop [26]). In a *pab1*Δ mutant, however, phosphorylation was already high in nitrogen-rich conditions, suggesting a negative role for PP2A-B55^{Pab1} on the TORC2-Gad8 pathway. Further reinforcing this idea, overexpression of *pab1* completely abrogated the phosphorylation of Gad8 in response to nitrogen starvation (Figure 4B).

To confirm that this increased TORC2-Gad8 signaling in the *pab1*Δ mutant was a direct effect triggered by the loss of PP2A-B55^{Pab1} and not an adaptive mechanism of the cells bearing the *pab1* deletion, we constructed a conditional mutant of *pab1* (*nmt41-miniAID-pab1* [27, 28]). In this strain, addition of thiamine and auxin rapidly led to the almost complete disappearance of B55^{Pab1}, which was accompanied by an increase in the phosphorylation of Gad8 at Ser546, cell shortening, and the induction of *mei2* expression (Figures 4C–4E). Importantly, cell-cycle distribution was not affected in this mutant. If a G1 arrest was induced alongside B55^{Pab1} depletion, expression of *mei2* could be further potentiated (Figures 4F and 4G), indicating that the expansion of this cell-cycle phase in the *pab1*Δ mutant contributes to the transcription of mating genes.

Induction of Gad8 Ser546 phosphorylation prompted by the lack of nitrogen was a direct consequence of the inhibition of TORC1, as inactivation of TORC1 by means of the temperature-sensitive allele *tor2-51* [9] also produced the same effect (Figure 4H). Recently, Sergio Moreno's group has shown that in fission yeast TORC1 promotes the activity of PP2A-B55^{Pab1} through the repression of the PP2A-B55^{Pab1} inhibitory module constituted by the kinase Greatwall and its substrate Endosulfin (Igo1 in fission yeast) [15, 29]. Conversely, inactivation of TORC1 results in the repression of PP2A-B55^{Pab1} (Figure S4A) and accelerated mitotic entry [30]. In budding yeast, the homolog pathway (Rim15-Igo1/2) also controls PP2A-B55 in response to nutritional cues, affecting the expression of quiescence genes [31] and regulating cell-cycle progression through the stabilization of the CDK inhibitor Sic1 [32]. These observations led us to explore whether the Greatwall-Igo1 pathway is also required for the regulation of TORC2-Gad8. In order to address this possibility, we analyzed Gad8 phosphorylation upon inactivation of TORC1 (*tor2-51*) in *igo1*Δ cells. In this case, phosphorylation of Gad8 only marginally increased upon the temperature shift, but it was far from the level achieved in the single *tor2-51* strain. This also correlated with the inability of the double mutant to induce the expression of *mei2* (Figures 4H and 4I). Gad8 phosphorylation was not observed when mitotic entry was induced through Wee1 inactivation or in a WT strain under the same conditions (Figures 4H, 4I, and S4C), ruling out the possibility of its being a consequence of the cell-cycle effect elicited by the inactivation of TORC1 or by the temperature shift.

We then decided to analyze in more detail the kinetics of Gad8 phosphorylation upon TORC1 inhibition. In a WT strain, nitrogen starvation led to a fast drop in TORC1 activity (as judged by

Figure 2. Deletion of *pab1* Leads to Major Transcriptional Changes

(A) Volcano plot graph showing *pab1*Δ transcriptional profile in comparison to WT in nitrogen-rich conditions, the genes upregulated with an adjusted p value <0.01 and fold change >3 are highlighted. In the right panel, enrichment analysis of genes upregulated in *pab1*Δ by GO Biological Process and Gene expression is shown, (p = adjusted p value).

(B) Venn diagram illustrating the overlaps between *pab1*Δ-upregulated genes in nitrogen-rich conditions, WT-induced genes upon 4 hr of nitrogen starvation, and genes upregulated in a *tor2-ts6* strain at restrictive temperature. In both cases, there is significant overlap with a p value = 3.83e^{-25} when comparing to the gene set induced in the WT strain in response to nitrogen starvation, and a p value = 1.75e^{-37} when comparing to the gene set induced in the *tor2-ts6* strain. In the right panel, enrichment analysis of the genes shared in *pab1*Δ and *tor2-ts6* by GO Biological Process and Gene expression (p = adjusted p value).

(C) Venn diagram showing the overlaps between *pab1*Δ-upregulated genes in nitrogen-rich conditions, WT-induced genes, and *tor1*Δ-induced genes upon 4 hr of nitrogen starvation.

See also Figure S2.

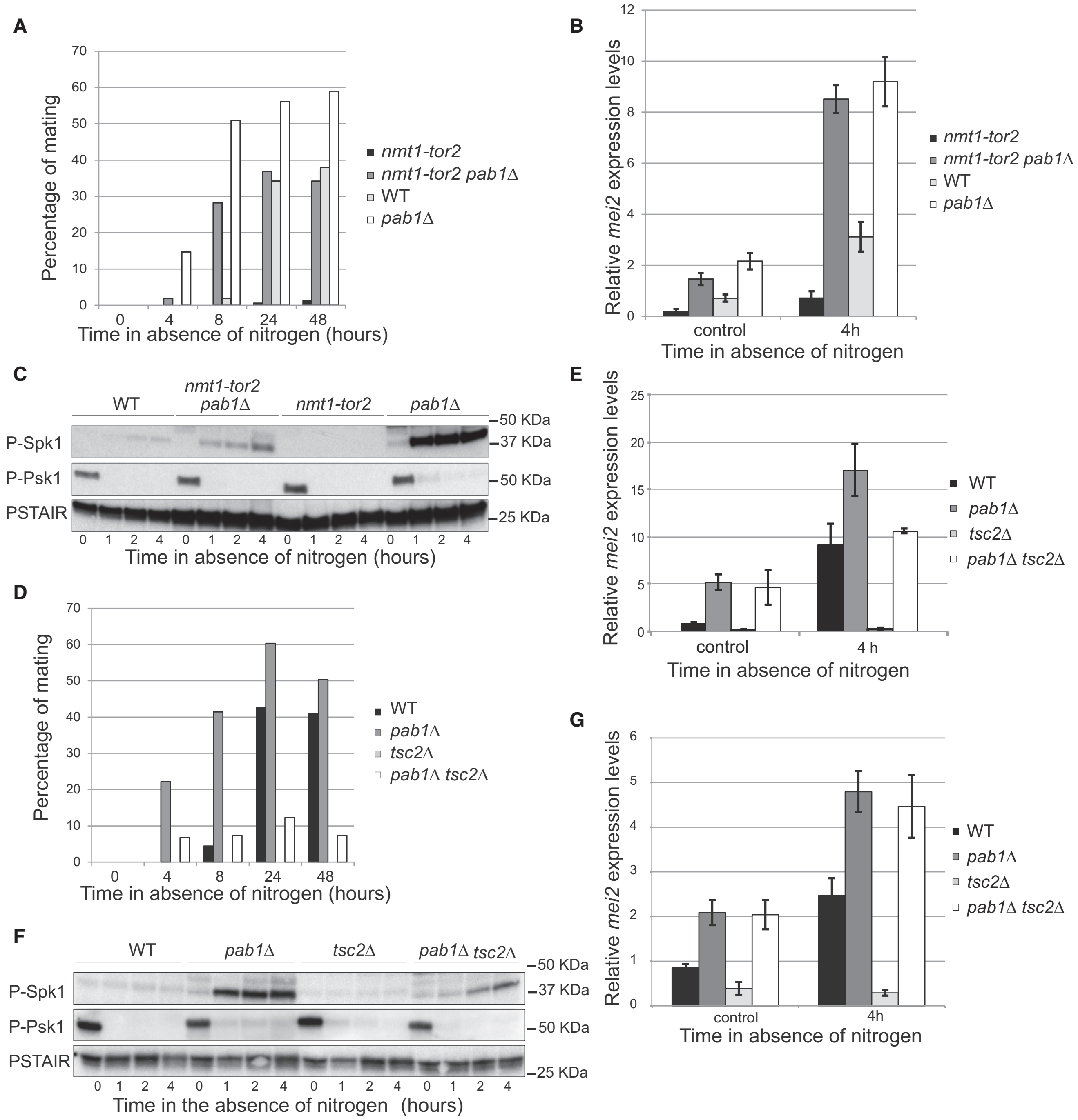

Figure 3. PP2A-B55^{Pab1} Functions Downstream of TORC1 Preventing Sexual Differentiation

(A) Homothallic WT, *pab1Δ*, *nmt1-tor2*, and *nmt1-tor2 pab1Δ* cells that had been grown in the absence of thiamine for 18 hr (to induce the overexpression of *tor2* in the *nmt1-tor2* strains) were incubated at 25°C in the absence of nitrogen, and their mating ability was determined at 0, 4, 8, 24, and 48 hr.

(B) mRNA expression of *mei2* in cells from (A) after incubation for 4 hr in EMM (control) or EMM-N. Expression is relative to actin and was determined by qPCR. Mean and SEM of three biological replicates are shown.

(C) Phosphorylation of the mating pheromone responsive MAPK Spk1 was used as a measure of the pheromone signaling in homothallic WT, *pab1Δ*, *nmt1-tor2*, and *nmt1-tor2 pab1Δ* cells (grown as in A) starved for nitrogen. Phosphorylated Psk1 was used as a measure of TORC1 activity. Cdc2 (PSTAIR) served as a loading control.

(D) Homothallic WT, *pab1Δ*, *tsc2Δ*, and *tsc2Δ pab1Δ* cells were incubated at 25°C in the absence of nitrogen and their mating ability was determined at 0, 4, 8, 24, and 48 hr.

(E) mRNA expression of *mei2* in cells from (D) after incubation for 4 hr in EMM (control) or EMM-N. Expression is relative to actin and was determined by qPCR. Mean and SEM of three biological replicates are shown.

(legend continued on next page)

phosphorylation of the TORC1 substrate Psk1) and to the phosphorylation of Igo1 (Figure S4D). This did not lead to the immediate hyperphosphorylation of Gad8. Actually, Gad8 phosphorylation showed an initial drop before it started to increase and reached a maximal level after 105–120 min of treatment (Figure S4D). TORC2 activity has been shown to decrease in the early time points following nitrogen starvation [26], and this drop in Gad8 phosphorylation might be a reflection of this. In contrast, if the *tor2-51* allele was used to bring about TORC1 inactivation, Gad8 phosphorylation steadily increased as Igo1 phosphorylation became apparent (Figure S4E). Therefore, these experiments indicate that, while PP2A-B55^{Pab1} inhibition following TORC1 inhibition results in the hyperphosphorylation of Gad8, this event is delayed due to the inactivation of TORC2 in the early time points following nitrogen depletion.

All in all, we conclude that TORC1 and PP2A-B55^{Pab1} form a signaling module that prevents premature activation of the TORC2-Gad8 pathway during vegetative growth and that this regulation might explain the exacerbated mating phenotype in a *pab1*Δ strain.

Enhanced TORC2-Gad8 Signaling Contributes to the Premature Differentiation Response in *pab1*Δ Cells

To assess the above-mentioned hypothesis, we took two different approaches. First, we tested whether the constitutively active allele *ryh1QL* (an activator of TORC2 [25]) could rescue the *mei2* expression defect of the *tor2-51 igo1*Δ strain. Indeed, a triple mutant *tor2-51 igo1*Δ *ryh1QL* expressed *mei2* to the same level than the *tor2-51* mutant upon incubation at the restrictive temperature (Figures 5A and 5B).

Next, we investigated the effect of disrupting the TORC2-Gad8 pathway. We did this by treating the cells with the mTOR inhibitor Torin1, which targets both TOR complexes but that, in the context of nitrogen starvation, allowed us to observe the effect of inactivating TORC2, as TORC1 was already inhibited by the lack of nitrogen. Torin1 treatment quickly reduced Gad8 phosphorylation in both the WT and *pab1*Δ strains and completely abrogated the mating ability of both strains (Figures 5C and 5D). This was not a general effect of the drug, as it did not affect the mating ability of a *gcn5*Δ mutant or of a strain overexpressing *ste11* (Figures S5A and S5B). Gcn5 and Ste11 control sexual differentiation by directly modulating the expression of mating genes at the promoter level, and the fact that Torin1 did not inhibit conjugation in these strains indicates that they regulate events downstream of TORC2, as opposed to PP2A-B55^{Pab1}.

In line with this result, mutation of Gad8 Ser546 (*gad8Ser546Ala*) resulted in a decrease in the expression of *mei2* in the *pab1*Δ mutant, and in a major drop in its conjugation efficiency (to the same level as the single *gad8Ser546Ala* mutant) (Figures 5E and 5F). Similarly, a phospho-null mutant of the Gad8 substrate Fkh2 [33] (*fkh2Ser321Ala*) could also reduce *pab1*Δ mating ability to WT levels (Figure 5G). Mutation of Gad8 Ser546 had a stronger effect than mutation of Fkh2 Ser321 in the differentiation response of *pab1*Δ cells. In both cases, however, the effect of the mutation did not completely abrogate the phenotypes associated to *pab1* deletion (either *mei2* induction or mating), suggesting that PP2A-Pab1 and Gad8 regulate other targets involved in the mating response. Yet, the substantial reduction of the conjugation efficiency in the *pab1*Δ *gad8Ser546Ala* argues in favor of the idea that enhanced signaling of the TORC2-Gad8 module is a major contributor to the exacerbated mating phenotype of the *pab1*Δ mutant.

PP2A-B55^{Pab1} Negatively Regulates the Activity of the TORC2-Gad8 Pathway through the Direct Dephosphorylation of Gad8

Having characterized the events involving TORC1, PP2A-B55^{Pab1}, and TORC2-Gad8 from a genetic point of view, we wanted to analyze their interaction using a biochemical approach. Co-precipitation assays showed that PP2A-B55^{Pab1}, Tor1, and Gad8 could be found together in the cell (Figure 6A). PP2A-B55^{Pab1}-GFP localizes throughout the cell and is particularly enriched in the nucleus (Figure S6A), whereas Gad8 has been shown to localize mainly in the cytoplasm [25]. We also observed cytoplasmic localization of Gad8-mCherry, but inhibition of nuclear export with Leptomycin B led to an increased nuclear signal, indicating that a fraction of Gad8 shuttles between the nucleus and the cytoplasm (Figure S6B). While these results still leave open the question of where the interaction between these proteins occurs, they show that Gad8 localization in the cell is dynamic and consistent with its known nuclear functions.

We next addressed the possible mechanisms by which PP2A-B55^{Pab1} could affect the signaling of the TORC2-Gad8 module. We found that PP2A-B55^{Pab1} did not affect the interaction between Gad8 and TORC2, as neither the overexpression nor the depletion of B55^{Pab1} affected the interaction between the TORC2 component Sin1 and Gad8 (Figures 6B and 6C).

However, PP2A-B55^{Pab1} could proficiently reduce the phosphorylation of Gad8 at Ser546 in an in vitro assay, an effect that was reverted by the addition of okadaic acid (Figure 6D). Consistently, deletion of *pab1* also resulted in an enhanced activity of Gad8 toward a recombinant fragment of Fkh2 (Figure 6E). In this assay, Gad8S546A only phosphorylated the substrate feebly, and deletion of *pab1* did not have any significant effect on its activity. Therefore, these experiments confirm our genetic data and strongly suggest that PP2A-B55^{Pab1} can directly repress the activity of the TORC2-Gad8 module through the direct dephosphorylation of Gad8 at Ser546.

DISCUSSION

In fission yeast, nutritional sensing is intimately linked to sexual differentiation. Early studies of the fission yeast counterparts of mTOR, Tor2 as part of TORC1 and Tor1 as part of TORC2, revealed that the two play opposite roles in the mating response

(F) Phosphorylation of the mating pheromone responsive MAPK Spk1 was used as a measure of the pheromone signaling in cells in (D). Phosphorylation of Psk1 served as a readout for TORC1 activity. Cdc2 (PSTAIR) served as a loading control.
(G) mRNA expression of *mei2* in heterothallic WT, *pab1*Δ, *tsc2*Δ, and *tsc2*Δ *pab1*Δ cells treated as in (D) after incubation for 4 hr in EMM (control) or EMM-N. Expression is relative to actin and was determined by qPCR. Mean and SEM of three biological replicates are shown.
See also Figure S3.

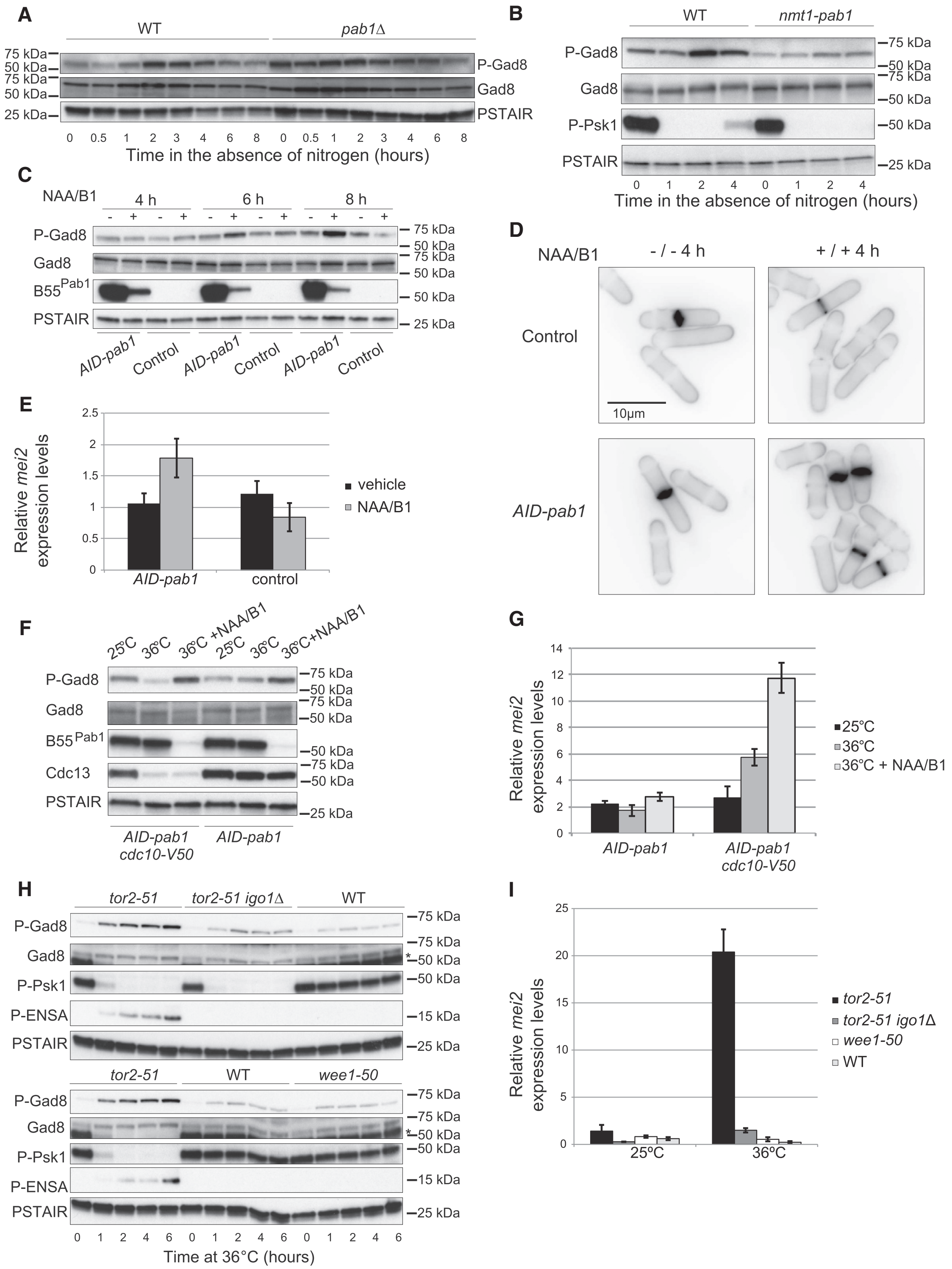

Figure 4. Nitrogen Starvation Leads to an Increased TORC2-Gad8 Signaling that Depends on the Inactivation of TORC1 and PP2A-B55^{Pab1}

(A) Homothallic WT and *pab1*Δ cells were incubated at 25°C in the absence of nitrogen, and samples were collected at the indicated time points. Phosphorylation of Gad8 at Ser546 was followed over the time course by western blot. Total Gad8 and Cdc2 (PSTAIR) served as loading controls.

(B) Homothallic WT and *nmt1-pab1* cells were grown in the absence of thiamine for 18 hr prior to shifting them to minimal medium without nitrogen (EMM-N). Samples were collected at the indicated time points and phosphorylation of Gad8 at Ser546 was monitored by western blot. Phosphorylation of Psk1 served as readout of TORC1 activity and total Gad8 and Cdc2 (PSTAIR) served as loading controls.

(C–E) Control cells (containing the auxin inducible degron background, *Padh15-skp1-At-Tir1-2NLS-Padh15-sk1-Os-Tir1*) and *nmt41-3PK-miniAID-pab1* cells in the same genetic background (referred to as *AID-pab1*) were treated with thiamine (0.5 μM) and NAA (0.5 mM), and samples were collected at the indicated time

(legend continued on next page)

[12]. TORC1 is active when in the presence of a rich nitrogen source, and its activity impedes the expression of genes required for differentiation [9, 10]. On the contrary, TORC2 activity is required for mating and cells deleted for *tor1* or its effector *gad8* are largely sterile [34]. Here, we show that the functions of the two TOR complexes are linked through the protein phosphatase PP2A-B55^{Pab1}.

Genetic studies show that PP2A-B55^{Pab1} lies downstream of TORC1, becoming downregulated as TORC1 activity drops (this work and [30]). Sergio Moreno's group showed that this leads to early mitotic entry due the premature CDK activation and to cell shortening, thus facilitating the extension of G1 [30]. TORC1 inhibition also results in major transcriptional changes and, expectedly, we observed significant overlap between the transcriptional profile of a *pab1*Δ and a *tor2-ts6* allele [10]. Still there was also a considerable amount of non-overlapping expression between our list of genes and the genes induced by TORC1 inactivation in the study carried by Matsuo and colleagues. This discrepancy can be explained from a technical point of view, as the latter study was done using microarrays while we used RNA sequencing, and this is a far more sensitive technology. Moreover, other differences in the experimental design could also have influenced the results. In our case, we analyzed cells permanently deleted for *pab1* grown at 25°C in Edinburgh minimal medium (EMM), while Matsuo et al. grew their cells in YES and transiently inactivated the *tor2-ts6* allele at 34°C.

Importantly, we observed that, as TORC1 becomes inactivated, the activity of the TORC2-Gad8 branch is enhanced through a mechanism that depends on PP2A-B55^{Pab1} inactivation (Figure 7). Nonetheless, during growth on nitrogen-rich medium (when TORC1 and PP2A-B55^{Pab1} are active) not all TORC2-Gad8 activity is affected by PP2A-B55^{Pab1}, as some Gad8-Ser546 phosphorylation can be detected. It was recently shown that glucose activates the TORC2-Gad8 module [35, 36]. As our experiments were done in the presence of glucose, the basal activity that we observe might be a reflection of this regulation. In addition to this, it is possible that only a certain pool of TORC2-Gad8 is subject to PP2A-B55^{Pab1} regulation. TORC2 and Gad8 are required for the cellular response to stress and DNA damage, gene silencing, and telomere maintenance [33, 37–39], but also for the expression of mating genes. This latter activity only has a purpose during nitrogen starvation, when the differentiation program is engaged. Coincidentally, our RNA sequencing (RNA-seq) data revealed that this is the function of Tor1 that becomes enhanced upon loss of PP2A-B55^{Pab1}. Why and how PP2A-B55^{Pab1} regulates only specific functions of the TORC2-Gad8 pathway are outstanding questions, and in the future a thorough characterization of common interactors should help address them.

PP2A-B55^{Pab1} has been mainly studied in the context of cell-cycle regulation, and indeed we observed that loss of *pab1* has a strong effect on cell size and cell-cycle distribution. This on its own does not explain the exacerbated mating phenotype of *pab1*Δ cells, because depletion of B55^{Pab1} could lead to the induction of mating genes even in cells already arrested in G1. Interestingly, we found that a longer residence in G1 was instrumental for the expression of differentiation genes. Hence, downregulation of PP2A-B55^{Pab1} results in a double effect: on the one hand, it favors the G1 arrest; on the other hand, it enhances the activity of TORC2-Gad8 resulting in the expression of mating genes.

In budding yeast, downregulation of PP2A-B55^{Pab1} by the Greatwall-Endosulfin pathway promotes transcription of genes involved in quiescence upon nutritional stress [31], as well as gametogenesis [40]. While the function of budding yeast TORC2 has not been linked to sexual differentiation, overexpression of its substrate YPK2 (homolog to Gad8) induces filamentous growth, which constitutes another form of differentiation that is initiated upon nutritional stress [41].

YPK2 and Gad8 belong to the family of AGC kinases. TORC2 signaling through these kinases is widely conserved across species, with mTORC2 regulating AKT, SGK1, and PKCα. Of the three, Gad8 resembles SGK1 the most. mTORC2 and SGK1 have been involved in the regulation of T cell differentiation [42–44], as well as in adipocyte differentiation [45]. However, SGK1 functions differ depending on the cell type and its overexpression has also been shown to promote cell proliferation and to dictate resistance to anti-AKT therapies in breast cancer cell lines [46]. Importantly, a large cohort study revealed that deletion of *PPP2R2A* (the gene encoding the human B55 alpha subunit of PP2A) was often associated to breast cancer occurrence [47]. Although the mechanisms of action in mammalian cells are undoubtedly more complex, it is most likely that the crosstalk revealed in this work is conserved through evolution and could help explaining the role of PP2A-B55 and SGK1 in these different cellular contexts.

points. (C) Gad8 phosphorylation at Ser546 was monitored by western blot. Depletion of B55^{Pab1} was detected by western blot against its N-terminal 3PK tag. Total Gad8 and Cdc2 (PSTAIR) served as loading controls. (D) Mock-treated and thiamine and NAA-treated cells were stained with Calcofluor to visualize the septa. (E) mRNA expression of *mei2* after 6 hr in the presence of thiamine and NAA. Expression is relative to actin and was determined by qPCR. Mean and SEM of three biological replicates are shown.

(F and G) *nmt41-3PK-miniAID-pab1* and *nmt41-3PK-miniAID-pab1 cdc10-V50* cells were incubated at 36°C and treated with thiamine (0.5 μM) and NAA (0.5 mM) for 6 hr or mock treated. Control cells were kept at 25°C for the same time. (F) Gad8 phosphorylation at Ser546 was monitored by western blot. Depletion of B55^{Pab1} was detected by western blot against its N-terminal 3PK tag. Cdc13 was used to monitor the cell-cycle arrest. Total Gad8 and Cdc2 (PSTAIR) served as loading controls. (G) mRNA expression of *mei2* in cells from (F). Expression is relative to actin and was determined by qPCR. Mean and SEM of three biological replicates are shown.

(H and I) *tor2-51*, *tor2-51 igo1*Δ, WT, and *wee1-50* cells were grown at 25°C (permissive temperature) and then shifted to 36°C in order to inactivate TORC1 and Wee1. Samples were collected at the indicated time points. For comparability between strains, the samples corresponding to the *tor2-51* mutant and the WT strain were run in the two gels. (H) Gad8 phosphorylation at Ser546 and Igo1 phosphorylation at Ser64 (P-ENSA) were detected by western blot. Psk1 phosphorylation was used as readout of TORC1 activity. Total Gad8 and Cdc2 (PSTAIR) served as loading controls. The asterisk in the Gad8 panels indicates remaining signal form the phosphorylated Psk1 western blot. (I) mRNA expression of *mei2* at permissive temperature and after incubation for 4 hr at 36°C. Expression is relative to actin and was determined by qPCR. Mean and SEM of three biological replicates are shown.

See also Figure S4.

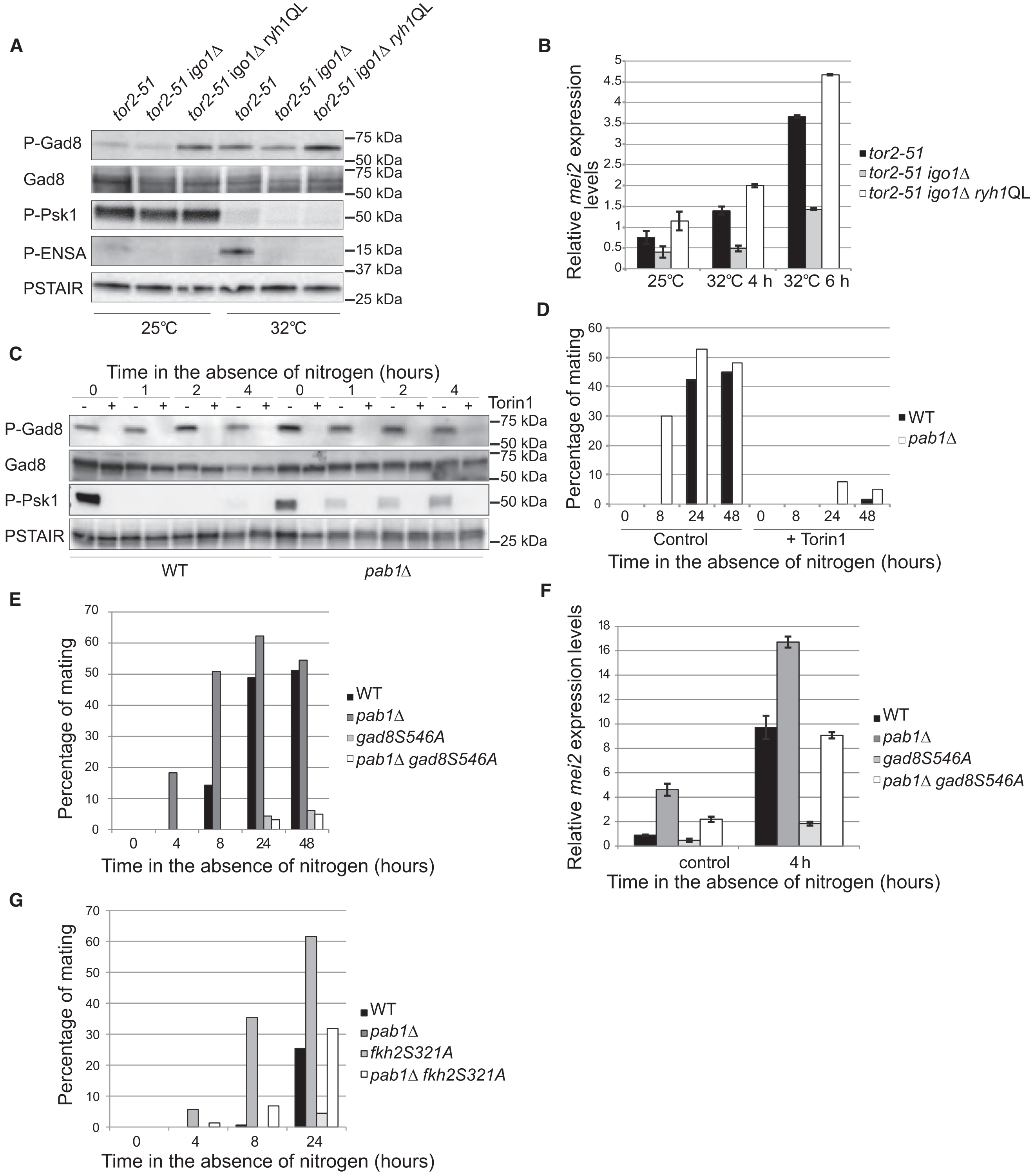

Figure 5. Enhanced TORC2-Gad8 Signaling Contributes to the Premature Differentiation Response in *pab1Δ* Cells

(A and B) *tor2-51*, *tor2-51 igo1Δ*, and *tor2-51 igo1Δ ryh1QL* were incubated at 25°C (permissive temperature) or 32°C for 4 hr in order to inactivate TORC1. (A) Gad8 phosphorylation at Ser546 and Igo1 phosphorylation at Ser64 (P-ENSA) were detected by western blot. Psk1 phosphorylation was used as readout of TORC1 activity. Total Gad8 and Cdc2 (PSTAIR) served as loading controls. (B) mRNA expression of *mei2* at permissive temperature and after incubation for 4 and 6 hr at 32°C. Expression is relative to actin and was determined by qPCR. Mean and SEM of three biological replicates are shown.

(C and D) Homothallic WT and *pab1Δ* cells were incubated at 25°C in the absence of nitrogen and in the presence of Torin1 (25 μM) or in its absence (treated with DMSO instead), and samples were collected at the indicated time points. (C) phosphorylation of Gad8 at Ser546 was monitored by western blot. Phosphorylation

(legend continued on next page)

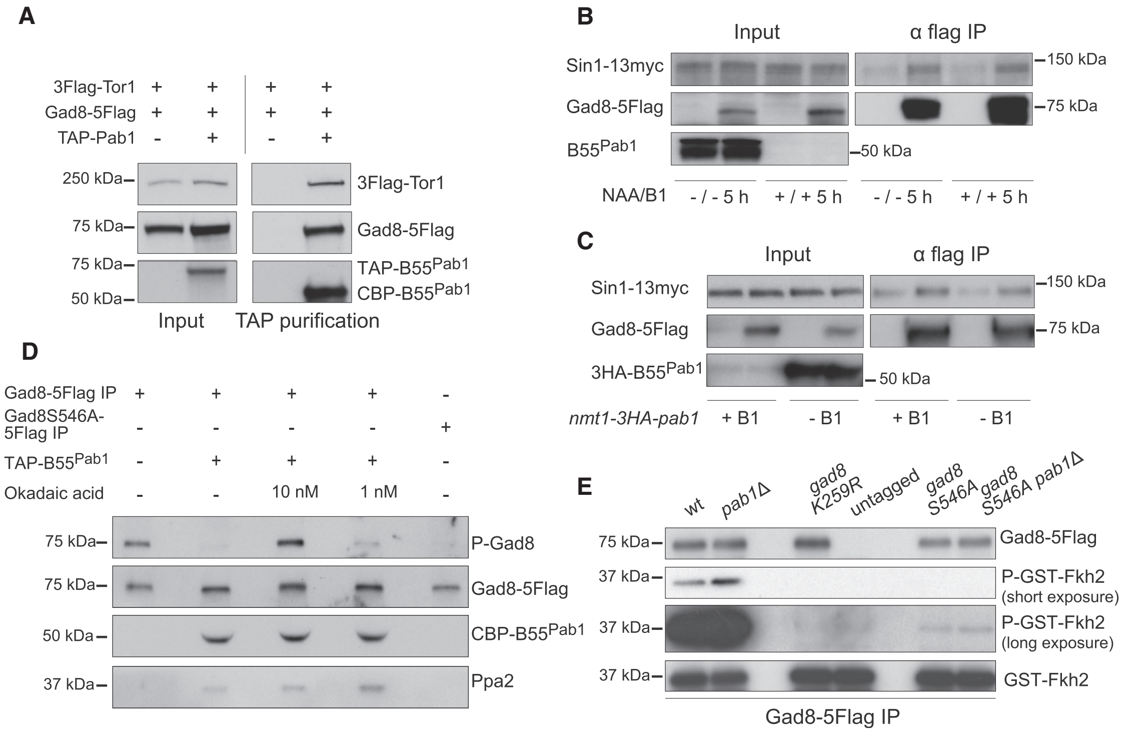

Figure 6. PP2A-B55^{Pab1} Interacts with Tor1 and Gad8 and Directly Dephosphorylates Gad8 at Ser546

(A) N-terminally TAP-tagged B55^{Pab1} was purified from cells expressing flag-tagged versions of Tor1 (3flag-Tor1) and Gad8 (Gad8-5flag). Western blot against the flag tag was used to detect Gad8 and Tor1 that co-purify with Pab1. A strain expressing 3flag-Tor1 and Gad8-5flag was used as a negative control in the TAP-purification. B55^{Pab1} was monitored by western blot against calmodulin-binding peptide (CBP).

(B) *nmt41-3PK-miniAID-pab1 sin1-13myc gad8-5flag* cells and *nmt41-3PK-miniAID-pab1 sin1-13myc* cells (all containing the auxin inducible degron background) were mock treated or treated with thiamine (15 μM) and NAA (0.5 mM) for 5 hr in order to deplete B55^{Pab1}. Gad8 was immunoprecipitated via its C-terminal 5flag tag, and the interaction between Sin1 and Gad8 was subsequently assessed by western blot against the flag tag and the myc tag.

(C) *nmt1-3HA-pab1 sin1-13myc gad8-5flag* cells and *nmt1-3HA-pab1 sin1-13myc* cells were grown in the absence of thiamine for 18 hr in order to induce the overexpression of *pab1*. Gad8 was immunoprecipitated via its C-terminal 5flag tag, and the interaction between Sin1 and Gad8 was subsequently assessed by western blot against the flag tag and the myc tag. Pab1 overexpression was monitored by western blot against the 3HA tag.

(D) PP2A- B55^{Pab1} purified from *par1*Δ cells via an N-terminal TAP tag in B55^{Pab1} was used in a phosphatase assay using as substrate immunopurified Gad8-5flag. Phosphorylation of Gad8 at Ser546 in the reactions was monitored by western blot. Okadaic acid (1 and 10 nM) was added in two of the reactions in order to show that the dephosphorylation observed was PP2A dependent. Immunoprecipitated Gad8 Ser546Ala was also used as control for the non-phosphorylated form of Gad8. Western blots against the flag tag (Gad8), the CBP tag (B55^{Pab1}), and against Ppa2 (the major catalytic subunit of the complex) served as controls for the purifications and showed the even presence of each component in the reactions.

(E) Gad8-5flag or Gad8S546A-5flag was immunopurified from WT and *pab1*Δ cells and used in a kinase assay against a recombinant fragment of Fkh2 (Gln291-Pro370) fused to GST. Gad8K259R-5flag (kinase dead) was used as negative control. Phosphorylation of this fragment was determined by western blot using an antibody that recognizes the phosphorylated AKT consensus sequence (anti-PAS) and was used as readout of Gad8 activity. Western blots against the flag tag (Gad8) and the GST tag (recombinant Fkh2) served as controls for the purifications.

See also Figure S6.

Here, we show that activation of Gad8 results from the repression of PP2A-B55^{Pab1} activity by the Greatwall-Endosulfin pathway. Interestingly, a recent study showed that overexpression of Greatwall brings about the phosphorylation of AKT Ser473 during tumor formation by enhancing the degradation of the phosphatase PHLPP [48]. While the mechanism of action described in the current work is different from this, both works underscore the importance of Greatwall in

of Psk1 served as readout of TORC1 activity. Total Gad8 and Cdc2 (PSTAIR) served as loading controls. (D) The mating counting indicates that Torin1 completely abrogates the mating ability of both WT and *pab1*Δ cells.

(E and F) Homothallic WT, *pab1*Δ, and *pab1*Δ *gad8Ser546Ala* cells grown in EMM were shifted to medium devoid of nitrogen (EMM-N) at 25°C and samples were collected at the indicated time points. (E) mating counting indicates that mutation of Gad8 Ser546Ala completely abrogates the exacerbated conjugation of *pab1*Δ cells. (F) mRNA expression of *mei2* before and upon shift to EMM-N. Expression is relative to actin and was determined by qPCR. Mean and SEM of three biological replicates are shown.

(G) Homothallic WT, *pab1*Δ, *fkh2Ser321Ala*, and *pab1*Δ *fkh2Ser321Ala* cells were incubated at 25°C in the absence of nitrogen, and their mating ability was determined at the indicated time points. The mating counting indicates that mutation of Fkh2 Ser321 to Ala reduces the enhanced mating phenotype of *pab1*Δ cells.

See also Figure S5.

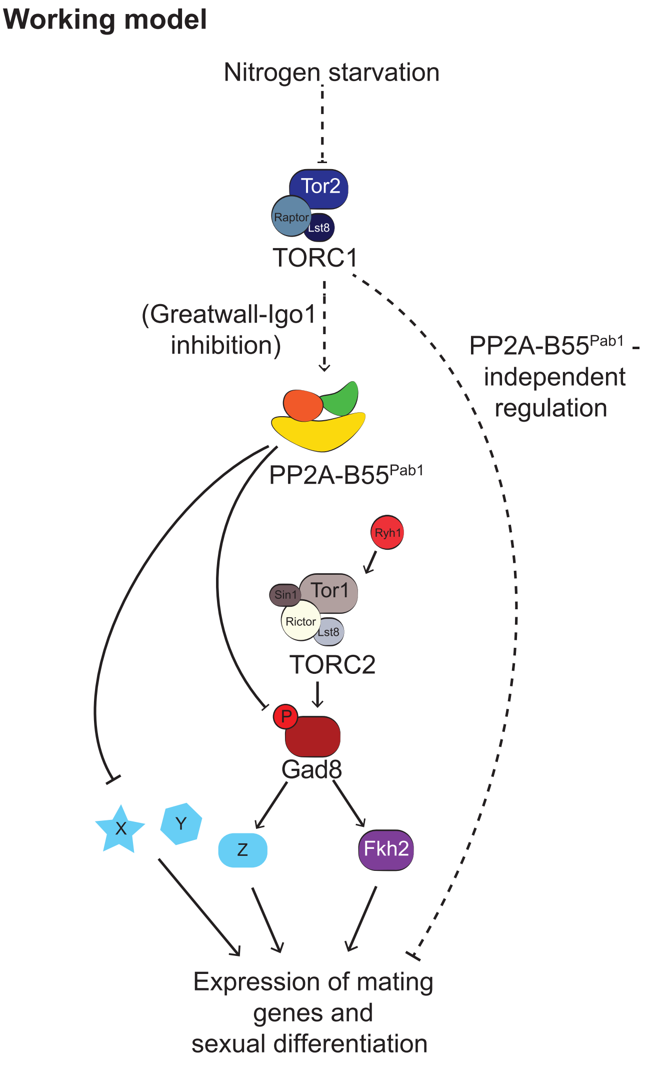

Figure 7. Working Model

PP2A-B55^{Pab1} represses the activity of the module TORC2-Gad8 by directly reverting the phosphorylation of Gad8 at Ser546. In nitrogen-rich conditions, this mechanism prevents untimely expression of mating genes. Upon nitrogen starvation, TORC1 inactivation leads to the inhibition of PP2A-B55^{Pab1}, through a mechanism that depends on the Greatwall-Endosulfin pathway (Ppk18-Igo1). Consequently, PP2A-B55^{Pab1} can no longer exert its negative effect on the TORC2-Gad8 module, which results in the increased phosphorylation of Gad8 at Ser546 and its enhanced activity toward Fkh2Ser321. Phosphorylation of Fkh2 (and probably other unknown targets of Gad8) leads to the induction of the expression of mating genes. Other unknown targets of PP2A-B55^{Pab1} might also participate in the transcriptional regulation of mating genes and sexual differentiation. The relative importance of Gad8 compared to other targets cannot be evaluated with current data. This effect is aided by other events also regulated by TORC1, e.g., cell shortening and G1 arrest (see main text for details).

regulating AKT signaling through the repression of protein phosphatases.

Finally, is TORC2-Gad8 the only target of PP2A-B55^{Pab1} in the mating response? Most likely other targets of PP2A-B55^{Pab1} also influence it. Clearly, the cell-cycle role of PP2A-B55^{Pab1} contributes to it. Moreover, the fact that a *pab1Δ gad8 Ser546Ala* mutant still showed some induction of *mei2* (although its conjugation ability was dramatically reduced) would also indicate it, at least for some aspects of this response. Actually, for a robust effect, one would anticipate that Gad8 and PP2A-B55^{Pab1} share substrates, forming a coherent feedforward loop. In the future, more exhaustive studies of PP2A-B55^{Pab1} and Gad8 targets will be fundamental to understand how these signaling nodes modulate the differentiation process.

EXPERIMENTAL PROCEDURES

Cell Culture and Growth

The strains used in this study are listed in Supplemental Experimental Procedures. All strains were prototroph, and all experiments were performed using early exponential cells grown in EMM containing NH_4Cl 93.5 mM as the source of nitrogen without supplemented amino acids [49]. For nitrogen starvation experiments, cells were collected by filtration and washed with three volumes of EMM without NH_4Cl (EMM-N) before resuspending them in EMM-N. Control cells were washed with EMM and resuspended in fresh EMM.

When using temperature-sensitive alleles, cells were grown at 25°C before shifting them to the restrictive temperature of 36°C in all cases except for the triple mutant *tor2-51 igo1Δ ryh1QL*, in which Tor2 was inactivated at 32°C, as the *ryh1QL* allele does not tolerate the temperature shift to 36°C.

Mating Assays

Mating assays were performed in liquid culture. Briefly, early exponential cells grown at 25°C in EMM were collected by centrifugation and washed with three volumes of EMM-N before resuspending them in EMM-N to a final concentration of 5×10^6 cells/mL. These cultures were subsequently incubated at 25°C, and samples were collected at the indicated time points. After gentle sonication the number of zygotes (Z) and tetrads (T) per 300 non-mating cells (C) were counted and mating efficiency was calculated using the following formula:

$$\frac{(Z+T)\times 2}{(Z+T)\times 2+300}\times 100.$$

Flow Cytometry Analysis

DNA content was determined through fluorescence-activated cell sorting (FACS) analysis of propidium-iodide-stained cells according to published protocols [50].

Cell Imaging

For blankophor staining, 3 mL of cells in exponential phase were washed and resuspended in 3 μL of 50 μg/mL blankophor (Bayer) and 2 μL of PBS. Cell imaging was carried out using a Cell Observer High Speed fluorescence microscope (Zeiss) equipped with a Hamamatsu ORCA-Flash 4.0 camera and a Plan/Apo 100× (numerical aperture [NA] 1.46) oil objective. For fluorescence imaging GFP-specific and m-Cherry specific filters were used. All images were acquired with Zen software and treated using Fiji software (http://fiji.sc/) and Adobe Photoshop CS6 (Adobe Systems).

Genetic Manipulation, Strain Construction, and Plasmids

Gene deletion, promoter exchange, and gene tagging were carried out using PCR cassettes amplified from pFA6a derivative plasmids. More information about plasmid construction, specific phosphomutants, and the NTAP-tagged *pab1* strain can be found in the Supplemental Experimental Procedures. Double mutants were constructed by genetic cross and tetrad dissection.

Additional information on protein and RNA methods can be found under Supplemental Experimental Procedures.

ACCESSION NUMBERS

The accession number for the raw data files reported in this paper is ArrayExpress: E-MTAB-4106.

SUPPLEMENTAL INFORMATION

Supplemental Information includes six figures, Supplemental Experimental Procedures, and one table and can be found with this article online at http://dx.doi.org/10.1016/j.cub.2016.11.037.

AUTHOR CONTRIBUTIONS

R.M., M.P., and N.C. performed experiments. R.M., M.P., M.N.-A., and S.L.-A. generated reagents. S.L.-A., R.M., and M.P. designed experiments. J.M. analyzed the RNA-seq data. All authors contributed to the interpretation of the results. S.L.-A. wrote the manuscript.

ACKNOWLEDGMENTS

We thank Dominique Helmlinger and Sergio Moreno for sharing unpublished results and strains, and for stimulating discussion. We thank Janni Petersen, Ronit Weisman, Kazuhiro Shiozaki, Mitsuhiro Yanagida, and Hisao Masukata for strains, constructs, and antibodies. We thank Toni Hurtado and Beata Grallert for critical reading of the manuscript. This work was supported by NFR FRIMEDBIO grant 214049. M.P. is the recipient of a Kreftforeningen postdoctoral fellowship (grant 5843744). N.C. has received funding from the European Union Seventh Framework Programme (FP7-PEOPLE-2013-COFUND) under grant agreement 609020 - Scientia Fellows. J.M. was supported by BBSRC research grants BB/N007697/1 and BB/M021483/1.

Received: February 5, 2016
Revised: September 20, 2016
Accepted: November 14, 2016
Published: December 29, 2016

REFERENCES

1. Yamamoto, M., Imai, Y., and Watanabe, Y. (1997). 12 Mating and Sporulation in *Schizosaccharomyces pombe*. Cold Spring Harbor Monograph Archive *21C*, 1037–1106.
2. Sugimoto, A., Iino, Y., Maeda, T., Watanabe, Y., and Yamamoto, M. (1991). *Schizosaccharomyces pombe* ste11+ encodes a transcription factor with an HMG motif that is a critical regulator of sexual development. Genes Dev. *5*, 1990–1999.
3. Mata, J., and Bähler, J. (2006). Global roles of Ste11p, cell type, and pheromone in the control of gene expression during early sexual differentiation in fission yeast. Proc. Natl. Acad. Sci. USA *103*, 15517–15522.
4. Anandhakumar, J., Fauquenoy, S., Materne, P., Migeot, V., and Hermand, D. (2013). Regulation of entry into gametogenesis by Ste11: The endless game. Biochem. Soc. Trans. *41*, 1673–1678.
5. Wullschleger, S., Loewith, R., and Hall, M.N. (2006). TOR signaling in growth and metabolism. Cell *124*, 471–484.
6. Laplante, M., and Sabatini, D.M. (2012). mTOR signaling in growth control and disease. Cell *149*, 274–293.
7. Dibble, C.C., and Manning, B.D. (2013). Signal integration by mTORC1 coordinates nutrient input with biosynthetic output. Nat. Cell Biol. *15*, 555–564.
8. Kanoh, J., and Yanagida, M. (2010). Structure of TOR Complexes in Fission Yeast. In The Enzymes, *Volume 27*, M.N. Hall and F. Tamanoi, eds. (Academic Press), pp. 271–284.
9. Alvarez, B., and Moreno, S. (2006). Fission yeast Tor2 promotes cell growth and represses cell differentiation. J. Cell Sci. *119*, 4475–4485.
10. Matsuo, T., Otsubo, Y., Urano, J., Tamanoi, F., and Yamamoto, M. (2007). Loss of the TOR kinase Tor2 mimics nitrogen starvation and activates the sexual development pathway in fission yeast. Mol. Cell. Biol. *27*, 3154–3164.
11. Uritani, M., Hidaka, H., Hotta, Y., Ueno, M., Ushimaru, T., and Toda, T. (2006). Fission yeast Tor2 links nitrogen signals to cell proliferation and acts downstream of the Rheb GTPase. Genes Cells *11*, 1367–1379.
12. Weisman, R., Roitburg, I., Schonbrun, M., Harari, R., and Kupiec, M. (2007). Opposite effects of tor1 and tor2 on nitrogen starvation responses in fission yeast. Genetics *175*, 1153–1162.
13. Ikai, N., Nakazawa, N., Hayashi, T., and Yanagida, M. (2011). The reverse, but coordinated, roles of Tor2 (TORC1) and Tor1 (TORC2) kinases for growth, cell cycle and separase-mediated mitosis in *Schizosaccharomyces pombe*. Open Biol. *1*, 110007.
14. Mochida, S., Ikeo, S., Gannon, J., and Hunt, T. (2009). Regulated activity of PP2A-B55 δ is crucial for controlling entry into and exit from mitosis in *Xenopus* egg extracts. EMBO J. *28*, 2777–2785.
15. Gharbi-Ayachi, A., Labbé, J.-C., Burgess, A., Vigneron, S., Strub, J.-M., Brioudes, E., Van-Dorsselaer, A., Castro, A., and Lorca, T. (2010). The substrate of Greatwall kinase, Arpp19, controls mitosis by inhibiting protein phosphatase 2A. Science *330*, 1673–1677.
16. López-Avilés, S., Kapuy, O., Novák, B., and Uhlmann, F. (2009). Irreversibility of mitotic exit is the consequence of systems-level feedback. Nature *459*, 592–595.
17. Kinoshita, N., Yamano, H., Niwa, H., Yoshida, T., and Yanagida, M. (1993). Negative regulation of mitosis by the fission yeast protein phosphatase ppa2. Genes Dev. *7*, 1059–1071.
18. Wu, L., and Russell, P. (1997). Roles of Wee1 and Nim1 protein kinases in regulating the switch from mitotic division to sexual development in *Schizosaccharomyces pombe*. Mol. Cell. Biol. *17*, 10–17.
19. Helmlinger, D., Marguerat, S., Villén, J., Gygi, S.P., Bähler, J., and Winston, F. (2008). The *S. pombe* SAGA complex controls the switch from proliferation to sexual differentiation through the opposing roles of its subunits Gcn5 and Spt8. Genes Dev. *22*, 3184–3195.
20. Rustici, G., Mata, J., Kivinen, K., Lió, P., Penkett, C.J., Burns, G., Hayles, J., Brazma, A., Nurse, P., and Bähler, J. (2004). Periodic gene expression program of the fission yeast cell cycle. Nat. Genet. *36*, 809–817.
21. Inoki, K., Li, Y., Xu, T., and Guan, K.-L. (2003). Rheb GTPase is a direct target of TSC2 GAP activity and regulates mTOR signaling. Genes Dev. *17*, 1829–1834.
22. Nakashima, A., Sato, T., and Tamanoi, F. (2010). Fission yeast TORC1 regulates phosphorylation of ribosomal S6 proteins in response to nutrients and its activity is inhibited by rapamycin. J. Cell Sci. *123*, 777–786.
23. Matsumoto, S., Bandyopadhyay, A., Kwiatkowski, D.J., Maitra, U., and Matsumoto, T. (2002). Role of the Tsc1-Tsc2 complex in signaling and transport across the cell membrane in the fission yeast *Schizosaccharomyces pombe*. Genetics *161*, 1053–1063.
24. Nakase, Y., Nakase, M., Kashiwazaki, J., Murai, T., Otsubo, Y., Mabuchi, I., Yamamoto, M., Takegawa, K., and Matsumoto, T. (2013). The fission yeast β-arrestin-like protein Any1 is involved in TSC-Rheb signaling and the regulation of amino acid transporters. J. Cell Sci. *126*, 3972–3981.
25. Tatebe, H., Morigasaki, S., Murayama, S., Zeng, C.T., and Shiozaki, K. (2010). Rab-family GTPase regulates TOR complex 2 signaling in fission yeast. Curr. Biol. *20*, 1975–1982.
26. Hálová, L., Du, W., Kirkham, S., Smith, D.L., and Petersen, J. (2013). Phosphorylation of the TOR ATP binding domain by AGC kinase constitutes a novel mode of TOR inhibition. J. Cell Biol. *203*, 595–604.
27. Kanke, M., Nishimura, K., Kanemaki, M., Kakimoto, T., Takahashi, T.S., Nakagawa, T., and Masukata, H. (2011). Auxin-inducible protein depletion system in fission yeast. BMC Cell Biol. *12*, 8.
28. Kubota, T., Nishimura, K., Kanemaki, M.T., and Donaldson, A.D. (2013). The Elg1 replication factor C-like complex functions in PCNA unloading during DNA replication. Mol. Cell *50*, 273–280.
29. Mochida, S., Maslen, S.L., Skehel, M., and Hunt, T. (2010). Greatwall phosphorylates an inhibitor of protein phosphatase 2A that is essential for mitosis. Science *330*, 1670–1673.
30. Chica, N., Rozalén, A.E., Pérez-Hidalgo, L., Rubio, A., Novák, B., and Moreno, S. (2016). Nutritional control of cell size by the Greatwall-Endosulfine-PP2A·B55 pathway. Curr. Biol. *26*, 319–330.
31. Bontron, S., Jaquenoud, M., Vaga, S., Talarek, N., Bodenmiller, B., Aebersold, R., and De Virgilio, C. (2013). Yeast endosulfines control entry

into quiescence and chronological life span by inhibiting protein phosphatase 2A. Cell Rep. *3*, 16–22.

32. Moreno-Torres, M., Jaquenoud, M., and De Virgilio, C. (2015). TORC1 controls G1-S cell cycle transition in yeast via Mpk1 and the greatwall kinase pathway. Nat. Commun. *6*, 8256.
33. Ikeda, K., Morigasaki, S., Tatebe, H., Tamanoi, F., and Shiozaki, K. (2008). Fission yeast TOR complex 2 activates the AGC-family Gad8 kinase essential for stress resistance and cell cycle control. Cell Cycle *7*, 358–364.
34. Matsuo, T., Kubo, Y., Watanabe, Y., and Yamamoto, M. (2003). *Schizosaccharomyces pombe* AGC family kinase Gad8p forms a conserved signaling module with TOR and PDK1-like kinases. EMBO J. *22*, 3073–3083.
35. Cohen, A., Kupiec, M., and Weisman, R. (2014). Glucose activates TORC2-Gad8 protein via positive regulation of the cAMP/cAMP-dependent protein kinase A (PKA) pathway and negative regulation of the Pmk1 protein-mitogen-activated protein kinase pathway. J. Biol. Chem. *289*, 21727–21737.
36. Hatano, T., Morigasaki, S., Tatebe, H., Ikeda, K., and Shiozaki, K. (2015). Fission yeast Ryh1 GTPase activates TOR Complex 2 in response to glucose. Cell Cycle *14*, 848–856.
37. Schonbrun, M., Kolesnikov, M., Kupiec, M., and Weisman, R. (2013). TORC2 is required to maintain genome stability during S phase in fission yeast. J. Biol. Chem. *288*, 19649–19660.
38. Weisman, R., and Choder, M. (2001). The fission yeast TOR homolog, tor1+, is required for the response to starvation and other stresses via a conserved serine. J. Biol. Chem. *276*, 7027–7032.
39. Schonbrun, M., Laor, D., López-Maury, L., Bähler, J., Kupiec, M., and Weisman, R. (2009). TOR complex 2 controls gene silencing, telomere length maintenance, and survival under DNA-damaging conditions. Mol. Cell. Biol. *29*, 4584–4594.
40. Sarkar, S., Dalgaard, J.Z., Millar, J.B.A., and Arumugam, P. (2014). The Rim15-endosulfine-PP2ACdc55 signalling module regulates entry into gametogenesis and quiescence via distinct mechanisms in budding yeast. PLoS Genet. *10*, e1004456.
41. Jin, R., Dobry, C.J., McCown, P.J., and Kumar, A. (2008). Large-scale analysis of yeast filamentous growth by systematic gene disruption and overexpression. Mol. Biol. Cell *19*, 284–296.
42. Heikamp, E.B., Patel, C.H., Collins, S., Waickman, A., Oh, M.-H., Sun, I.-H., Illei, P., Sharma, A., Naray-Fejes-Toth, A., Fejes-Toth, G., et al. (2014). The AGC kinase SGK1 regulates TH1 and TH2 differentiation downstream of the mTORC2 complex. Nat. Immunol. *15*, 457–464.
43. Wu, C., Yosef, N., Thalhamer, T., Zhu, C., Xiao, S., Kishi, Y., Regev, A., and Kuchroo, V.K. (2013). Induction of pathogenic TH17 cells by inducible salt-sensing kinase SGK1. Nature *496*, 513–517.
44. Chang, X., Lazorchak, A.S., Liu, D., and Su, B. (2012). Sin1 regulates Treg-cell development but is not required for T-cell growth and proliferation. Eur. J. Immunol. *42*, 1639–1647.
45. Di Pietro, N., Panel, V., Hayes, S., Bagattin, A., Meruvu, S., Pandolfi, A., Hugendubler, L., Fejes-Tóth, G., Naray-Fejes-Tóth, A., and Mueller, E. (2010). Serum- and glucocorticoid-inducible kinase 1 (SGK1) regulates adipocyte differentiation via forkhead box O1. Mol. Endocrinol. *24*, 370–380.
46. Sommer, E.M., Dry, H., Cross, D., Guichard, S., Davies, B.R., and Alessi, D.R. (2013). Elevated SGK1 predicts resistance of breast cancer cells to Akt inhibitors. Biochem. J. *452*, 499–508.
47. Curtis, C., Shah, S.P., Chin, S.-F., Turashvili, G., Rueda, O.M., Dunning, M.J., Speed, D., Lynch, A.G., Samarajiwa, S., Yuan, Y., et al.; METABRIC Group (2012). The genomic and transcriptomic architecture of 2,000 breast tumours reveals novel subgroups. Nature *486*, 346–352.
48. Vera, J., Lartigue, L., Vigneron, S., Gadea, G., Gire, V., Del Rio, M., Soubeyran, I., Chibon, F., Lorca, T., and Castro, A. (2015). Greatwall promotes cell transformation by hyperactivating AKT in human malignancies. eLife *4*, e10115.
49. Moreno, S., Klar, A., and Nurse, P. (1991). Molecular genetic analysis of fission yeast *Schizosaccharomyces pombe*. Methods Enzymol. *194*, 795–823.
50. Sabatinos, S.A., and Forsburg, S.L. (2009). Measuring DNA content by flow cytometry in fission yeast. Methods Mol. Biol. *521*, 449–461.

Current Biology, Volume *27*

Supplemental Information

A PP2A-B55-Mediated Crosstalk between TORC1 and TORC2 Regulates the Differentiation Response in Fission Yeast

Ruth Martín, Marina Portantier, Nathalia Chica, Mari Nyquist-Andersen, Juan Mata, and Sandra Lopez-Aviles

A

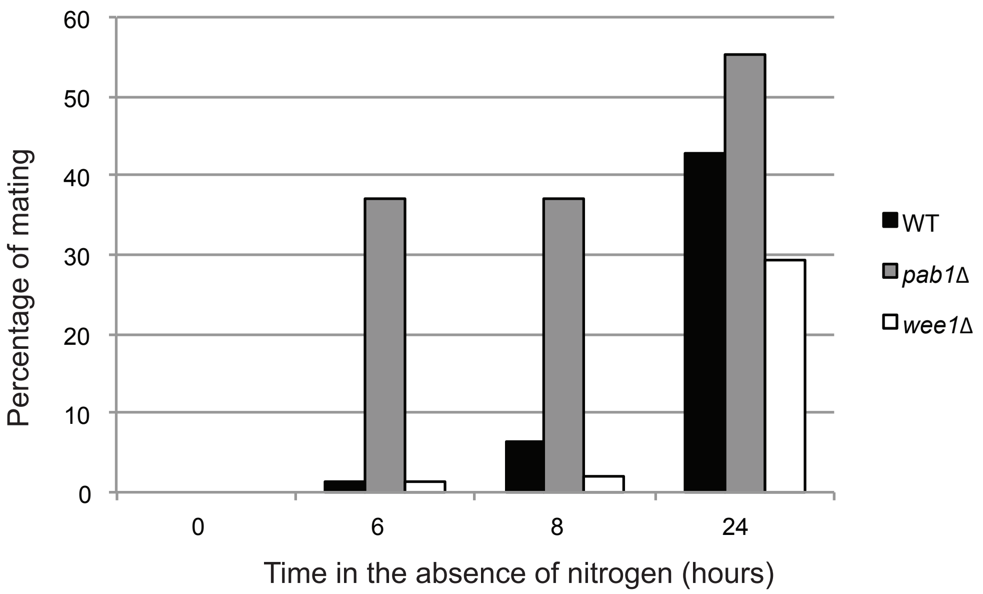

B

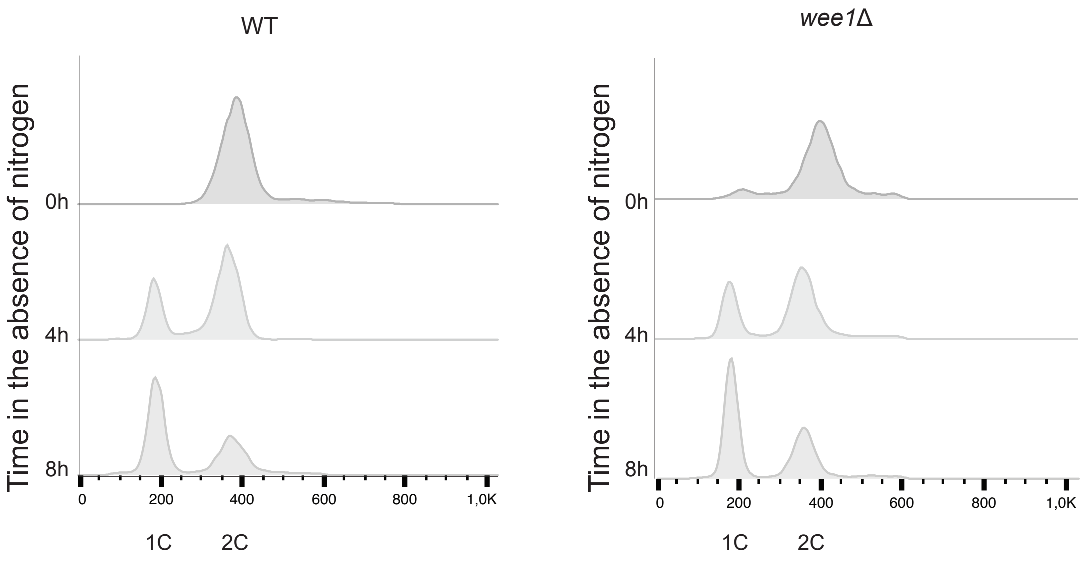

Figure S1 (related to Figure 1): ***wee1*Δ cells display reduced conjugation efficiency.**

A, homotallic WT, *pab1*Δ and *wee1*Δ cells were incubated at 25ºC in the absence of nitrogen and their mating ability was determined at 0 h, 6 h, 8 h and 24 h.
B, FACS analysis of the DNA content of the cells in A.

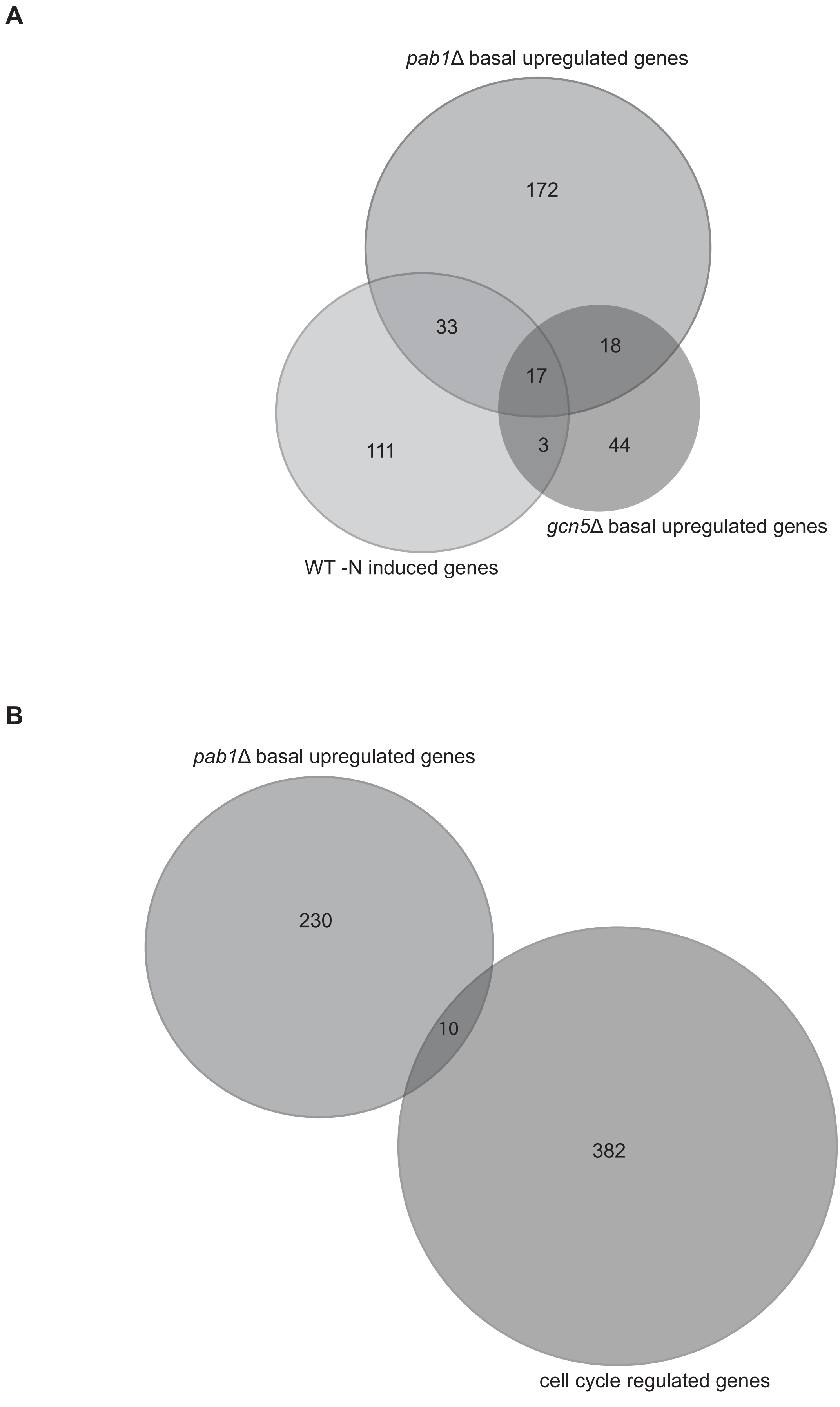

Figure S2 (related to Figure 2): The set of *pab1*Δ upregulated genes significantly overlaps with *gcn5*Δ upregulated genes but not with cell cycle regulated genes.

A, Venn diagram illustrating theoverlap between *pab1*Δ and *gcn5*Δ upregulated gene list in basal conditions (p value = $1.971867e^{-25}$). WT -N induced genes are also shown. **B**, Venn diagram illustrating the overlap between *pab1*Δ upregulated genes in basal conditions and cell cycle regulated gene list (p value = 0.99).

A

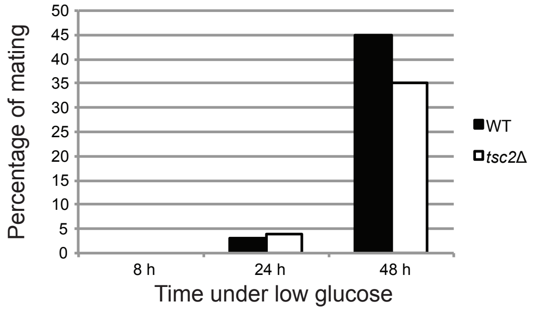

B

	cell concentration								
	$5x10^6$ cells/ml			$50x10^6$ cells/ml			$100x10^6$ cells/ml		
	4 h	24 h	48 h	4 h	24 h	48 h	4 h	24 h	48 h
WT	0	42,7	40,9	0	38	34,2	0	8,5	9
pab1Δ	22,2	60,3	50,33	31,2	60,7	63	42,5	59	56,6
tsc2Δ	0	0	0	0	0	0,6	0	0,6	1,3
pab1Δ tsc2Δ	6,8	12,3	7,4	8,5	16,6	26,1	10,2	13,3	19,4

Percentage of mating (%)

C

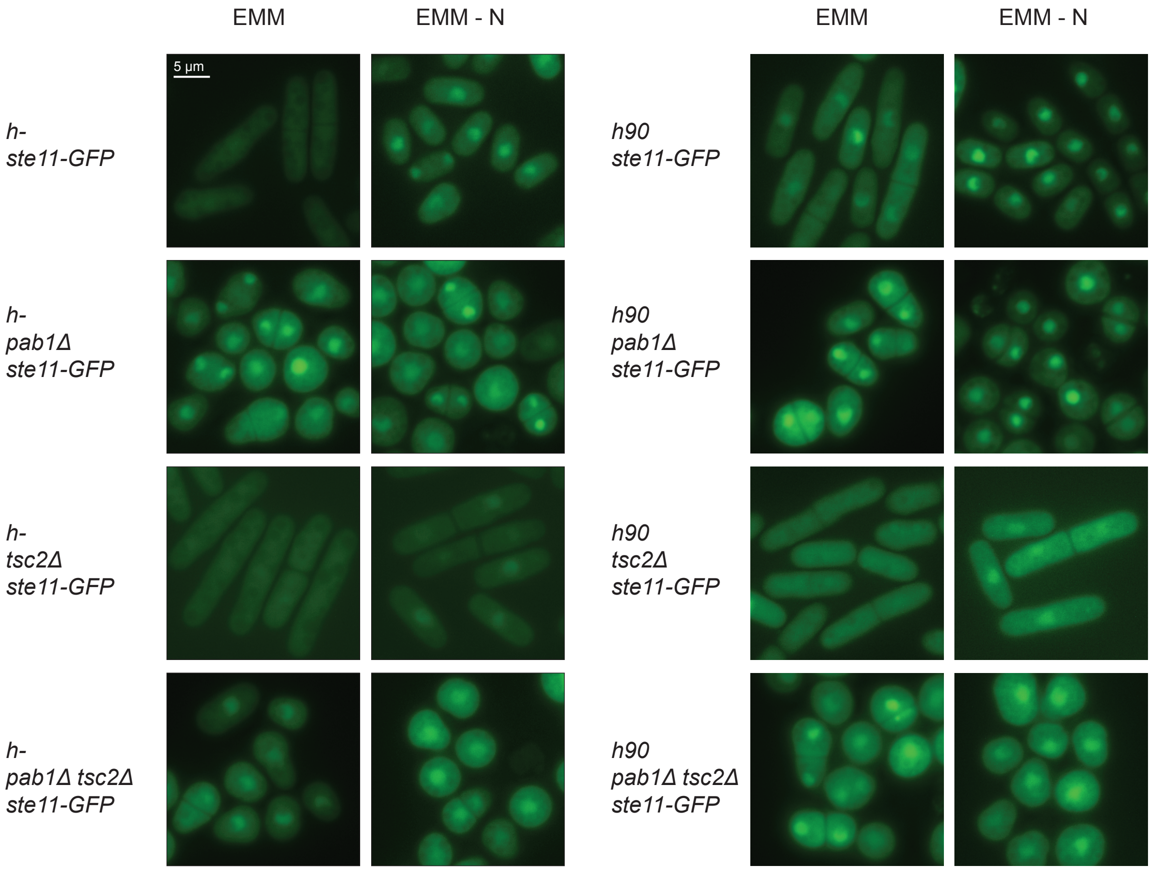

Figure S3 (related to Figure 3): *tsc2*Δ cells mating efficiency under low glucose and rescue of the sexual differentiation defect by deletion of *pab1*.

A, *tsc2*Δ cells show WT conjugation efficiency under low glucose conditions. Homotallic WT and *tsc2*Δ cells were incubated at 25ºC in the presence of 0.1% glucose (supplemented with 3% glycerol) and their mating ability was determined at 8 h, 24 h, and 48 h.

B, The mating efficiency of the *pab1*Δ *tsc2*Δ mutant is enhanced by high cellular concentration. Homotallic WT, *pab1*Δ, *tsc2*Δ and *pab1*Δ *tsc2*Δ cells were incubated at 25ºC in the absence of nitrogen at different cellular concentrations and their mating ability was determined at 4 h, 24 h and 48 h.

C, The defect in Ste11 accumulation of *tsc2*Δ mutants is rescued by *pab1* deletion. Heterotallic and homotallic WT, *pab1*Δ, *tsc2* Δ and *pab1*Δ *tsc2*Δ cells expressing endogenous levels of Ste11-GFP were incubated at 32ºC in the absence of nitrogen for 4h. Control cells were incubated in EMM for the same period of time.

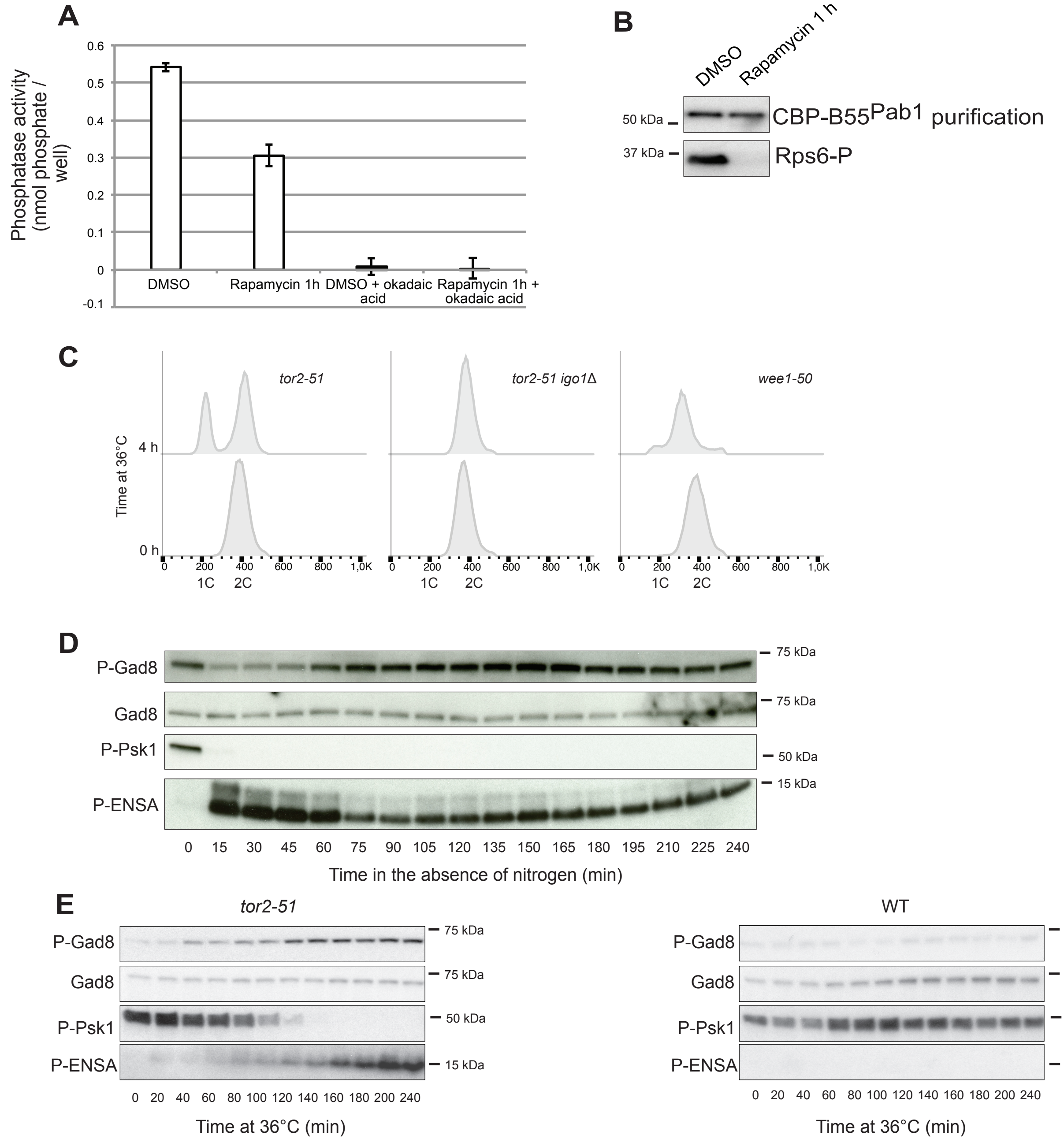

Figure S4 (related to Figure 4): TORC1 inhibition promotes inactivation of PP2A-B55 and this results in increased Gad8 Ser546 phosphorylation.

A, TAP-B55^{Pab1} was purified from cells treated with either DMSO or Rapamycin (0.2 µg/ml) for 1 h and used in a phosphatase assay against a synthetic serine phosphopeptide (DLDVPIPGRFDRRVS(PO3)VAAE). Released phosphate was used as a measure of the phosphatase activity present in the sample, and its concentration was determined based on the absorbance at 620 nm upon reaction with the malachite green reagent. Treatment with okadaic acid completely abrogated the release of phosphate, indicating that the activity observed was PP2A specific. Mean and SEM of two independent biological replicates are shown.

B, Upper panel: B55^{Pab1} present in the samples used in the phosphatase assay was detected by western blot against its amino-terminal CBP-tag. Lower panel: TORC1 activity present in the DMSO and rapamycin treated cultures was determined by western blot against P-Rps6 using TCA extracts.

C, FACS analysis of the DNA content of *tor2-51*, *tor2-51 igo1*Δ and *wee1-50* cells grown at 25°C and incubated at 36°C for 4 h.

D, Homotallic WT cells were incubated at 25°C in the absence of nitrogen and samples were collected at the indicated time points. Gad8 phosphorylation at Ser546 and Igo1 phosphorylation at Ser64 (P-ENSA) were detected by western blot. Psk1 phosphorylation was used as readout of TORC1 activity. Total Gad8 served as loading control.

E, Homotallic *tor2-51* and WT cells were grown at 25°C and then shifted to 36°C in order to inactivate TORC1. Samples were collected at the indicated time points. Gad8 phosphorylation at Ser546 and Igo1 phosphorylation at Ser64 (P-ENSA) were detected by western blot. Psk1 phosphorylation was used as readout of TORC1 activity. Total Gad8 served as loading control.

A

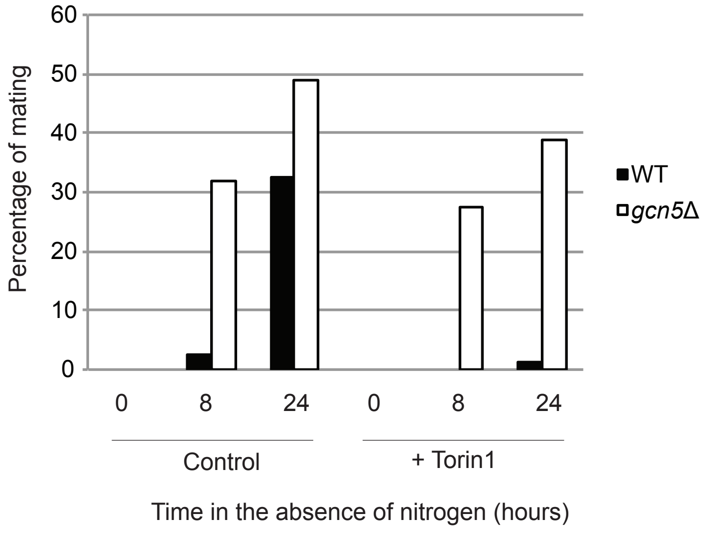

B

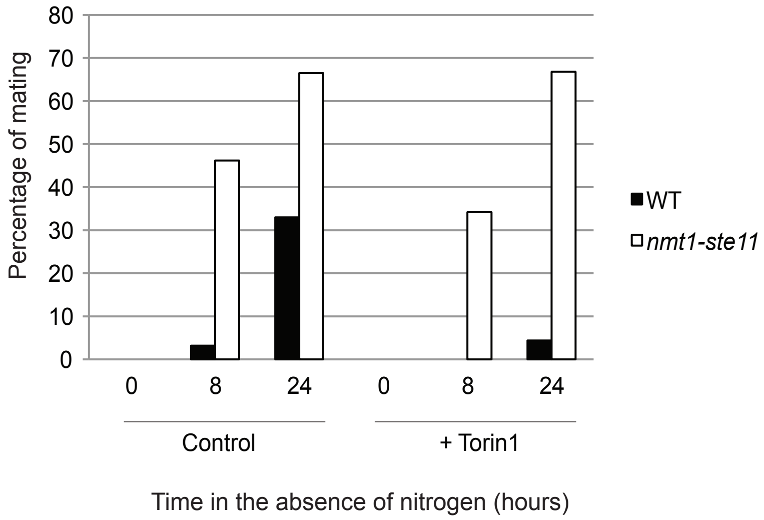

Figure S5 (related to Figure 5): Torin1 does not inhibit mating in a *gcn5*Δ strain or in a strain overexpressing *ste11*.

A, Homotallic WT and *gcn5*Δ cells were incubated at 25ºC in the absence of nitrogen in the presence or in the absence of Torin1 (25 μM) and their mating ability was determined at the indicated time points (as described in Experimental Procedures).

B, Homotallic WT and *nmt1-ste11* cells were grown at 25ºC in the absence of thiamine for 18 h, before they were washed and incubated in the absence of nitrogen in the presence or in the absence of Torin1 (25 μM). Their mating ability was subsequently determined at the indicated time points (as described in Experimental Procedures)

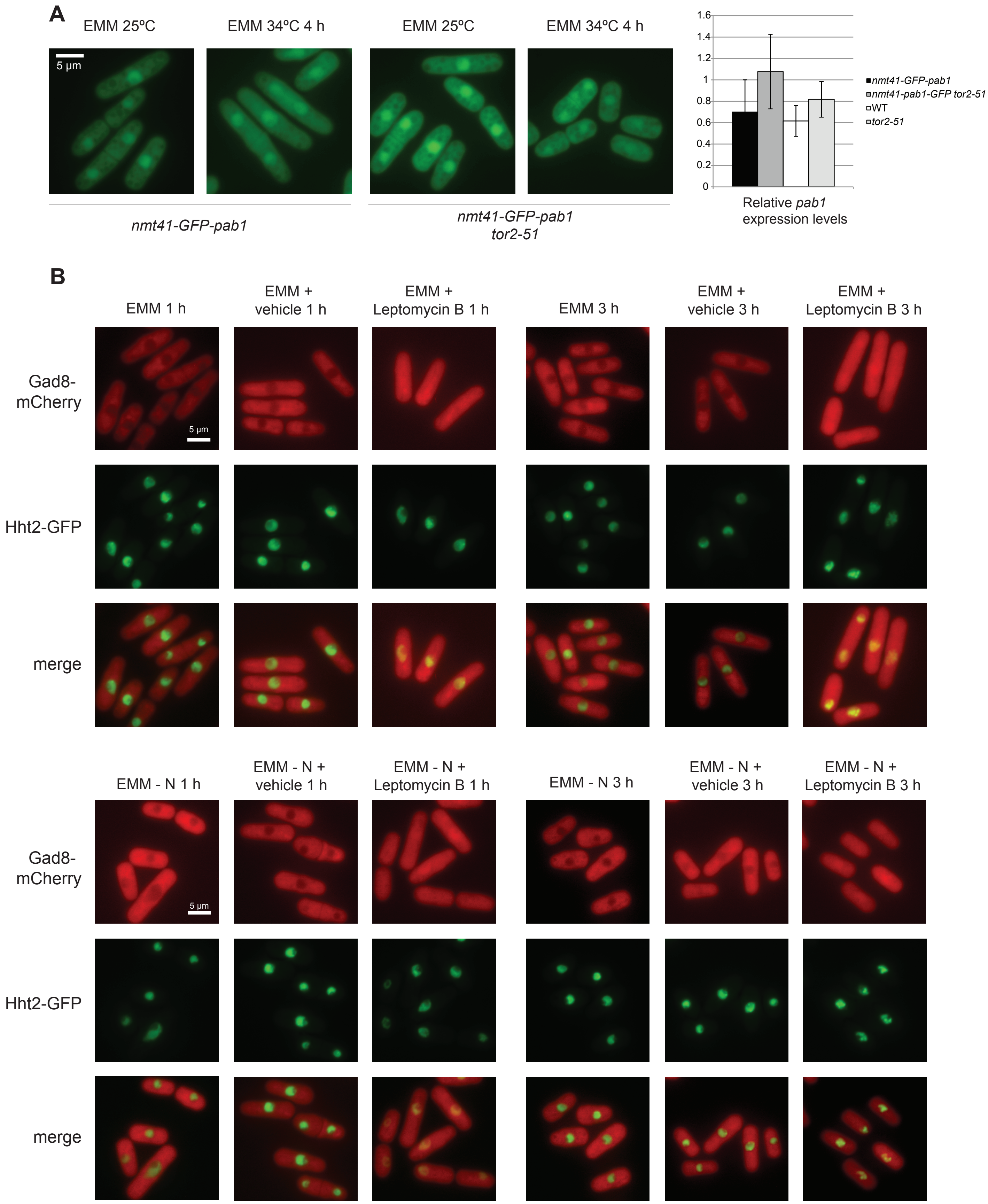

Figure S6 (related to Figure 6): In vivo localization of B55^{Pab1} and of Gad8.

A, Pab1 localizes to the nucleus and cytoplasm and its localization does not change upon Tor2 inactivation.

nmt41-GFP-pab1 and *nmt41-GFP-pab1 tor2-51* cells grown in EMM were shifted to the restrictive temperature of 34ºC for 4 h in order to inactivate Tor2. Control cells were kept at 25ºC for the same period of time. mRNA expression of *pab1* in these cells was analyzed and compared to the endogenous expression in a WT and in a *tor2-51* strain grown in EMM at 25ºC. Expression is relative to actin and was determined by qPCR. Mean and SEM of three biological replicates is shown.

B, Gad8 localizes primarily to the cytoplasm, but treatment with leptomycin B reveals nuclear-cytoplasmic shuttling of a fraction of Gad8.

gad8-mCherry hht2-GFP cells grown at 25ºC in EMM, were split and incubated either in the absence of nitrogen (EMM-N) or in fresh EMM for the indicated times (1 h or 3 h). Gad8 localization under these conditions was assessed upon treatment with leptomycin B or vehicle (for the same period of time). Histone H2 localization served as a marker for the nuclear compartment.

Supplemental Experimental Procedures

Plasmid and strain construction

For the construction of the *nmt41-3PK-miniAID-pab1* strain, a new pFA6a plasmid (pFA6a-KanMX6-p41nmt1-3PK-miniAID) was generated. Briefly, the 3PK tag followed by the miniAID module was amplified by PCR from existing plasmids [S1], adding a 6 Gly linker downstream of the miniAID and overhangs of 15 bp both at the 5' and 3' end of the cassette. These 15 bp overhangs bore homology to the regions upstream and downstream of the tag in a pFA6a-KanMX6-p41nmt1-3HA plasmid. This plasmid was also amplified by PCR using primers matching the 15bp regions of homology, therefore excluding the 3HA tag. The final plasmid was obtained by recombination of these two fragments using a recombination-mediated cloning kit (In-Fusion® Cloning Kit, Takara-Clontech).

For the construction of the *gad8Ser546Ala* strain, the *gad8* gene was first deleted using an Ura4 cassette, in a *ura4-D18* background. The whole genomic sequence spanning from 635 upstream the start codon to 661 bp downstream of the stop codon and comprising the Ser546 to Ala substitution (AGC→GCC) was synthesized by GenScript and cloned in a pUC57 backbone. Digestion of the plasmid with BamHI and PstI released a 3087 bp fragment containing the genomic sequence of *gad8* (Ser546Ala) flanked by the regions upstream of the start codon and downstream of the stop codon. This fragment was then used to transform the *gad8*Δ::*ura4* strain and clones that had exchanged the Ura4 cassette by the mutated allele of *gad8* were selected based on their ability to grow on FOA plates supplemented with Uracil. The presence of the Ser546Ala was confirmed by sequencing.

For the construction of the *gad8-5flag Ser546Ala* allele, genomic DNA was obtained from a Gad8-5flag tagged strain (*gad8-5flag::KanMX6*). This DNA was used as template in two different PCRs. The first PCR amplified a region starting 465 nt upstream of the original stop codon and finishing 8bp downstream of the Ser546 codon. The reverse primer in this PCR introduced a mutation in the Ser546 codon, so that the final PCR product contained the Ser to Ala mutation (AGC→GCC). Similarly, the second PCR produced a fragment that started 9bp before the Ser546 codon, and that finished 374 bp downstream of the 5flag-KanMX6 cassette. As for the first PCR the primer matching the Ser546 codon introduced the mutation to Ala. These two PCRs were then used as template in a tandem PCR reaction together with the forward primer of the first reaction and the reverse primer of the second in order to generate a single cassette now containing the Ser to Ala mutation, the 5flag tag and the kanMX6 module as well as extensive regions of homology to Gad8 ORF and 3' UTR upstream and downstream of the tag and antibiotic resistance. This final PCR was then used to transform a wild type strain. Positive clones were subsequently sequenced to confirm the presence of the mutation.

For the construction of the *fkh2Ser321*Ala strain, the *fkh2* gene was first deleted in an *ura4-D18* background using an Ura4 cassette. The whole genomic sequence spanning from 560 bp upstream the start codon to 400 bp downstream of the stop codon and comprising the Ser321 to Ala substitution (TCC→GCC) was synthesized by GenScript and cloned in a pUC18 backbone. Digestion of the plasmid with AseI released a 3076 bp fragment containing the genomic sequence of *fkh2* (Ser321Ala) flanked by the regions upstream of the start codon and downstream of the stop codon. This fragment was then used to transform the *fkh2*Δ::*ura4* strain and clones that had exchanged the Ura4 cassette by the mutated allele of *fkh2* were selected based on their ability to grow on FOA plates supplemented with Uracil. The presence of the Ser321Ala was confirmed by sequencing.

The NTAP-Pab1 strain was obtained by transformation of *pab1*Δ::KanMX6 *leu1-32* with a pJK148-Ppab1-NTAP-pab1-pab1 3'UTR that had been digested with Sty1 for integration at *leu1*. To obtain the plasmid, the different fragments corresponding to the promoter region and 5'UTR (starting 977 bp upstream of the start codon and finishing at the start codon), the NTAP tag (including a 9 Gly linker) and the genomic sequence and 3'UTR of *pab1* (starting at the start codon and finishing 714 bp downstream of the stop codon) were obtained by PCR. Genomic DNA was used as template to amplify the promoter and the genomic sequence followed by 3'UTR of *pab1*. For the amplification of the NTAP we used a pcDNA3-NTAP plasmid as template. Each PCR reaction was carried out with primers that introduced 15 bp of homology to the adjacent region in the final plasmid. These three PCR reactions were cloned by recombination using a recombination-mediated cloning kit (In-Fusion® Cloning Kit, Takara-Clontech) in a pJK148 empty vector that had been linearized with NotI and SacI.

Strains used in this study

Figure 1		
167	h^{90} WT	Lab stock
343	h^{90} *pab1Δ::kanMX6*	Lab stock
363	h^{90} *par1Δ::kanMX6*	Lab stock
696	h^{90} *dis2Δ::ura4*	Lab stock
Figure 2		
167	h^{90} WT	Lab stock
343	h^{90} *pab1Δ::kanMX6*	Lab stock
678	h^{90} *tor1Δ::kanMX6*	This study
Figure 3		
557	h^{90} *kanMX6::P3nmt1::tor2*	S. Moreno
551	h^{90} *kanMX6::P3nmt1::tor2 pab1Δ::kanMX6*	This study
167	h^{90} WT	Lab stock
343	h^{90} *pab1Δ::kanMX6*	Lab stock
350	h^{90} *tsc2Δ::kanMX6*	This study
554	h^{90} *tsc2Δ::kanMX6 pab1Δ::hphMX6*	This study
147	*h+* WT	Lab stock
411	*h+ pab1Δ::kanMX6*	Lab stock
857	*h- tsc2Δ::kanMX6 pab1Δ::hphMX6*	This study
848	*h- tsc2Δ::kanMX6*	This study
Figure 4		
167	h^{90} WT	Lab stock
343	h^{90} *pab1Δ::kanMX6*	Lab stock
561	h^{90} *kanMX6::P3nmt1::pab1*	Lab stock
594	*h- ade6::ade6+-Padh15-skp1-AtTIR1-2NLS-natMX6-Padh15-skp1-OsTIR1*	Lab stock (Originally from H. Masukata)
593	*h- kanMX6::P41nmt1::3PK-miniAID-pab1 ade6::ade6+-Padh15-skp1-AtTIR1-2NLS-natMX6-Padh15-skp1-OsTIR1*	This study
650	*h- cdc10-V50 kanMX6::P41nmt1::3PK-miniAID-pab1 ade6::ade6+-Padh15-skp1-AtTIR1-2NLS-natMX6-Padh15-skp1-OsTIR1*	This study
222	h^{90} *tor2-51::ura4*	S. Moreno
660	h^{90} *tor2-51::ura4 igo1Δ::hphMX6*	This study
825	h^{90} *wee1-50*	P. Nurse
Figure 5		
222	h^{90} *tor2-51::ura4*	S. Moreno
660	h^{90} *tor2-51::ura4 igo1Δ::hphMX6*	This study
714	h^{90} *tor2-51::ura4 igo1Δ::hphMX6 flag-ryh1QL*	This study (*flag-ryh1QL* allele originally from K. Shiozaki)
167	h^{90} WT	Lab stock
343	h^{90} *pab1Δ::kanMX6*	Lab stock
900	h^{90} *gad8S546A*	This study
908	h^{90} *gad8S546A pab1Δ::hphMX6*	This study
767	h^{90} *fkh2S321A*	This study

773	h^{90} *fkh2S321A pab1Δ::hphMX6*	This study
Figure 6		
841	*h- 3flag-tor1::hphMX6 gad8-5flag::kanMX6 leu1-32*	This study (*3flag-tor1* allele originally from M. Yanagida)
588	*h- 3flag-tor1::hphMX6 gad8-5flag::kanMX6 pab1Δ::kanMX6 leu1-32::leu1::Ppab1::TAP-pab1*	This study
647	*h+ sin1-13myc::natMX6 kanMX6::P41nmt1::3PK-miniAID-pab1 ade6::ade6+-Padh15-skp1-AtTIR1-2NLS-natMX6-Padh15-skp1-OsTIR1*	This study
637	*h- sin1-13myc::natMX6 gad8-5flag::kanMX6 kanMX6::P41nmt1::3PK-miniAID-pab1 ade6::ade6+-Padh15-skp1-AtTIR1-2NLS-natMX6-Padh15-skp1-OsTIR1*	This study
887	*h- sin1-13myc::natMX6 kanMX6::P3nmt1::3HA-pab1*	This study
888	*h+ sin1-13myc::natMX6 gad8-5flag::hphMX6 kanMX6::P3nmt1::3HA-pab1*	This study
732	*h- leu1-32::leu1::Ppab1::TAP-pab1 pab1Δ::kanMX6 Δpar1::hphMX6*	This study
148	*h-* WT	Lab stock
586	*h+ gad8-5flag::kanMX6*	K. Shiozaki
587	*h+ gad8-5flag::kanMX6 pab1Δ::kanMX6*	This study
870	*h- gad8S546A-5flag::kanMX6*	This study
897	*h- gad8S546A-5flag::kanMX6 pab1Δ::hphMX6*	This study
932	*gad8K259R-5flag::hphMX6*	This study (*gad8K259R* allele originally from J. Petersen)
Figure S1		
167	h^{90} WT	Lab stock
343	h^{90} *pab1Δ::kanMX6*	Lab stock
568	h^{90} *wee1Δ::ura4 ura4-D18*	This study
Figure S2		
167	h^{90} WT	Lab stock
343	h^{90} *pab1Δ::kanMX6*	Lab stock
Figure S3		
167	h^{90} WT	Lab stock
350	h^{90} *tsc2Δ::kanMX6*	This study
343	h^{90} *pab1Δ::kanMX6*	Lab stock
554	h^{90} *tsc2Δ::kanMX6 pab1Δ::hphMX6*	This study
849	*h- ste11-GFP::kanMX6*	This study
850	*h- ste11-GFP::kanMX6 tsc2Δ::kanMX6*	This study
851	h^{90} *ste11-GFP::kanMX6 tsc2Δ::kanMX6*	This study
852	h^{90} *ste11-GFP::kanMX6*	This study
853	*h- ste11-GFP::kanMX6 pab1Δ::hphMX6*	This study
854	h^{90} *ste11-GFP::kanMX6 pab1Δ::hphMX6*	This study
855	*h- ste11-GFP::kanMX6 tsc2Δ::kanMX6 pab1Δ::hphMX6*	This study
856	h^{90} *ste11-GFP::kanMX6 tsc2Δ::kanMX6 pab1Δ::hphMX6*	This study
Figure S4		
584	*h- leu1-32::leu1::Ppab1::TAP-pab1 pab1Δ::kanMX6*	This study
167	h^{90} WT	Lab stock

222	h^{90} *tor2-51::ura4*	S. Moreno
660	h^{90} *tor2-51::ura4 igo1Δ::hphMX6*	This study
825	h^{90} *wee1-50*	P. Nurse
Figure S5		
167	h^{90} WT	Lab stock
475	h^{90} *gcn5Δ::kanMX6*	D. Helmlinger
859	h^{90} *kanMX6::P3nmt1::ste11*	This study
Figure S6		
705	*h- kanMX6::P41nmt1::GFP-pab1*	Lab stock
731	*h+ tor2-51::ura4 kanMX6::P41nmt1::GFP-pab1*	Lab stock
905	*h+ gad8-mcherry::kanMX6 hht2-GFP:ura4*	This study

Drugs

A 100 µM stock solution of Okadaic acid (Sigma, 07885) was prepared in DMSO and used at final concentrations 1 nM and 10 nM. A 7.5 mM stock solution of Torin1 (R & D systems, 4247) was prepared in DMSO and used at a final concentration of 25 µM. Rapamycin (Sigma, R0395) was resuspended in DMSO at a concentration of 1mg/ml and used at a final concentration of 0.2 µM. A 500 mM stock solution of 1-Naphthaleneacetic acid potassium salt (ChemCruz Biochemicals, SC-229803) was prepared in water and added to a final concentration of 500 µM.

Leptomycin B (Sigma, L2913) was added at a final concentration of 100 ng/ml. Control cells were treated with the same volume of vehicle (Methanol:water (7:3)).

Protein extraction and Immunoblotting

Samples were collected by filtration and washed in STOP buffer (150 mM NaCl, 50 mM NaF, 10 mM EDTA, 1 mM sodium azide) before freezing them in liquid nitrogen. Protein extracts were prepared using the TCA method [S2]. 30-50 µg of total protein was loaded in Criterion™ TGX™ Any Kd™ gels (BioRad) and transferred to PVDF membranes using a semi-dry blotting system (Trans-Blot® Turbo™ from BioRad). Antibody solutions were prepared in TBS-Tween (0.1%) containing 5% BSA (for all antibodies against phosphorylated residues except for Gad8-Ser546-P) or 5% non-fat milk (in all other instances). Western blots were developed using Amersham ECL western blotting detection reagents.

Antibodies

Polyclonal anti Gad8S546-P and anti Gad8 (directed against the last 20 aminoacids of Gad8) antibodies were produced in rabbit by Eurogentec and used in 1:250-1:1000 dilutions for Western blotting.

The following commercial antibodies were used for WB: anti-PSTAIR (Abcam) 1:1000, anti-phospho S/T AKT substrate (PAS) (Cell Signaling) 1:1000; anti-phospho-S6K (T389) mouse monoclonal antibody (clone # 1A5) (Cell Signaling) 1:1000 was used to detect phosphorylated Psk1; anti phospho-p44/p42 (Cell Signaling) 1:1000 was used to detect phosphorylated Spk1; anti-CBP (GenScript) 1:1000; anti-Myc 9E10 (SIGMA) 1:1000; anti-Flag (SIGMA) 1:1000; anti-HA 12CA5 (Roche) 1:1000; anti-GST (Abcam) 1:1000; anti-V5 (AbD Serotec) 1:1000; anti-PP2A C subunit (Millipore) 1:1000; Phospho-ENSA (Ser67) (Cell Signaling) 1:1000; HRP-conjugated anti mouse IgG (SIGMA) 1:10000 and HRP-conjugated anti rabbit IgG (SIGMA) 1:10000. For IP experiments the anti-Flag, anti-Myc, anti-protein A (SIGMA) antibodies were used in 1:250 dilution. In order to avoid IgG detection in IP experiments, Rabbit and Mouse Trueblot® (Rockland) secondary antibodies were used.

Immunopurifications

150 ml of exponentially growing cells (~10^9 cells) were collected by filtration, and pellets were washed in STOP buffer or in TBS (for IPs used in kinase and phosphatase assays) before freezing them in liquid nitrogen. Native protein extraction was performed in IP Buffer (20 mM Tris-HCl pH 8, 140 mM KCl, 1.8 mM MgCl2, 0.1% NP40, 1 mM PMSF, 1 cOmplete™ mini protease inhibitors (Roche)/5 ml buffer, 1 phosSTOP™ tablet (Roche)/10 ml buffer). Additional phosphatase inhibitors (50 mM NaF, 0,2 mM sodium ortovanadate, 0,02 µM mycrocystin-LR, 1 mM sodium β-glycerophosphate, 10 mM sodium pyrophosphate) were included in the buffer for IPs to be used in kinase assays. For the TAP-purification of PP2A-B55^{Pab1} all phosphatase inhibitors were omitted.

Cells were broken using a FastPrep®-24 equipped with a CryoPrep™ adapter for cryogenic lysis. For all the IPs, 10 mg of whole cell extract were incubated 1 h at 4 ºC with 75 µl of Dynabeds ® Pan Mouse IgG (Invitrogen) which had been incubated overnight with the corresponding antibody in a 1:250 dilution. In the case of Tandem Affinity purifications (TAP) the beads were incubated with anti-protein A. The beads were then washed 4 times in Wash Buffer (20 mM Tris-HCl pH 8, 140 mM KCl, 1.8 mM MgCl2, 0.04% NP40, 10% glycerol) containing 1 mM PMSF and 4 times with Wash Buffer containing 1 mM DTT. The beads were finally resuspended in laemmli loading buffer (for co-inmunopurification experiments) or in the corresponding kinase or phosphatase buffer. In the case of TAP purifications, the beads were resuspended in wash buffer containing DTT and 20 U AcTEV (Invitrogen) was added for 1 h RT in order to release the CBP-tagged protein from the protein A.

Kinase assays

The purification of Gad8-5flag was done in IP Buffer containing phosphatase inhibitors as explained above.

The Gad8-Fkh2 assay was performed according to Laor D et al. [S3]. Briefly, Gad8-5flag IP was incubated with GST-Fkh2 recombinant fragment (100 ng/µl) purified from *E. coli* in the kinase buffer (2 mM DTT, 2.5 mM Mg acetate, 20 mM Tris HCl pH 8, 2.5 mM MgCl2, 1 mM ATP) and incubated 10 min at 30 ºC. Reactions were stopped by boiling the samples for 5 min in laemmli loading buffer. Western blot against PAS was used to detect phosphorylated recombinant Fkh2 and served as readout of the Gad8 activity present in the samples.

Phosphatase assays

For phosphatase assays, CBP-B55^{Pab1} was purified from a *par1*Δ strain (in order to avoid competition for the scaffolding and catalytic subunit) in IP Buffer without phosphatase inhibitors and Gad8-5flag (used as the substrate) was purified from a *pab1*Δ strain with IP Buffer containing phosphatase inhibitors. Prior to the assay, the Gad8-5flag beads were washed in Wash Buffer to ensure removal of phosphatase inhibitors before finally resuspending them in Phosphatase Buffer (50 mM Tris pH 7.5, 10 mM MgCl2, 0.02 % Brij-35, 1 mg/ml BSA). The assay was performed by adding Gad8-5flag IP to eluted CBP-B55^{Pab1} in phosphatase buffer (pre-treated with okadaic acid at the indicated concentration for 15 min in the specified cases) and incubated for 30 min at 30 ºC. The reactions were terminated as explained before. Western blot against Gad8-Ser546-P was used to determine the extent of dephosphorylation of Gad8 by PP2A-B55^{Pab1}.

A malachite green-based colorimetric assay (DuoSet® IC activity assay from R&D systems) was used to determine the activity of PP2A-B55^{Pab1} upon Tor2 inhibition. The assay was performed using CBP-B55^{Pab1} IP according to the manufacturer instructions.

RNA extraction and quantitative PCR (qPCR)

For qPCR and RNAseq experiments 20 ml samples were collected by centrifugation and pellets were washed in DEPC-treated water before freezing them in liquid nitrogen.

Total RNA preparation was performed with MasterPure™ Yeast RNA Purification Kit (Epicentre) following the manufacturer instructions. For qPCR experiments, 1 µg of RNA was used for cDNA synthesis using SuperScript® III Reverse Transcriptase (Invitrogen). qPCR was performed with the corresponding oligos, mei2: AAGAAACTCCCACTGCTGCT and CTGGAGATGATTCAGTGCGT and act1: CAAATCCAACCGTGAGAAGA and CATCACCAGAGTCCAAGACG with SYBR® Select Master Mix (Applied Biosystems). Analysis was done using the ΔΔCt method.

RNAseq

The RNA quality was assessed with Bioanalyzer. The construction of libraries (TruSeq stranded prep) and sequencing (NextSeq 500 75 bp SR H.O) was performed by Norwegian Seq Center http://www.sequencing.uio.no/services/

Reads were aligned to the S. pombe genome using Tophat 2 version 2.1.0 [S4] and Bowtie 2 version 2.2.6 [S5]. The following parameters were used: --no-novel-juncs --no-novel-indels --min-anchor-length 20 --b2-very-fast --read-mismatches 2 --min-intron-length 29 --max-intron-length 819 --max-multihits 1. A gff3 file downloaded from Pombase (ASM294v2.28) [S6] was used as a source of splice junctions. The numbers of reads per feature were quantified using a custom-written Perl script. Determination of differentially expressed genes was performed using the Bioconductor package DEseq [S7]. A threshold if adjusted p values of 0.01 was used. Enrichment analysis was performed using ANGELI [S8]. Raw data files have been deposited in ArrayExpress [S9] with accession number E-MTAB-4106.

The p value of the overlapping genes between lists was calculated with R i386 3.2.2 (hypergeometric test).

Supplemental References

S1. Kubota, T., Nishimura, K., Kanemaki, M. T., and Donaldson, A. D. (2013). The Elg1 replication factor C-like complex functions in PCNA unloading during DNA replication. Mol. Cell *50*, 273–280.

S2. Foiani, M., Marini, F., Gamba, D., Lucchini, G., and Plevani, P. (1994). The B subunit of the DNA polymerase alpha-primase complex in Saccharomyces cerevisiae executes an essential function at the initial stage of DNA replication. Mol. Cell. Biol. *14*, 923–933.

S3. Laor, D., Cohen, A., Pasmanik-Chor, M., Oron-Karni, V., Kupiec, M., and Weisman, R. (2014). Isp7 is a novel regulator of amino acid uptake in the TOR signaling pathway. Mol. Cell. Biol. *34*, 794–806.

S4. Kim, D., Pertea, G., Trapnell, C., Pimentel, H., and Kelley, R. (2013). TopHat2: accurate alignment of transcriptomes in the presence of insertions, deletions and gene fusions. Genome Biology *14,* R36.

S5. Langmead, B., and Salzberg, S. L. (2012). Fast gapped-read alignment with Bowtie 2. Nat. Methods *9*, 357–359.

S6. McDowall, M. D., Harris, M. A., Lock, A., Rutherford, K., Staines, D. M., Bähler, J., Kersey, P. J., Oliver, S. G., and Wood, V. (2015). PomBase 2015: updates to the fission yeast database. Nucleic Acids Res. *43*, D656–61.

S7. Anders, S., and Huber, W. (2010). Differential expression analysis for sequence count data. Genome Biology *11*, R106.

S8. Bitton, D. A., Schubert, F., Dey, S., Okoniewski, M., Smith, G. C., Khadayate, S., Pancaldi, V., Wood, V., and Bähler, J. (2015). AnGeLi: A Tool for the Analysis of Gene Lists from Fission Yeast. Front. Genet. *6*, 76.

S9. Rustici, G., Kolesnikov, N., Brandizi, M., Burdett, T., Dylag, M., Emam, I., Farne, A., Hastings, E., Ison, J., Keays, M., et al. (2013). ArrayExpress update--trends in database growth and links to data analysis tools. Nucleic Acids Res. *41*, D987–90.
